# Dysfunction of a SET3-like complex underlies a family of related neurological disorders

**DOI:** 10.1038/s41467-026-73227-5

**Published:** 2026-05-16

**Authors:** Katie M. Paton, Beatrice Alexander-Howden, Jenna I. Hare, Jacky Guy, Kashyap Chhatbar, Maria Yudina, Stanley T. Morris, Robyn Walls, Tricia Mathieson, Christos Spanos, Adrian P. Bird, Matthew J. Lyst

**Affiliations:** 1 Wellcome Centre for Cell Biology, University of Edinburgh, Michael Swann Building, Max Born Crescent, Edinburgh, EH9 3BF United Kingdom; 2 Simons Initiative for the Developing Brain, Hugh Robson Building, George Square, Edinburgh, EH8 9XF United Kingdom; 3 The Roslin Institute, Easter Bush Campus, Midlothian, EH25 9RH United Kingdom

**Keywords:** Autism spectrum disorders, Transcription, Developmental disorders, Mechanisms of disease

## Abstract

TBLR1 is a subunit of the NCoR corepressor complex that is mutated in a range of neurodevelopmental disorders. Here, we report that TBLR1 functions as a molecular scaffold that physically connects ANKRD11 and SETD5 – two of the most frequently mutated proteins in neurodevelopmental disorders – and links them to the rest of the NCoR complex. The resulting assembly resembles the yeast SET3 complex (SET3C) – a transcriptional regulator. Pathogenic missense mutations in TBLR1, ANKRD11 and SETD5 disrupt this assembly, and an engineered mutation that specifically abolishes SETD5 incorporation into SET3C causes severe developmental impairments in mice. Disruptions of mammalian SET3C components cause highly correlated changes in gene expression – including upregulation of already highly transcribed genes. Together, our results reveal that failure of transcriptional regulation by SET3C is a convergent molecular basis for a family of neurodevelopmental disorders.

## Abstract (web summary)

Mutations in TBLR1, ANKRD11 and SETD5 are common in neurodevelopmental disorders. Here, the authors show that TBLR1 scaffolds a mammalian SET3-like complex linking these factors to NCoR, and that disruption of this transcriptional regulatory assembly underlies convergent neurodevelopmental pathology.

## Introduction

Neurodevelopmental disorders often arise from de novo loss-of-function mutations in single genes. Mutations in any individual gene contribute only a small fraction of total cases, but the cumulative disease burden of lesions in hundreds of different genes is large^1^. Most implicated protein products fall into broad functional categories, including synapse proteins, translational regulators and chromatin proteins^2,3^. In nearly all cases, however, the chain of events linking the mutation to its phenotypic consequences is unclear. Specifically, the extent to which mutated proteins within these categories might function together in shared molecular pathways is not known. This study focuses on the nuclear corepressor (NCoR) complex and, in particular, the subunit TBLR1, encoded by the *TBL1XR1* gene. Mutations in this gene are associated with intellectual disability and developmental delay, but do not cause a single well-defined syndrome. Defined syndromes associated with TBLR1 mutations include West Syndrome, Pierpont Syndrome, Schizophrenia and Cornelia de Lange Syndrome^4–12^. TBL1X, a closely related paralog of TBLR1, is also a subunit of NCoR. Mutations in the *TBL1X* gene appear not to be associated with neurodevelopmental disorders, but instead are linked to hypothyroidism and hearing loss^13,14^.

The first clinically relevant TBLR1-interacting protein to be identified was the methyl-CpG binding protein MeCP2^15–17^, mutations in which cause the severe neurological disorder Rett syndrome (RTT)^18^. RTT is characterised by repetitive hand stereotypies, gait abnormalities, loss of speech and intellectual disability^19–22^. MeCP2 recruits the NCoR complex via a well-defined interaction with the WDR domains of its TBL1X and TBLR1 subunits^23,24^. MeCP2 itself binds broadly across the genome^25^ and evidence suggests that it brings the NCoR complex to chromatin, leading to transcriptional inhibition, in particular of highly methylated genes^26–32^. Supporting the idea that NCoR recruitment to DNA is a critical function of MeCP2^33^, separate clusters of RTT-causing missense mutations disrupt the DNA-binding methyl-CpG binding domain (MBD) or the NCoR interaction domain (NID) of MeCP2^23^. In fact, all characterised RTT-causing mutations in MeCP2 affect the ability of MeCP2 to recruit the TBLR1-containing NCoR complex to chromatin^34^, and radically truncated versions of MeCP2, preserving the MBD and the NID, rescue most of the consequences of MeCP2 deficiency in mice^35^. Given that MeCP2 function relies on its interaction with TBLR1, patient mutations in TBLR1 might be expected to disrupt MeCP2 binding and result in RTT-like symptoms. However, whilst one individual with a clinical diagnosis of RTT has a TBLR1 mutation located at the MeCP2 binding interface^24,36^, the disparate symptoms of most patients overlap only partially with RTT. For this reason, we hypothesised that many pathogenic TBLR1 mutations affect interactions and functions that are MeCP2-independent.

Here, we have investigated the molecular pathology associated with TBLR1 dysfunction. We examined the molecular consequences of pathogenic TBLR1 missense mutations, and found that, whilst many disrupt binding to MeCP2, interactions with SETD5 and/or ANKRD11 were also strongly affected. SETD5 and ANKRD11 have been reported to interact with the NCoR complex^37–40^, and, like MeCP2, are themselves among the ten most frequently mutated genes in neurodevelopmental disorders^41^. Mutations in ANKRD11 cause KBG syndrome (KBGS), which is characterised by intellectual disability, facial dysmorphism, macrodontia, and skeletal abnormalities^42^. Interestingly, the spectra of symptoms for SETD5 syndrome—intellectual disability and dysmorphic facial features—often overlap with those of KBGS. Indeed, some patients diagnosed clinically with KBGS are found to have mutations in SETD5^43^. Motivated by this overlap, and by the effects of disease-causing TBLR1 mutations on ANKRD11/SETD5 binding, we investigated whether TBLR1 might coordinate the functions of these proteins. We found that TBLR1 forms a molecular “bridge” between ANKRD11 and SETD5, resulting in an assembly that, together with the NCoR complex, bears a striking resemblance to the yeast Set3 complex (SET3C)—a transcriptional regulator^44–48^. The relevance of this complex to neurodevelopmental disorders is further supported by our finding that pathogenic missense mutations in both ANKRD11 and SETD5 disrupt their association with SET3C. Moreover, *Setd5*^W834C^, a mutation engineered to specifically disrupt TBLR1 binding, resulted in severe developmental defects in mice, similar to those observed in *Setd5*-*null* animals. In mouse embryonic stem cells, this same mutation, as well as a pathogenic mutation in *Ankrd11*, gives rise to highly correlated changes in gene expression. Furthermore, depletion of SET3C components in a human cultured cell line^49^ resulted in highly correlated changes in gene expression, further implicating SET3C in transcriptional regulation. Intriguingly, following perturbation of human SET3C subunits in this system, the most transcriptionally active genes were found to be preferentially up-regulated. Overall, our results reveal TBLR1 as a keystone of the transcriptional regulator SET3C, whose dysfunction underlies a family of prevalent neurodevelopmental disorders.

## Results

### Pathogenic TBLR1 mutations disturb MeCP2, SETD5 and ANKRD11 binding

**Fig. 1:**
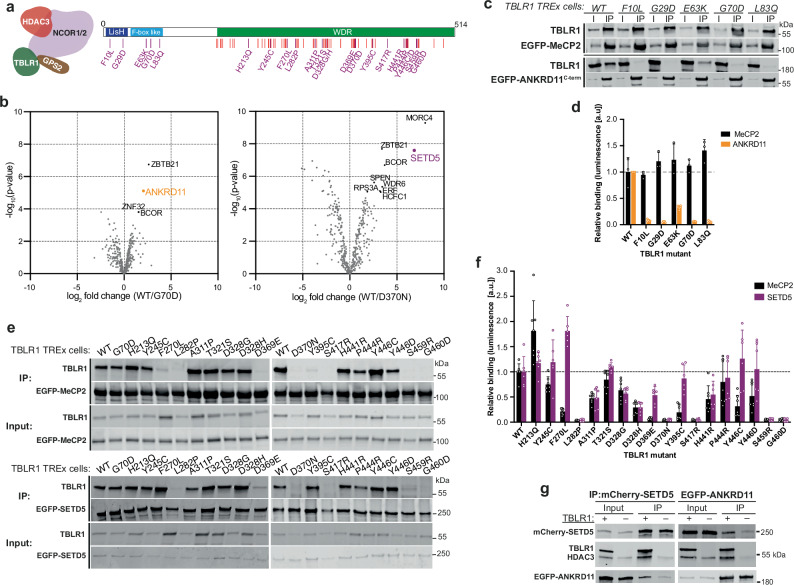
Pathogenic TBLR1 mutations disturb MeCP2, SETD5 and ANKRD11 binding. **a** A schematic of the functional domains in TBLR1. The positions of pathogenic missense mutations from the ClinVar (likely pathogenic/pathogenic), DECIPHER (de novo, excluding benign mutations), and SFARI GENE (de novo) databases are indicated by vertical lines. The mutations we tested (purple) are labelled. Protein domains are annotated. LisH lis homology, WDR WD-repeat. **b** Volcano plots showing enrichment of protein interactions detected by mass spectrometry after immunoprecipitation of wild-type (WT) or mutated (G70D or D370N) TBLR1-mCherry from TBLR1-null Flp-In™ T-REx™ 293 cells (*n* = 3 independent transfections per mutation). Statistical significance was calculated using two-tailed moderated *t*-tests. **c** Western blot for TBLR1 and EGFP following immunoprecipitation of EGFP-ANKRD11^C-term^ (ANKRD11 residues 1762-2663) and EGFP-MeCP2 from the indicated N-terminal TBLR1 mutant Flp-In™ T-REx™-293 cell lines. **d** NanoLuc complementation assay using the indicated N-terminal mutant TBLR1-LgBiT constructs and either MeCP2-SmBiT or ANKRD11-SmBiT (*n* = 3–5 independent transfections per TBLR1 mutant). Mean and ±standard deviation (SD) are indicated. **e** Western blot for TBLR1 and EGFP following immunoprecipitation of EGFP-SETD5 or EGFP-MeCP2 from the indicated WDR domain TBLR1 mutant Flp-In™ T-REx™ 293 cell lines. **f** NanoLuc complementation assay using the indicated WDR domain mutant TBLR1-LgBiT constructs and either MeCP2-SmBiT or SETD5-SmBiT (*n* = 6–7 independent transfections per TBLR1 mutant). Mean and ±SD are indicated. **g** Western blot for TBLR1, HDAC3, mCherry and EGFP following immunoprecipitation of mCherry-SETD5 or EGFP-ANKRD11^C-term^ in either TBLR1-null or TBLR1^WT^ Flp-In™ T-REx™ 293 cells.

To identify interaction partners affected by pathogenic mutations in TBLR1, we used mass spectrometry to compare proteins that co-immunoprecipitate (co-IP) with wild-type and mutant forms of TBLR1. We used CRISPR to inactivate the endogenous TBLR1 gene in Flp-In T-REx 293 cells (Supplementary Fig. 1a) and then transfected wild-type or mutated TBLR1-mCherry expression vectors into these cells. We observed that the G70D mutation^6^, which affects the N-terminal domain of TBLR1—made up of a LisH domain and a putative F-box motif^50^—disturbs the interaction of TBLR1 with ANKRD11 (Fig. 1a, b). In contrast, the D370N^36^ mutation within the C-terminal WDR domain of TBLR1 reduced the interaction with SETD5 (Fig. 1a, b). We decided to focus on ANKRD11 and SETD5, both of which have been reported to interact with the NCoR complex^38–40^, since patients with mutations in either of these genes often present with KBG syndrome or similar neurodevelopmental symptoms^42,43^.

To test whether reduced binding to ANKRD11, SETD5 and/or MeCP2 is a general feature of pathogenic TBLR1 mutations, we created 24 TBLR1 mutant cell lines by integrating TBLR1 cDNAs at the specific FRT site-containing locus in our *TBLR1*-null Flp-In T-REx 293 line (Supplementary Fig. 1a, b). We then transfected these cells with GFP-tagged MeCP2, SETD5 or ANKRD11 and assayed their interaction with TBLR1 mutants by IP with an antibody against GFP followed by western blotting with an antibody against TBLR1. Focussing first on pathogenic missense mutations in the N-terminal domain of TBLR1 (Fig. 1a), all five changes drastically reduced binding to ANKRD11 (Fig. 1c). In contrast, binding to MeCP2, which is known to interact with the C-terminal WDR domain of TBLR1, was unaffected by N-terminal mutations in TBLR1 (Fig. 1c). To validate the effects of TBLR1 mutations on binding to ANKRD11, we used a NanoLuc luciferase protein fragment complementation assay^51,52^. In agreement with the co-IP data, N-terminal TBLR1 mutations strongly reduced binding to ANKRD11, without affecting interaction with MeCP2 (Fig. 1d). The N-terminus of TBLR1 mediates self-association, and also incorporation into the NCoR complex^50^. To test whether pathogenic missense mutations in this region of TBLR1 might affect these interactions, expression plasmids for FLAG-TBLR1 and either wild-type or mutant mCherry-tagged TBLR1 were co-transfected into TBLR1-null cells. IP of wild-type or mutant TBLR1-mCherry, followed by western blotting with FLAG antibodies revealed that TBLR1 self-association was not substantially affected by these mutations (Supplementary Fig. 1c). Moreover, N-terminal mutations in TBLR1 did not markedly reduce TBLR1 association with the NCoR complex, as shown by Western blotting with antibodies against NCoR complex components NCoR1 and HDAC3 (Supplementary Fig. 1c). We conclude that pathogenic mutations in the N-terminus of TBLR1 interfere specifically with its interaction with ANKRD11.

We next assayed the effects of pathogenic missense mutations in the WDR domain of TBLR1 on binding to SETD5 and MeCP2. In co-IP experiments, approximately half of the 18 WDR domain mutations tested strongly reduced binding to both SETD5 and MeCP2 (Fig. 1e), with the effects of mutations on SETD5 binding being well-correlated with their effects on MeCP2 binding. WDR domain mutations, which strikingly reduced binding to SETD5 and/or MeCP2 as assessed by co-IP assays, also showed strong reductions in binding in the NanoLuc complementation assay (Fig. 1f). Furthermore, for all but two of the remaining TBLR1 WDR mutations (H213Q and T321S), the more quantitative NanoLuc assay revealed moderate reductions in binding that were not apparent in the co-IP experiments (Fig. 1f).

As altered stability can contribute to the dysfunction of proteins carrying disease-causing missense mutations^34,53^, we tested the effects of pathogenic TBLR1 mutations on TBLR1 protein levels. When integrated as single-copy transgenes in T-REx cells, all N-terminal mutant forms of TBLR1 were expressed at approximately the same level as the wild-type protein (Supplementary Fig. 1d, e). In most cases, WDR domain mutations did not substantially affect TBLR1 expression levels either. However, we observed up to an approximately twofold reduction in TBLR1 expression level for WDR domain mutations with the most severe effects on MeCP2 and SETD5 binding (Supplementary Fig. 1d, e). Of note, patients heterozygous for deletion mutations that completely remove TBLR1, and therefore have a 50% dose of the protein, display relatively mild symptoms^4,5^, making it unlikely that the more severe pathology associated with some TBLR1 missense mutations is primarily driven by their effects on TBLR1 abundance^10,11,36^. Instead, the results suggest that for the majority of disease-causing TBLR1 mutations, disruption of their interaction with MeCP2, ANKRD11 and/or SETD5 contributes to the associated pathology.

The effects of TBLR1 mutations on binding to ANKRD11 and SETD5 raised the possibility that TBLR1 serves as a molecular bridge joining these two proteins, whilst also functioning as an adaptor protein to connect ANKRD11/SETD5 with the rest of the NCoR complex. To test this, we performed co-IP assays of mCherry-SETD5 and GFP-ANKRD11 that had been co-transfected into *TBLR1*-null cells or control cells expressing wild-type TBLR1. In the absence of TBLR1, the ability of ANKRD11 and SETD5 to interact with each other, and with the HDAC3 subunit of the NCoR complex, was strongly reduced (Fig. 1g). Residual binding observed in these experiments may be due to low levels of TBL1X in our TBLR1-null cells. We conclude that TBLR1 is necessary for robust interaction between ANKRD11, SETD5 and the rest of the NCoR complex.

### The complex between ANKRD11, SETD5 and NCoR resembles the yeast SET3 complex

The data presented point to the existence of a complex between SETD5, ANKRD11 and the other subunits of NCoR. It has previously been noted that SETD5 resembles the SET domain protein SET3 that is part of the yeast histone deacetylase-containing SET3 complex (SET3C)^40,44^. Further parallels exist between the subunits of SET3C and the components of the mammalian complex under study. Amino acid sequence conservation is limited, but most protein domains within subunits of the yeast complex have matching counterparts in mammals (Supplementary Fig. 2). These include an ankyrin repeat domain in HOS4 (similar to ANKRD11), a catalytically inactive SET domain in SET3 (similar to SETD5), a LisH domain and a WDR domain in SIF2 (similar to TBLR1), a SANT domain in SNT1 (similar to NCOR1) and a histone deacetylase domain in HOS2 (similar to HDAC3). Based on extensive domain conservation, we consider that the ANKRD11/SETD5/NCoR complex represents a mammalian ortholog of SET3C.

### Disease-causing missense mutations in ANKRD11 disturb SET3C assembly

**Fig. 2:**
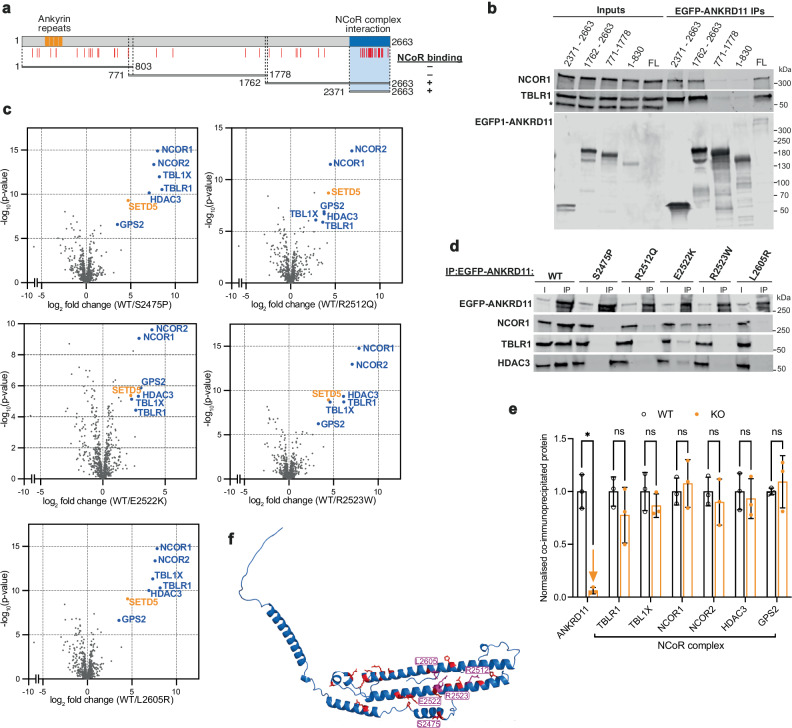
Disease-causing missense mutations in ANKRD11 disturb SET3C assembly. **a** A schematic of the functional domains in ANKRD11. The positions of pathogenic missense mutations from the ClinVar (likely pathogenic/pathogenic), DECIPHER (de novo, excluding benign mutations), and SFARI GENE (de novo) databases are indicated by vertical lines. Truncation constructs tested are annotated. **b** Western blot for TBLR1, NCOR1 and EGFP following immunoprecipitation of EGFP-ANKRD11 full length (FL) or truncation constructs from TBLR1^WT^ Flp-In™ T-REx™ 293 cells. * = non-specific band detected with TBLR1 antibody in input samples. **c** Volcano plots showing enrichment of protein interactions detected by mass spectrometry following immunoprecipitation of wild-type (WT) or indicated missense mutant EGFP-ANKRD11 expressed in HEK293T cells (*n* = 3 independent transfections per mutant). NCoR complex core components are indicated in blue. SETD5 is in orange. Statistical significance was calculated using two-tailed moderated *t*-tests. **d** Western blot for EGFP, NCOR1, TBLR1 and HDAC3 following immunoprecipitation of wild-type (WT) or indicated missense mutant EGFP-ANKRD11 expressed in TBLR1^WT^ Flp-In™ T-REx™ 293 cells. **e** Mass spectrometry quantification of SET3C components bound following immunoprecipitation of mCherry-SETD5 from mCherry-SETD5 transfected wild-type (WT) vs. ANKRD11 knock-out Flp-In™ T-REx™ 293 cells (KO) (*n* = 3 independent transfections per genotype). Mean and ±SD are indicated. Statistical significance was calculated using multiple two-tailed unpaired *t*-tests using the Holm–Šídák method to correct for multiple comparisons (adjusted *p* value) (ANKRD11 *p = 0.000567). **f** AlphaFold 3 predicted structure of a C-terminal globular domain in ANKRD11. Positions of pathogenic mutations are highlighted in red with those used in this study indicated in purple.

Our data demonstrate that many pathogenic mutations in TBLR1 disturb ANKRD11/SETD5 binding, indicating that disruption of SET3C may represent an underlying cause of the associated disorders. According to this hypothesis, mutations in SETD5 and ANKRD11 that affect SET3C assembly would be expected to cause disease. Considering first ANKRD11, the C-terminal domain contains a cluster of pathogenic KBGS mutations (Fig. 2a), and behaves as transcriptional repressor in reporter assays^54^. IP of transiently expressed GFP-tagged ANKRD11 truncations followed by western blot analysis revealed that a 292 amino acid C-terminal fragment of ANKRD11 is sufficient for binding to the NCoR complex, whereas other regions of ANKRD11 did not show detectable interaction with NCoR (Fig. 2b). Five pathogenic C-terminal mutant forms of ANKRD11 identified in patients with developmental delay and cognitive impairments^55^ (S2475P, R2512Q, E2522K, R2523W and L2605R) were transiently expressed in HEK293 cells as GFP-fusions, and IPs were analysed by mass spectrometry. The mutants all showed a substantial reduction in binding to both NCoR complex subunits and to SETD5 (Fig. 2c). Reduced NCoR binding by mutant ANKRD11 was confirmed by IP of GFP-ANKRD11 and western blotting with antibodies against NCoR complex components (Fig. 2d). Mass spectrometry of mCherry-SETD5 immunoprecipitations from wild-type as compared with ANKRD11 knock-out cells showed that loss of ANKRD11 from the SET3 complex does not affect the interactions between, or the stability of, the other SET3 complex components (Fig. 2e). Therefore, the predicted C-terminal globular domain of ANKRD11 binds to the TBLR1-containing NCoR complex (Fig. 2f), and mutations disrupting this interaction lead to KBGS. These findings are consistent with the idea that assembly into SET3C is essential for ANKRD11 function.

### An MeCP2-like motif in SETD5 mediates its incorporation into SET3C

**Fig. 3:**
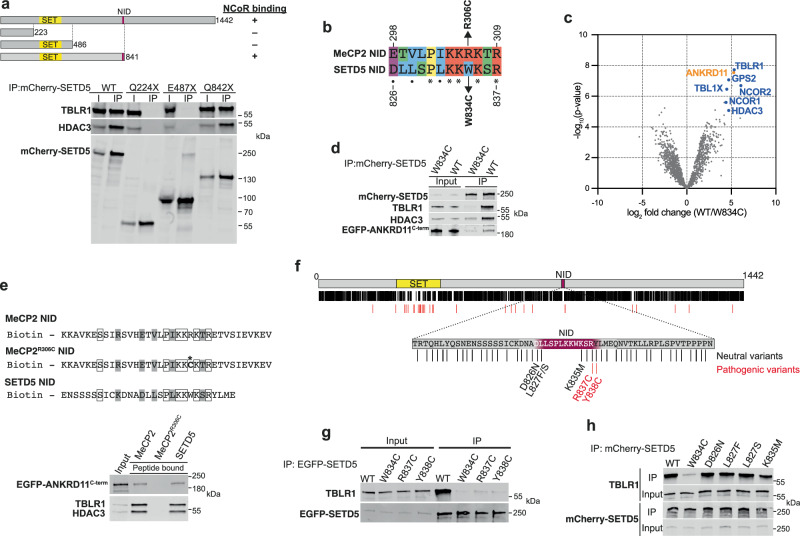
An MeCP2-like motif in SETD5 mediates its incorporation into SET3C, mutation of which causes disease. **a** Western blot for TBLR1, HDAC3 and mCherry following immunoprecipitation of wild-type (WT) mCherry-SETD5 or indicated truncation mutants from TBLR1^WT^ Flp-In™ T-REx™ 293 cells. NID NCoR interaction domain, SET SET domain. **b** Alignment of MeCP2 and SETD5 NIDs (NCoR interaction domains). Amino acids are coloured based on their properties (Clustal X). Asterisks = same amino acid, circles = similar amino acid. The R306C RTT-causing mutation in MeCP2 and the equivalent W834C mutation in SETD5 are indicated. **c** Volcano plots showing enrichment of protein interactions detected by mass spectrometry following immunoprecipitation of wild-type (WT) or W834C mutant EGFP-SETD5 expressed in TBLR1^WT^ Flp-In™ T-REx™ 293 cells (*n* = 3 independent transfections). Blue = NCoR complex core components. Statistical significance was calculated using two-tailed moderated *t*-tests. **d** Western blot for TBLR1, HDAC3, mCherry and EGFP following immunoprecipitation of wild-type (WT) or W834C mutant mCherry-SETD5 after co-transfection of TBLR1^WT^ Flp-In™ T-REx™ cells with mCherry-SETD5 and EGFP-ANKRD11^C-term^ (residues 1762-2663). **e** Western blot for EGFP, TBLR1 and HDAC3 after peptide pull-downs from EGFP-ANKRD11^C-term^ transfected TBLR1^WT^ Flp-In™ T-REx™-293 cell lysates. Sequences of the biotinylated peptides are shown with identical (white box) and similar (grey) amino acids indicated. * = R306C mutation. **f** A schematic of the functional domains in SETD5. Black vertical lines = neutral missense mutations from gnomAD 4.1 (excluding variants also in ClinVar). Red vertical lines = pathogenic missense mutations from the ClinVar (likely pathogenic/pathogenic), DECIPHER (de novo, excluding benign mutations), and SFARI GENE (de novo) databases. **g** Western blot for TBLR1 and EGFP following immunoprecipitation of wild-type (WT) or pathogenic mutant (R837C and Y838C) EGFP-SETD5 expressed in TBLR1^WT^ Flp-In™ T-REx™ 293 cells. The W834C mutation is used as a negative control. **h** Western blot for TBLR1 and mCherry following immunoprecipitation of wild-type (WT) and mutant mCherry-SETD5 expressed in TBLR1^WT^ Flp-In™ T-REx™ 293 cells. The mutations tested are from gnomAD v2.1.1, v3.1.2 and ExAC (excluding mutations also in ClinVar). The W834C mutation is used as a negative control.

To locate the region of SETD5 responsible for incorporation into SET3C, we tested a series of mCherry-tagged truncations that progressively remove C-terminal regions of SETD5 (Fig. 3a). Co-IP assays showed that 1–841 still bound to the NCoR complex, whereas the 1–486 and 1–223 truncations did not bind (Fig. 3a and Supplementary Fig. 3a). Examination of the SETD5 amino acid sequence between 486 and 841 revealed the motif PLKKWKS, which closely resembles the core TBLR1-binding NID of MeCP2 (PIKKRKT)^23,24^ (Fig. 3b). This offered a potential explanation for the similar effect of different TBLR1 mutations on binding to SETD5 and MeCP2 (Fig. 1e, f). To test whether the SETD5 NID-like motif can interact with TBLR1, we transiently expressed wild-type or mutated GFP-SETD5 in Flp-In T-REx cells expressing wild-type TBLR1. The W834C mutation was chosen, since a mutation at the corresponding position in MeCP2 (R306C) disrupts binding to TBLR1 and causes Rett syndrome without destabilising the protein (Fig. 3b)^23,24^. After IP of GFP-SETD5, mass spectrometry showed that the W834C mutation severely disrupts the association of SETD5 with components of SET3C, including ANKRD11 (Fig. 3c). The disruptive effect of the SETD5 W834C mutation on NCoR complex and ANKRD11 binding was confirmed by co-expressing mCherry-SETD5 with GFP-ANKRD11^C-term^, followed by anti-mCherry IP and western blotting with antibodies against TBLR1, HDAC3 and GFP (Fig. 3d). Pull-down assays using a biotin-tagged SETD5 NID peptide, and extracts from either wild-type mouse brain or cells transiently expressing transfected GFP-ANKRD11^C-term^, further demonstrated that this region of SETD5 is sufficient for SET3C incorporation (Fig. 3e and Supplementary Fig. 3b). Therefore SETD5 and MeCP2 each interact with TBLR1 via a closely related protein NID motif.

### Disease-causing missense mutations in SETD5 disturb SET3C assembly

Two pathogenic missense mutations in SETD5, R837C and Y838C, are located close to the NID identified above (Fig. 3f). To test the effect of these variants on TBLR1 binding, we transfected wild-type or mutant GFP-SETD5 into HEK293 cells and performed IPs followed by western blotting with antibodies against TBLR1. Both mutations drastically reduced the interaction of SETD5 with TBLR1 (Fig. 3g), suggesting that loss of this interaction might contribute to pathology. Consistent with the NID being a key functional motif in SETD5, this region of the protein is relatively depleted of neutral variants in individuals with no evidence of developmental delay (gnomAD) (Fig. 3f). Reinforcing this conclusion, neutral missense mutations at residues close to the NID (D826, L827 and K835; Fig. 3f) had no effect on SETD5 binding to the NCoR complex by the same assay (Fig. 3h). Together, these observations offer strong support for the idea that SETD5 function relies on the ability of its NID to interact with TBLR1, and that genetic disease associated with SETD5 mutations can involve loss of this interaction.

### SET3C is required for embryonic development

**Fig. 4:**
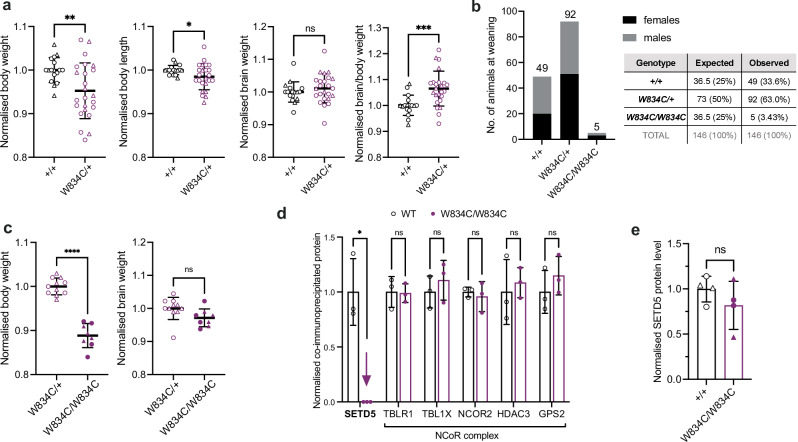
SET3C is required for embryonic development. **a** Body and brain weight, body lengths and brain/body weight ratio of heterozygous *Setd5*^*W834C/+*^ mice (*n* = 24; *n* = 16 males (circles), *n* = 8 females (triangles)) and their wild-type *Setd5*^*+/+*^ littermates (*n* = 17; *n* = 11 males (circles), *n* = 6 females (triangles)). Values were normalised to the average of the wild-type littermates of the same sex. Means and ±SD are indicated. Statistical significance was calculated using two-tailed unpaired *t*-test with Welch’s correction (body weight **p = 0.0028, body length *p = 0.0291, brain weight ^ns^*p* = 0.3475, brain/body weight ***p = 0.0004). **b** Numbers of *Setd5*^*W834C/W834C*^, *Setd5*^*W834C/+*^ and *Setd5*^*+/+*^ offspring at weaning from crosses between *Setd5*^*W834C/+*^ and *Setd5*^*W834C/+*^ mice on a mixed background (*n* = 2 backcrosses to C57BL/6J mice). Statistical significance was calculated using a two-tailed chi-squared test (****p = 1.24 × 10^−8^). **c** Body and brain weights of homozygous *Setd5*^*W834C/W834C*^ mice (*n* = 8, *n* = 4 males (circles), *n* = 4 females (triangles)) and heterozygous *Setd5*^*W834C/+*^ (*n* = 11; *n* = 5 males (circles), *n* = 6 females (triangles)) littermates. Values were normalised to the average of the *Setd5*^*W834C/+*^ littermates of the same sex. Means and ±SD are indicated. Statistical significance was calculated using a two-tailed unpaired *t*-test (body weight ****p = 7.5 × 10^−9^), brain weight ^ns^*p* = 0.1593). **d** Mass spectrometry quantification of NCoR complex components and SETD5 bound following immunoprecipitation with an antibody against NCOR1 from extracts from *Setd5*^*+/+*^ (WT) vs. *Setd5*^*W834C/W834C*^ homozygous male mouse brains (*n* = 3 per genotype). Means and ±SD are indicated. Statistical significance was calculated using multiple two-tailed unpaired *t*-tests using the Holm–Šídák method to correct for multiple comparisons (adjusted *p* value) (SETD5 *p = 0.004660). **e** Mass spectrometry quantification of SETD5 protein levels in *Setd5*^+/+^  vs. *Setd5*^*W834C/W834C*^ homozygous mouse brain extracts (*n* = 4 brains per genotype; *n* = 2 males (circles), *n* = 2 females (triangles)). Means and ±SD are indicated. Statistical significance was calculated using a two-tailed unpaired *t*-test (^ns^*p* = 0.2761).

To determine whether normal mouse development requires incorporation of SETD5 into the SET3 complex, we generated mice with the NID-disrupting W834C mutation in *Setd5* (Supplementary Fig. 4a). Heterozygous *Setd5*^*W834C/+*^ animals were viable and fertile but had somewhat reduced body weight—including kidney weight (Supplementary Fig. 4b)—and length compared to wild-type littermates (Fig. 4a). Brain weight was similar to controls for *Setd5*^*W834C/+*^ mice resulting in an increased brain to body weight ratio (Fig. 4a). These features closely match phenotypes previously described in mice heterozygous for a *Setd5*-null allele^38,56^. Homozygosity for this null mutation leads to embryonic lethality^40^. Similarly, when heterozygous *Setd5*^*W834C/+*^ animals (on a mixed background) were inter-crossed, only 3.4% of pups were homozygotes (*n* = 5/146), which is well below the expected Mendelian ratio of 25% (Fig. 4b). Therefore, failure of mutant W834C SETD5 to join the SET3C complex resembles the effects of a null mutation in the mouse *Setd5* gene, including, in the great majority of cases, pre-natal lethality. To better characterise the in vivo consequences of the W834C mutation at the molecular level, we performed RNA-seq on cortical tissue from 4-week-old *Setd5*^*W834C/+*^ and *Setd5*^*+/+*^ mice. Despite sequencing 6 biological replicates per genotype to a depth of 80 million reads, we observed very few significantly differentially regulated genes (Supplementary Fig. 4c). This observation raised the possibility that SETD5 might predominantly function in transcriptional regulation at earlier points in development.

The surviving homozygous *Setd5*^*W834C/W834C*^ mice had body weights that were more severely reduced relative to controls, whereas brain weight once again remained similar to littermates (Fig. 4c). Using brain nuclear extracts from these mice, we confirmed that the W834C knock-in mutation in endogenous *Setd5* prevents binding to NCOR1 without affecting SETD5 protein expression levels (Fig. 4d, e). Furthermore, co-immunoprecipitation experiments in *Setd5*^*W834C/W834C*^ mouse brain or mouse embryonic stem cells (ESCs), and mass spectrometry analysis of input protein extracts, showed that loss of SETD5 does not affect the integrity of the remaining SET3 complex (Fig. 4d and Supplementary Fig. 4d, e). As the rare *Setd5*^*W834C/W834C*^ homozygotes were fertile, we were able to ask whether they were exceptional survivors of a developmental bottleneck, or if they might have been rescued by a secondary genetic change. Compatible with the latter hypothesis, a cross between the homozygous *Setd5*^*W834C/W834C*^ and heterozygous *Setd5*^*W834C/+*^ mice produced a substantially higher proportion of homozygous pups (25.9% *n* = 45/174) (Supplementary Fig. 4f). To rule out the possibility of a secondary deleterious mutation that segregated with the *Setd5* W834C mutation, confounding its effects, we repeated the *Setd5*^*W834C/+*^ by *Setd5*^*W834C/+*^ cross with inbred mice (backcrossed >6 generations). No homozygous offspring (*n* = 0/31) (Supplementary Fig. 4g) were produced from these crosses, which aligns well with the data from the original cross (Fig. 4b). This also further supports the possibility that selection from genetic variation present in animals on a mixed background contributed to the reduced penetrance of the lethality in the *Setd5*^*W834C/W834C*^ by *Setd5*^*W834C/+*^ cross. Whilst identification of the precise cause of suppression will require further work, the mouse data as a whole demonstrates that a single amino acid change preventing SETD5 from assembling into SET3C has severe phenotypic consequences. SET3C assembly is therefore a critical molecular function of SETD5.

### SETD5 mutation does not rescue the RTT-like phenotypes of *Mecp2*-null mice

**Fig. 5:**
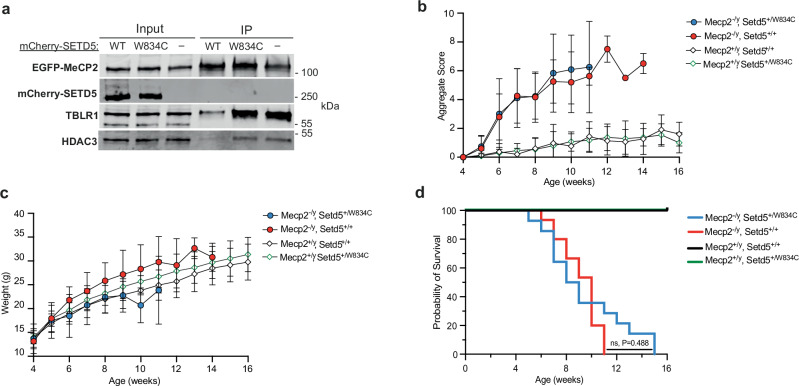
SETD5 mutation does not rescue RTT-like phenotype of *Mecp2-null* mice. **a** Western blot for TBLR1, HDAC3, mCherry and EGFP following immunoprecipitation of EGFP-MeCP2 from mixed EGFP-MeCP2 and mCherry-SETD5 (WT or W834C) transfected HEK293T cell extracts. Cell extracts were mixed at a ratio of 20:1 mCherry-SETD5 to EGFP-MeCP2. **b** Phenotypic scoring of male compound mutant *Mecp2*^*-/y*^, *Setd5*^*W834C/+*^ (*n* = 15) and littermate control animals (*n* = 14 *Mecp2*^*-/y*^, *Setd5*^*+/+*^ ; *n* = 15 *Mecp2*^*+/y*^, *Setd5*^*+/+*^; *n* = 12 *Mecp2*^*+/y*^, *Setd5*^*W834C/+*^). Data presented as mean values ± SD. **c** Body weights of animals shown in (**b**). Data presented as mean values ± SD. **d** Kaplan–Meier plot of survival of animals shown in (**b**). Statistical significance was calculated using a two-tailed log-rank (Mantel–Cox) test (^ns^*p* = 0.488).

An intriguing implication of our observation that MeCP2 and SETD5 interact with the same surface of TBLR1 is that the two proteins could compete for access to NCoR. To test this, we immunoprecipitated transfected EGFP-MECP2 from extracts expressing mCherry-SETD5^WT^ or, as a negative control, mCherry-SETD5^W834C^. Exogenous wild-type SETD5 reduced the amount of immunoprecipitated TBLR1, whereas mutant SETD5 had no effect (Fig. 5a). The results demonstrate that under these conditions, MeCP2 and SETD5 compete for binding to TBLR1. A potential consequence of competition is that MeCP2, a highly abundant chromatin protein, might sequester a major fraction of neuronal TBLR1, therefore limiting the amount of NCoR that is available for incorporation into SET3C. According to this scenario, the absence of MeCP2 due to loss-of-function mutations that cause RTT, may greatly increase the amount of NCoR that is available for interaction with SETD5. If so, some phenotypic features of RTT may arise due to unrestrained SET3C activity, that would normally be tempered due to the presence of MeCP2.

To investigate this hypothesis, we asked whether the RTT-like phenotypic consequences of MeCP2 disruption are relieved by reducing the interaction between SETD5 and TBLR1. *Mecp2-null* male mice, a model for RTT, appear normal at weaning, but from ∼5 weeks of age develop RTT-like symptoms that progress rapidly, reducing survival to a median age of 9 weeks^57^. We combined this mutation with heterozygosity for *Setd5*^*W834C*^, thereby reducing the amount of available functional SETD5 by approximately half. No change was observed in the trajectory of the RTT-like phenotypes of *Mecp2*^*-/y*^*,Setd5*^*W834C/+*^ mice compared to *Mecp*^*-/y*^*, Setd5*^*+/+*^ mice (Fig. 5b, c). Furthermore, there was no rescue of the median survival in *Mecp2*^*−/y*^*, Setd5*^*W834C/+*^ mice (Fig. 5d). These findings do not offer support for the notion that aspects of the RTT phenotype are due to rampant SET3C activity.

### The integrity of SET3C is required for transcriptional regulation

**Fig. 6:**
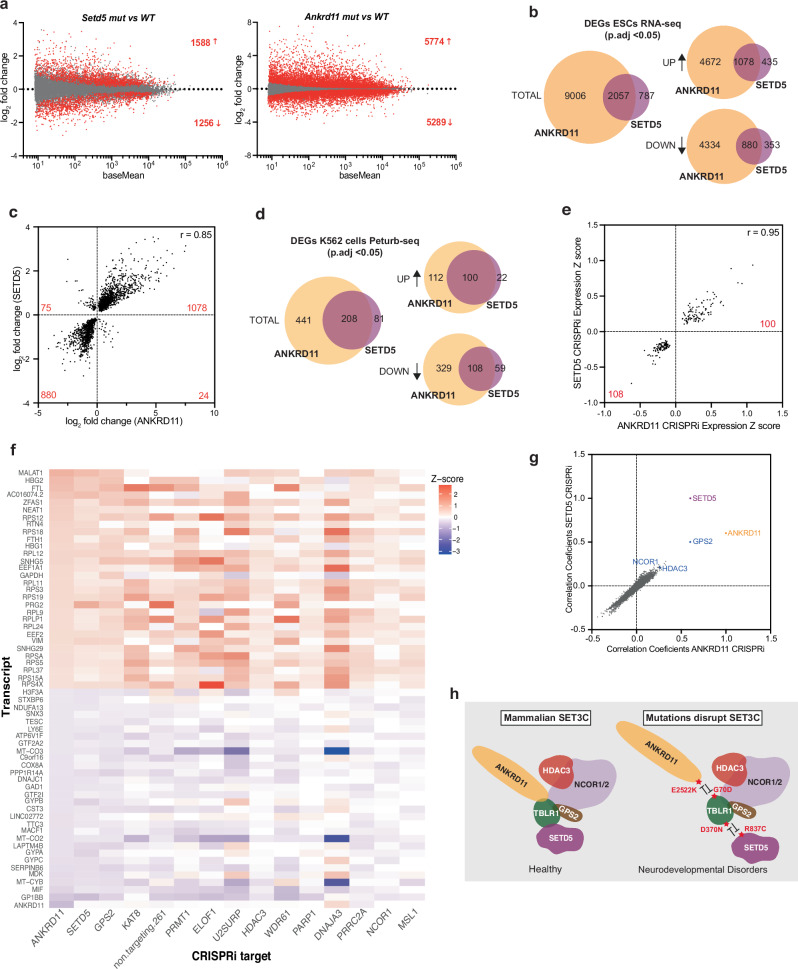
The integrity of SET3C is required for transcriptional regulation. **a** MA plots of gene expression changes in *Setd5* and *Ankrd11* mutant mouse embryonic stem cells (*n* = 3 independent clones for each genotype and *n* = 3 separate outgrowths per clone). Genes with significantly increased or decreased expression levels (*p* adj <0.05) are shown in red. Statistical significance was calculated using a two-tailed Wald test using Benjamini–Hochberg adjustment for multiple comparisons. **b** Venn diagrams showing the overlap of differentially expressed genes (DEGs) in *Setd5* and *Ankrd11* mutant ESCs. Separate diagrams are shown for total DEGs, up-regulated DEGs and down-regulated DEGs. **c** Scatterplot showing gene expression changes in *Ankrd11* and *Setd5* mutant mouse embryonic stem cells (Pearson *r* = 0.85). Points correspond to genes which are differentially expressed in both mutants (*p* adj <0.05). Statistical significance was calculated using a two-tailed Wald test using Benjamini–Hochberg adjustment for multiple comparisons. **d** Venn diagrams showing the overlap of differentially expressed genes (DEGs) following CRISPRi against SETD5 or ANKRD11 in K562 cells. Separate diagrams are shown for total DEGs, up-regulated DEGs and down-regulated DEGs. **e** Scatterplot showing differentially expressed genes in K562 cells following CRISPRi against ANKRD11 or SETD5 (Pearson *r* = 0.95). Points correspond to genes which are differentially expressed in both conditions (*p* adj <0.05). Statistical significance was calculated using the Anderson–Darling test using Benjamini–Hochberg adjustment for multiple comparisons. **f** Heatmap showing gene expression changes after CRISPRi against ANKRD11 and the twelve other proteins with the highest correlation coefficients with ANKRD11. The top 30 up- and down-regulated genes in ANKRD11 CRISPRi (ordered by Z-scores) are shown. **g** Scatterplot showing correlation coefficients of genome-scale CRISPRi scRNA-seq with CRISPRi against ANKRD11 and SETD5. **h** Schematic of the proposed model of the mammalian SET3 complex. Patient mutations in SET3C components disrupt the assembly of the complex and cause neurodevelopmental disorders.

The resemblance of mammalian SET3C to yeast SET3C suggests that its role may also be to modulate gene expression. To test this possibility, we performed RNA-seq on mouse embryonic stem cells (ESCs) homozygous for ANKRD11 or SETD5 missense mutations that disrupt their association with SET3C. A by-product of generating homozygous *Setd5* W834C ESCs was a clone homozygous for an in-frame deletion of amino acids K833 to K835 of SETD5. This ∆KWK mutation severely disrupted the ability of SETD5 to bind to TBLR1 but only moderately affected SETD5 protein levels (Supplementary Fig. 5a, b). We therefore analysed both W834C and ∆KWK mutant ESCs by RNA-seq (two independent W834C clones and one ∆KWK clone). *Setd5* mutant ESCs showed 2844 differentially expressed genes (DEGs) compared to the wild-type controls with similar numbers of genes up- and down-regulated (1588 up and 1256 down) (Fig. 6a). In the case of ANKRD11, we generated three independent clones of ESCs with the E2523K missense mutation, which is equivalent to the human KBG syndrome mutation E2522K. This mutation is present in the Yoda mouse line^58^ where homozygosity is associated with in utero lethality, and heterozygosity causes behavioural and neurological abnormalities^59^. *Ankrd11*^*yod/yod*^ ESCs had a greater number of DEGs, again with similar numbers of up and down regulated genes (11063 total DEGs; 5774 up and 5289 down) (Fig. 6a). The Yoda mutation, unlike the W834C mutation or ∆KWK in SETD5, substantially destabilises the ANKRD11 protein in both ESCs and in mouse brain (Supplementary Fig. 5c). This, or the presence of potentially redundant orthologous proteins, may explain its greater effect on transcription (see Discussion). Importantly, the transcriptional changes associated with these mutations are highly correlated (2057 total shared DEGs; 1078 up and 880 down) (Fig. 6b, c), supporting the view that ANKRD11 and SETD5 operate in the same pathway. This strong correlation is maintained when analysis is done using a more stringent *p* adjusted value or when only including the W834C samples or ∆KWK (Supplementary Fig. 5d–f). Shared DEGs include many involved in organogenesis, including differentiation of muscle, heart, bone and kidney (see top 25 GO terms in Supplementary Fig. 5g). These altered transcriptomes suggest that the mutant cells have a propensity to differentiate.

Since many DEGs shared by the mutant ES cell lines were related to differentiation, it seemed likely that these reflect destabilization of the stem cell state rather than primary effects of SET3C-deficiency on gene expression. In an attempt to reduce the downstream consequences of altered cell states and to test the effect of knocking down other SET3C subunits, we drew upon a Perturb-seq dataset derived from the human erythroid cell line K562 in which single-cell RNA-seq was used to assay the effects of genome-scale CRISPR interference-mediated perturbations^49^. The gene expression changes caused by depletion of ANKRD11 or SETD5 were once again highly correlated (Fig. 6d, e). Indeed, they were better correlated with each other, than with any of the other ~11,000 perturbations tested (Fig. 6f, g). Furthermore, depletions of the NCoR subunits NCoR1, GPS2 or HDAC3 also produced transcriptional changes that were well-correlated with those observed when ANKRD11 or SETD5 are knocked down (Fig. 6f, g). These data strongly suggest that ANKRD11, SETD5 and other components of SET3C function together to affect transcription. The most significantly dysregulated genes in Perturb-seq included ribosomal protein genes, haemoglobin, ferritin, vimentin, eosinophil major basic protein, and a set of abundantly expressed long non-coding RNAs (Fig. 6f), all of which were up-regulated. A common feature of these genes is that their mRNA transcripts are already abundant in unperturbed erythroid K562 cells. An intriguing possibility that requires future research is that SET3C preferentially restrains expression of highly transcribed genes.

## Discussion

Data from the 100k genome project places *ANKRD11*, *SETD5* and *MECP2* within the top ten most frequently mutated genes contributing to monogenic neurodevelopmental abnormalities^41^. Through analysis of patient mutations in transfection experiments, mutant cells in culture and mouse models, we pinpoint disruption of ANKRD11-TBLR1-SETD5 interactions as a common aetiology underlying these disorders (Fig. 5h). Previously reported SETD5 binding proteins include the NCoR complex^38,40^, the PAF complex^40^, ANKRD11^38^, G9a/GLP^60^ and BRD2^61^, and previously reported ANKRD11 interactions include p53^62^, HDAC3^39^, the p160 coactivator^39^, AIB1^63^, ADA3^64^, WDR5^65^, cohesin^66^ and the minor spliceosomal protein 65 K/RNPC3^67^ as well as self-association^68^. However, despite this information, it remained uncertain whether disruption of mechanisms involving these interactions leads to the pathology seen in SETD5 syndrome or in KBGS. Our data now suggest that brain function relies on the integrity of SET3C—the mammalian equivalent of the yeast SET3C complex. Strikingly, disruptions of different SET3C subunits can have similar effects on gene expression profiles. Thus, failure of SET3C-dependent transcriptional regulation is a shared feature of a set of distinct but related neurodevelopmental disorders. The embryonic lethality seen in our *Setd5*^*W834C/W834C*^ homozygous mice further supports the importance of SET3C integrity in vivo. Future studies of the more mildly affected *Setd5*^*W834C/+*^ heterozygous animals, incorporating comprehensive behavioural and neuroanatomical analyses, will be important to further define the in vivo consequences of SET3C disruption and to facilitate translational research.

Our findings help to explain why TBLR1 mutations are not associated with a single discrete clinical disorder. We find that TBLR1 has at least two clinically relevant functions (binding to MeCP2 and SET3C), and different mutations in TBLR1 disrupt these to different extents. For example, mutations in the TBLR1 N-terminal domain that prevent its association with ANKRD11 will disrupt the assembly of SET3C while preserving the ability of TBLR1 to serve as an adaptor for NCoR recruitment by MeCP2. Other mutations in the TBLR1 WDR domain interfere with both MeCP2 and SETD5 binding, and these might blend both RTT-like pathology and the pathology associated with SET3C dysfunction. There are additional reasons why mutations in TBLR1 might only rarely resemble RTT. TBLR1 is autosomal, whereas MeCP2 is an X-linked gene and therefore subject to X chromosome inactivation. Classical RTT is the result of a mosaic brain with complete loss of MeCP2-dependent functions in approximately half of the cells, the other half being functionally wildtype, whereas TBLR1 mutations would be associated with a molecular pathology that affects every cell. The relationship between different TBLR1 mutations and variable clinical outcomes might be further complicated by the effects of TBLR1 mutations on its other binding partners. One candidate is ANKRD12, a paralog of ANKRD11 for which loss-of-function mutations are associated with poor verbal-numerical reasoning^69^. Also, MLL5, a paralog of SETD5, may be functionally redundant with SETD5. Yet another binding partner is USP44—a ubiquitin protease that binds to the TBLR1 WDR domain^70^ and has been implicated in cognitive disability^71^. One of the implications of our observation that MeCP2 and SETD5 interact with the same surface of TBLR1 was explored in the present study. Since the two proteins compete for access to NCoR, mutations in one may enhance or disrupt the interaction with the other, leading to potential phenotypic consequences. However, when we reduced the amount of functional SETD5 by mutating its TBLR1 interaction domain, the phenotype of mice lacking MeCP2 was unaltered. This result does not rule out the possibility that an abundance of uncommitted NCoR due to the absence of MeCP2 contributes to the RTT phenotype, but it offers no support for the hypothesis that hyperactivity of SET3C is responsible. It remains possible that reducing the reduction in SETD5 by ~2-fold is insufficient to reveal phenotypic effects. Alternatively, SETD5 may not be highly expressed in mature neurons where MeCP2 is most abundant.

Our findings indicate that SET3C may limit the transcription of highly expressed genes. This is consistent with a recent report that in *Drosophila* embryos, HDAC3 depletion up-regulated genes which were already highly expressed in control tissues^72^. Work in yeast suggests that the SET3 complex transcriptionally suppresses sporulation genes^44^ and also spurious transcriptional initiation from cryptic promoters^45,73^. The yeast SET3 complex is reportedly targeted to the dimethylated form of lysine 4 of histone H3 via the PHD finger of its Set3 subunit^46^. However, although SETD5 is related to Set3, it does not contain a comparable PHD finger (unlike its mammalian paralog MLL5). *Drosophila* UpSET—an ortholog of SETD5 but also possessing a PHD finger—has been implicated in preventing the ectopic spread of active chromatin away from genes and into neighbouring flanking regions^74^. ChIP-seq from different groups suggests that SETD5 binds preferentially to transcription start sites^38^, or to active gene bodies^56^. Understanding the SET3C function will require reconciliation of these observations, in particular by elucidating the molecular determinants that target SET3C to chromatin. Several interactions and domains could plausibly play a role in specifying genomic binding, including the PAF complex, the ankyrin repeat domain of ANKRD11, the SET domain of SETD5 (which contains several pathological missense mutations), and other subunits of SET3C. Recruitment via the ANKRD11 ankyrin repeats is an attractive hypothesis since they share significant homology with the ankyrin repeats of BARD1—a domain that binds to the N-terminal tail of histone H4^75^. At present, however, the mechanism by which SET3C is recruited to chromatin remains unknown.

The finding that mutations affecting these three genes impact a shared regulatory pathway involved in related neurological disorders is relevant to the quest for therapies. Most immediately, lessons learned about the treatment of patients with one SET3C-related disorder might help to guide the management of patients with other related conditions. Looking further ahead, efforts to develop gene therapy as a treatment strategy for SETD5 syndrome or KBGS may be complicated by the large size of SETD5 and ANKRD11, which exceeds the limited capacity of available viral vectors. One possible solution to this problem is genome editing, which is relatively indifferent to gene size—a technology that is rapidly developing. Another is to develop truncated versions of SETD5 and ANKRD11, which nevertheless retain all their critical domains and motifs, and so can function normally. The design of such constructs will require a complete inventory of the functional elements in SETD5 and ANKRD11. Just as reporting the key MeCP2-TBLR1 interaction^23,24^ paved the way for developing the miniaturised version of MeCP2^35^ that underlies the first RTT gene therapy clinical trial^76^, our findings on SET3C-related disorders may also hold translational potential.

## Methods

### Animals

Procedures were carried out by certified persons, licensed by the UK Home Office and according to the Animals (Scientific Procedures) Act 1986 under project licences (PPLs) 60/4547 and PP4326006. Ethical review of the PPLs was carried out by the University of Edinburgh Animal Welfare and Ethical Review Board (AWERB). Mice (*Mus Musculus*) were bred in a specific-pathogen-free facility in individually ventilated cages, with wood chippings, tissue bedding and environmental enrichment and were given *ad libitum* access to food and water. Rooms were maintained on a 12-h light/dark cycle at 45–65% humidity at 20–24 °C.

The *Setd5*^*W834C*^ mice were generated by injection of heterozygous *Setd5*^*W834C/+*^ ESCs (129/Ola E14TG2a) (see below) into E3.5 blastocysts obtained from C57BL/6J (Charles River RRID:IMSR_JAX: 000664) females after natural matings. Blastocysts were transferred to pseudo-pregnant recipient females, and chimeric offspring were mated to C57BL/6 J mice to establish the line. The *Ankrd11*^*Yod*^ mice (C3H.Cg-Ankrd11^Yod^/H) were rederived from mouse sperm purchased from the EMMA mouse repository (EM:00380). Mouse genotyping was initially performed by PCR (see Supplementary Data 1 for primers) and XcmI digest for *Setd5*^*W834C*^ mice, then by TransnetYX. *Ankrd11*^*Yod*^ and Setd5^W834C^ mice were genotyped by TransnetYX (TransnetYX assays available upon request). Weight and length measurements and tissues for molecular analysis were taken from 6- week-old mice (on a mixed background). Cortical brain tissue for RNA-seq analysis was taken from 4-week-old mice, which were backcrossed to C57BL/6 J mice for *n* = 5 generations. Roughly equal numbers for each sex of mice were used for phenotypic analysis of *Setd5*^*W834C*^ mice, mass spectrometry quantification of protein levels in brain extracts and RNA-seq analysis of cortical tissue. Only male mice were used for immunoprecipitation followed by mass spectrometry from brain extracts to analyse protein-protein interactions, as these findings should not be influenced by sex.

Cohorts of compound mutant *Mecp2*^*−/y*^*,Setd5*^*W834C*^ mice and littermate controls (on a mixed background) with at least 12–15 animals per genotype were weighed and scored weekly from 4 to 16 weeks for a range of RTT-like phenotypes (mobility, gait, hindlimb clasping, tremor, breathing and general condition) to give an aggregate score between 0 and 12 as previously described^77,78^. Scoring was terminated at 16 weeks because all *Mecp2-null* animals had died by that timepoint. Animals receiving a maximum score of 2 for tremor, breathing or general condition or which had lost 20% of their body weight, had reached the severity limit of the experiment according to the Home Office license and were humanely culled. These animals were counted as having ‘died’ for the purposes of survival data. Animals of any genotype which were culled for reasons not linked to the mutation, such as fighting with cage mates, were removed from survival plots at that point (censored data). Male mice only were used for phenotypic analysis of *Mecp2*^*-/y*^*,Setd5*^*W834C/+*^ mice, as the *Mecp2* gene is X-linked and hemizygous male mice have a much more severe phenotype than female mice.

### Cell culture

Mouse embryonic stem cells (ESCs) (129/Ola E14TG2a) were cultured on gelatinised culture dishes and flasks in Glasgow MEM medium (GMEM) (Gibco 21710025) supplemented with recombinant mouse Leukaemia Inhibitory Factor (LIF) (ESGRO), 10% FBS (Gibco), 1% Non-essential amino acids (Gibco), 1% Sodium pyruvate (Gibco) and 0.1% β-mercaptoethanol (Gibco) and transfected with Lipofectamine 2000 (Thermo). HEK293 cells (ATCC, CRL-1573) were grown in DMEM (Gibco, 41966029) supplemented with 10% FBS (Gibco) and transfected with Lipofectamine 2000 (Thermo). Flp-In™ T-REx™ 293 cells (Thermo, R78007) were cultured according to the manufacturer’s protocol. Mouse ESCs were authenticated by their ability to contribute to the germline in chimeras derived from blastocyst injection. No authentication was performed on the human cell lines used. HEK293 of Flp-In^TM^ T-REx^TM^ 293 cells were harvested 24 h after transfection and/or induction of protein expression and then stored at −80 °C for future analysis.

*Setd5*^*W834C/+*^ heterozygous ESCs were generated using two CRISPR-Cas9 guide RNAs (Supplementary Data 1) targeting either side of exon 17 and a long single-stranded DNA (lssDNA) template with the W834C mutation and silent PAM-abolishing mutations (Supplementary Data 1). The ssDNA template was excised from a plasmid using the nicking endonucleases Nt.BspQI and Nb.BsmI, and purified using the LssODN Preparation Kit (Biodynamics Laboratory Inc., Tokyo, Japan). The two exonic CRISPR-Cas9 guide RNA off-target sites with CFD scores >0.02 (according to CRISPR.org web tool) were PCR amplified and sequenced, and no mutations were detected (Source Data). *Setd5*^*W834C/W834C*^ homozygous ESCs were generated using a single CRISPR-Cas9 guide RNA (Supplementary Data 1) and a 200 bp DNA oligonucleotide (IDT) containing the W834C mutation (Supplementary Data 1). Targeted clones were identified and verified by XcmI restriction digest (the W834C mutation destroyed a XcmI site) and by sequencing (analysed using SNAPgene v4.3.11).

*ANKRD11*^*E2523K/E2523K*^ homozygous ESCs were made using a single CRISPR-Cas9 guide RNA (Supplementary Data 1) and a 200 bp DNA oligonucleotide (IDT) containing the E2523K mutation and silent mutations within the seed region of the gRNA target DNA sequence (Supplementary Data 1). Targeted clones were identified and verified by MseI restriction digest (inserted mutations introduce an MseI site) and by sequencing.

TBLR1 was knocked out in Flp-In™ T-REx™ 293 cells using a CRISPR-Cas9 guide RNA (Supplementary Data 1) targeting a region just downstream of the ATG initiation codon. Subsequent sequencing identified clones which were compound heterozygous for frame shift mutations in this region. ANKRD11 was knocked out in Flp-In™ T-REx™ 293 cells using CRISPR-Cas9 guide RNA (Supplementary Data 1) targeting a region at the beginning of exon 9. Subsequent sequencing identified clones which were compound heterozygous for frame shift mutations (1 bp deletion and 25 bp deletion), and mass spectrometry on cell extracts confirmed ANKRD11 was absent.

Tetracycline-inducible expression constructs, derived from pcDNA5/FRT/TO (Supplementary Data 1), were inserted into the FRT site of Flp-In™ T-REx™ 293 cells according to the manufacturer’s instructions. Clones were verified by sequencing, and single integration of the plasmid into the FRT site was confirmed by PCR and Southern blotting (see Supplementary Data 1 for primers).

### Plasmids

The coding sequences of ANKRD11, SETD5, TBLR1 and/or MeCP2 were cloned into pEGFP/mCherry-C1, pmCherry-N1, p3xFLAG-CMV10, pPyCAG-EGFP and/or pcDNA5/FRT/TO (Thermo, V652020). The NanoLuc LgBiT or SmBiT fragments were added to the C-terminus of coding sequences (see Supplementary Data 1 for plasmid sequences). Truncation and missense mutation variants of these plasmids were produced by PCR and Gibson assembly or by site-directed mutagenesis (see Supplementary Data 1 for primer sequences).

### Western blotting

Samples for western blots were either immunoprecipitation samples, peptide pull-down samples, input samples or whole-cell protein extracts. For whole-cell protein extracts: cell pellets were lysed in NE10 buffer (20 mM HEPES pH 7.9, 10 mM KCl, 1 mM MgCl_2_, 0.1% Triton X-100, 20% glycerol, 0.5 mM DTT, Benzonase 1000U/ml and protease inhibitors cocktail) and incubated at room temperature for 10 min. 2x SDS-PAGE sample buffer was added, and the solution was then vortexed, boiled for 3 min, snap frozen on dry ice, and then boiled for a further 3 min. Antibodies used for western blotting were as follows: HDAC3 (Abnova, H00008841-M02, lot 15281, clone 3E11), HDAC3 (Abcam, ab32369, lot 1002834-27), mCherry (Abcam, ab167453, lot 61100), mCherry (Abcam, ab125096, lot GR309694-1, clone 1C51), NCOR1 (Cell Signalling Technology, 5948S, lot 2), GFP (Takara, 632592, lot 2310030), GFP (Takara, 632381, lot A8034133, clone JL-8), SIN3A (Abcam, ab3479, lot 1070214), TBLR1 (Santa Cruz Biotechnology, sc-100908, lot J0115, clone L-08), γ-tubulin (Sigma, T5326, lot 0000299270) and Histone H3 (Abcam, ab1791, lot GR3421644-1). Antibody dilutions used were 1:1000 for all, apart from H3, which was 1:10,000. Western blots were imaged on the LI-COR® Odyssey CLx system and quantified using the Image Studio Lite software v5.2.5 (LI-COR Biosciences).

### NanoLuc assays

NanoLuc complementation assays were performed as described before^52^ except that full-length TBLR1 was used rather than the isolated WDR domain. Briefly, expression vectors for TBLR1 fused to LgBiT and either MeCP2, SETD5 or ANKRD11 fused to SmBiT were co-transfected into HEK293 cells. After 24 h, extracts were prepared and diluted in passive lysis buffer (Promega, E1910) before the addition of furimazine (N1110) and measuring luminescence.

### Mass spectrometry

Samples were prepared either by in gel digestion as described elsewhere^79^ or (for the analysis of pEGFP-SETD5 IPs) by filter-aided sample preparation as described elsewhere^80^. Immunoprecipitations were done in triplicate from three separate cell transfections per construct or brains from three separate animals per genotype.

LC-MS analyses were performed on Orbitrap Fusion™ Lumos™ Tribrid™ Mass Spectrometer (Thermo Fisher Scientific, UK) using the Data Dependent Acquisition (DDA) mode and on Orbitrap Exploris 480™ on a Data Independent Acquisition (DIA) mode, both coupled online, to an Ultimate 3000 HPLC (Dionex, Thermo Fisher Scientific, UK). Peptides were separated on a 50 cm (2 µm particle size) EASY-Spray column (Thermo Scientific, UK), which was assembled on an EASY-Spray source (Thermo Scientific, UK) and operated constantly at 50 °C. Mobile phase A consisted of 0.1% formic acid in LC-MS grade water, and mobile phase B consisted of 80% acetonitrile and 0.1% formic acid. Peptides were loaded onto the column at a flow rate of 0.3 μl min^−1^ and eluted at a flow rate of 0.25 μl min^−1^ according to the following gradient: 2 to 40% mobile phase B in 150 min and then to 95% in 11 min. Mobile phase B was retained at 95% for 5 min and returned back to 2% a minute after until the end of the run (190 min).

For DDA, survey scans were recorded at 120,000 resolution (scan range 350–1500 m/z) with an ion target of 4.0e5, and an injection time of 50 ms. MS2 was performed in the ion trap at a rapid scan mode, with an ion target of 2.0e4 and HCD fragmentation^81^ with a normalised collision energy of 27. The isolation window in the quadrupole was 1.4 Thomson. Only ions with a charge between 2 and 7 were selected for MS2. Dynamic exclusion was set at 60 s.

For DIA, MS1 scans were recorded at 120,000 resolution (scan range 350–1650 m/z) with an ion target of 5.0e6, and an injection time of 20 ms. MS2 was performed in the Orbitrap at 30,000 resolution with a scan range of 200–2000 m/z, maximum injection time of 55 ms and AGC target of 3.0E6 ions. We used HCD fragmentation with stepped collision energy of 25.5, 27 and 30. We used variable isolation windows throughout the scan range, ranging from 10.5 to 50.5 m/z. Narrow isolation windows (10.5–18.5 m/z) were applied from 400 to 800 m/z and then gradually increased to 50.5 m/z until the end of the scan range. The default charge state was set to 3. Data for both survey and MS/MS scans were acquired in profile mode.

MaxQuant^82^ version 1.6.1.0 was used to process the raw files, and search was conducted against the *Mus musculus* protein database (released in July 2017), using the Andromeda search engine^83^. For the first search, peptide tolerance was set to 20 ppm, while for the main search, peptide tolerance was set to 4.5 ppm. Isotope mass tolerance was 2 ppm and the maximum charge was 7. Digestion mode was set to specific with trypsin allowing maximum of two missed cleavages. Carbamidomethylation of cysteine was set as a fixed modification and oxidation of methionine was set as variable modifications. Label-free quantitation analysis was performed using the MaxLFQ algorithm as described^84^. Absolute protein quantification was performed as described^85^. Peptide and protein identifications were filtered to 1% FDR.

The DIA-NN software platform^86^ version 1.8.1 was used to process the DIA raw files, and a search was conducted against the *Mus musculus* complete/reference proteome (Uniprot, released in July, 2017). Precursor ion generation was based on the chosen protein database (automatically generated spectral library) with deep-learning based spectra, retention time and IMs prediction. Digestion mode was set to specific with trypsin allowing maximum of two missed cleavages. Carbamidomethylation of cysteine was set as a fixed modification. Oxidation of methionine, and acetylation of the N-terminus were set as variable modifications. The parameters for peptide length range, precursor charge range, precursor m/z range and fragment ion m/z range, as well as other software parameters, were used with their default values. The precursor FDR was set to 1%. Downstream data analysis was performed using the DEPP package v1.26.0 in R v4.4.0.

### Binding assays

Brains were subject to Dounce homogenisation in ice-cold NE10 buffer (20 mM HEPES NaOH, pH 7.5, 10 mM NaCl, 1 mM MgCl_2_, 0.1% Triton X-100, 10 mM 2-mercaptoethanol and protease inhibitors) followed by 5 min centrifugation at 500×*g*. Insoluble material was resuspended in NE10 and treated for 5 min at 25 °C with 250 units of benzonase (Merck, E1014). 5 M NaCl was then added to give a final NaCl concentration of 150 mM. After 20 min mixing and 30 min 16,000×*g* centrifugation, all at 4 °C, the supernatant was taken as starting material for immunoprecipitation or peptide pull-down assays. Cell extracts were prepared by Dounce homogenisation in NE10 before benzonase treatment (as above), adjusting the NaCl concentration to 150 mM, and 30 min centrifugation at 16,000×*g* at 4 °C. Supernatants were then taken as starting material for immunoprecipitations.

For immunoprecipitation (IP) of GFP-SETD5 for mass spectrometry, anti-GFP (Merck, 11814460001) was used. For the IP of NCOR1 from the mouse brain anti-NCOR1 (Cell Signalling Technology, 5948S) was used. GFP trap (Chromotek, GTA-20) and RFP trap (Chromotek, RTA-20) were used for all other IPs. IPs with GFP/RFP trap were performed as described previously^23^. Briefly, extracts were mixed with GFP/RFP trap beads for 30–60 min at 4 degrees before washing four times with NE10 buffer containing 150 mM NaCl. IPs with anti-GFP and anti-NCOR1 were performed similarly except that 5 µg of antibody was first bound to protein G Dynabeads (Thermo, 10003D) by cross-linking by mixing for 30 min at 20 °C in borate buffer (40 mM boric acid, 40 mM sodium tetraborate decahydrate) with 20 mM dimethyl pimelimidate. Bound complexes were eluted by boiling with SDS-PAGE sample buffer, except for the IP of GFP-SETD5 for mass spectrometry, which was eluted by mixing for 15 min at 60 °C in 50 µl 0.1% Rapigest in 50 mM Tris-HCl pH 8.0. For peptide pull-down assays, 50 µg of peptide with an N-terminal biotin tag (see Supplementary Data 1 for peptide sequences) was bound to 10 µl streptavidin sepharose (Merck, GE17-5113-01). As with the IP assays, complexes were then precipitated from extracts, washed, and eluted with SDS-PAGE sample buffer.

### RNA-seq

Total RNA was isolated from *Setd5*^*W834C/W834C*^, *Setd5*^*∆KWK/∆KWK*^*, Setd5*^*+/+*^*, Ankrd11*^*yod/yod*^ and *Ankrd11*^*+/+*^ ESC clones (*n* = 3 separate clones (*Setd5*^*+/+*^, *Ankrd11*^*yod/yod*^ and *Ankrd11*^*+/+*^), *n* = 2 seperate clones (*Setd5*^*W834C/W834C*^), n = 1 clone (*Setd5*^*∆KWK/∆KWK*^) and *n* = 3 separate outgrowths per clone) or *Setd5*^*W834C/+*^ and *Setd5*^*+/+*^ cortical brain tissue (*n* = 6 animals per genotype, *n* = 3 females, *n* = 3 males) using TRIzol (Thermo, 15596026) followed by purification with the Direct-zol RNA Miniprep Plus kit (Zymo Research, R2070) according to manufacturer’s protocol. An extra genomic DNA contamination removal step was performed for ESC samples with the Invitrogen™ DNA-*free*™ DNA Removal Kit (AM1906), and the remaining DNA-free RNA was tested for purity using PCR for genomic loci. Total RNA was tested on the 2100 Bioanalyzer (Agilent Technologies) to ensure high quality and quantified using a Qubit (Invitrogen). *Drosophila* S2 cells (5% total cell count (ESCs) and 6000 cells per 1 mg tissue (cortex)) were added to each sample as a spike-in before proceeding with RNA extraction. ERCC RNA Spike-in control mix (Ambion) was added to RNA samples before library preparation and sequencing. Strand-specific library preparation with rRNA-depletion was performed by Novogene, followed by whole-transcriptome sequencing using HiSeq X (Novogene Europe, UK). Differential gene expression analysis was performed using DESeq2 v1.42.0 as described in (https://github.com/kashyapchhatbar/SET3C-manuscript-2026)^87^ (also see Data availability section). Gene Ontology (GO) analysis was performed using clusterProfiler v4.8.1.

### Statistics and reproducibility

Values are expressed as mean ± standard deviation (SD) as indicated. For quantitative experiments, the number and type of replicates are stated in the Figure Legends. Statistical analysis was carried out in GraphPad Prism v10.6.1, DEP package v1.26.0 in R v4.4.0 and DESeq2 v1.42.0. Information on the statistical tests used can be found in the figure legends. For immunoprecipitation followed by mass spectrometry analysis, statistical significance was calculated using two-tailed moderated *t*-tests. Statistical analysis of mass spectrometry data for the quantification of protein levels in extracts was performed using multiple two-tailed unpaired *t*-tests using the Holm–Šídák method to correct for multiple comparisons (adjusted *p* value) (NCoR complex components) or a two-tailed unpaired *t*-test (for SETD5 or ANKRD11 levels in wild-type or mutant extracts). Statistical analysis of mouse weights was performed using a two-tailed unpaired *t*-test with or without Welch’s correction. Statistical analysis of survival to weaning of different mouse genotypes was done using a two-tailed chi-squared test (for three possible genotypes) or a two-tailed binomial test (for two possible genotypes). Statistical significance of *Mecp2*-mutant mouse survival was calculated using a two-tailed Log-rank (Mantel–Cox) test. For differential gene expression analysis, statistical significance was calculated using a two-tailed Wald test using Benjamini–Hochberg adjustment for multiple comparisons. The statistical test used for Gene Ontology analysis was the one-tailed hypergeometric test using Benjamini–Hochberg adjustment for multiple comparisons. For Peturb-seq data, statistical significance was calculated using the Anderson–Darling test using Benjamini–Hochberg adjustment for multiple comparisons. Linear regression analysis of differentially expressed genes in different conditions was determined using Pearson correlation coefficients, with coefficients displayed in Figures and Figure legends. Immunoprecipitation followed by western blot experiments (Figs. 1g, 2b, 3e, g, h, and 5a and Supplementary Figs. 1c and 4c) were repeated independently (*n* = 2) with similar results (see Source data). No statistical methods were used to predetermine sample sizes. No data was excluded from the analyses except for the mouse survival experiment (Fig. 5d) where animals of any genotype which were culled for reasons not linked to the mutation, such as fighting with cage mates, were removed from survival plots at that point (censored data). Randomisation was not applicable to the experiments in this study. Investigators were blinded to the genotype of animals in our phenotypic scoring experiments, but not for other assays.

### Reporting summary

Further information on research design is available in the Nature Portfolio Reporting Summary linked to this article.

## Supplementary information

Supplementary Information

Description of Additional Supplementary Files

Supplementary Data 1

Reporting Summary

Transparent Peer Review file

## Source data

Source Data

## Data Availability

Raw data for Figures and associated Supplementary Figs. are provided in the Source data Zip file, including uncropped western blot images, formatted and uncropped western blots for replicate experiments and datasheets containing individual values underlying each plot. The mass spectrometry data generated in this study have been deposited in the PRIDE partner repository under the accession codes PXD063846 and PXD077916. The RNA-sequencing data generated in this study have been deposited in the ArrayExpress database under the accession codes E-MTAB-15318 (ES cells) and E-MTAB-16803 (cortex). The Perturb-seq data used in this study are available in Figshare Plus under 10.25452/figshare.plus.21632564.v1 (*p* values) and 10.25452/figshare.plus.20029387 (Z-scores). Source data are provided with this paper.

## Code availability

Code for RNA-seq and differential gene expression analysis is available at https://github.com/kashyapchhatbar/SET3C-manuscript-2026. The code is archived in Zenodo and is accessible via 10.5281/zenodo.19736279^87^.

## Materials availability

All plasmids, cell lines and animal lines generated as part of this study are available by request to the corresponding authors.
