# Supplementary material for: Dysfunction of a SET3-like complex underlies a family of related neurological disorders
Source: Nat Commun. 2026 May 16;17:6729. doi: 10.1038/s41467-026-73227-5 (PMC13385800; doi:10.1038/s41467-026-73227-5)

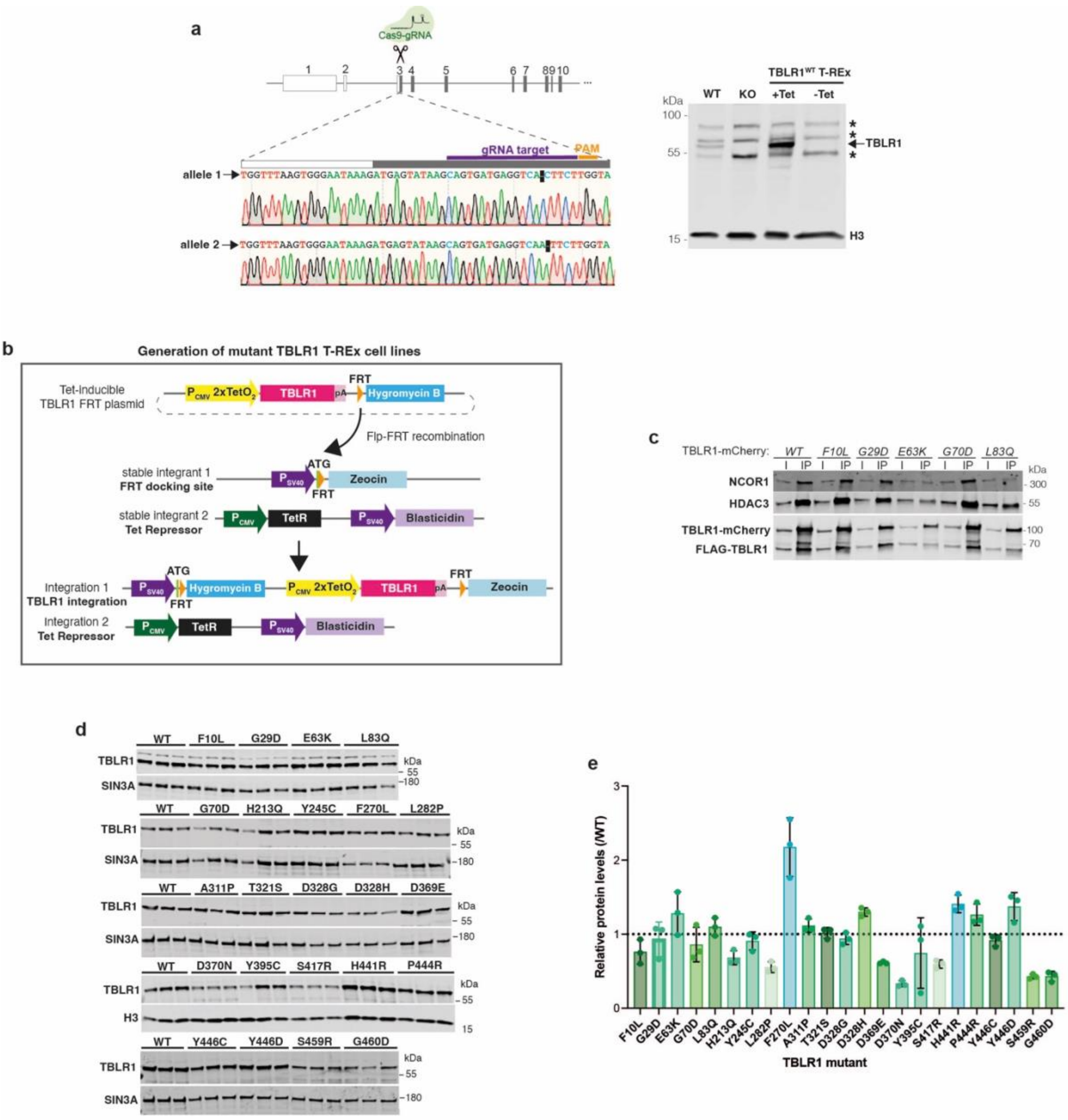

Supplementary Figure 1: Pathogenic mutations in TBLR1 do not substantially affect protein stability.

a, Left: Targeting strategy used to knock-out *TBL1XR1* in Flp-In™ T-REx™-293 cells and the sequences of the two targeted alleles with 1 bp deletions. Right: Western blot for TBLR1 and histone H3 using extracts from untargeted wild-type cells (WT), TBLR1 knock-out cells (KO) and KO cells with a TBLR1^{WT} cDNA under a tetracycline inducible promoter (TBLR1^{WT} T-REx) inserted into the FRT site, treated with (+) or without (–) tetracycline. *Non-specific binding of TBLR1 antibody. H3 serves as a loading control. **b,** A schematic of Flp-recombinase mediated transgene insertion into the FRT site of Flp-In™ T-REx™-293 cells. **c,** Western blot for NCoR1, HDAC3, mCherry and FLAG following immunoprecipitation of wild-type (WT) or indicated mutated forms of TBLR1-mCherry from TBLR1^{WT} Flp-In™ T-REx™ 293 cells that were co-transfected with TBLR1-mCherry and FLAG-TBLR1. **d,** Western blot for TBLR1 and SIN3A/H3 using extracts from tetracycline-induced wild-type (WT) and indicated mutant TBLR1 Flp-In™ T-REx™ 293 cell lines (n = 3 independent tetracycline inductions for each mutation). SIN3A or Histone H3 serves as loading controls. **e,** Quantification of Western blots showing TBLR1 protein level relative to WT in the indicated mutant TBLR1 mutant Flp-In™ T-REx™-293 cell lines. Mean and +/- SD are indicated.

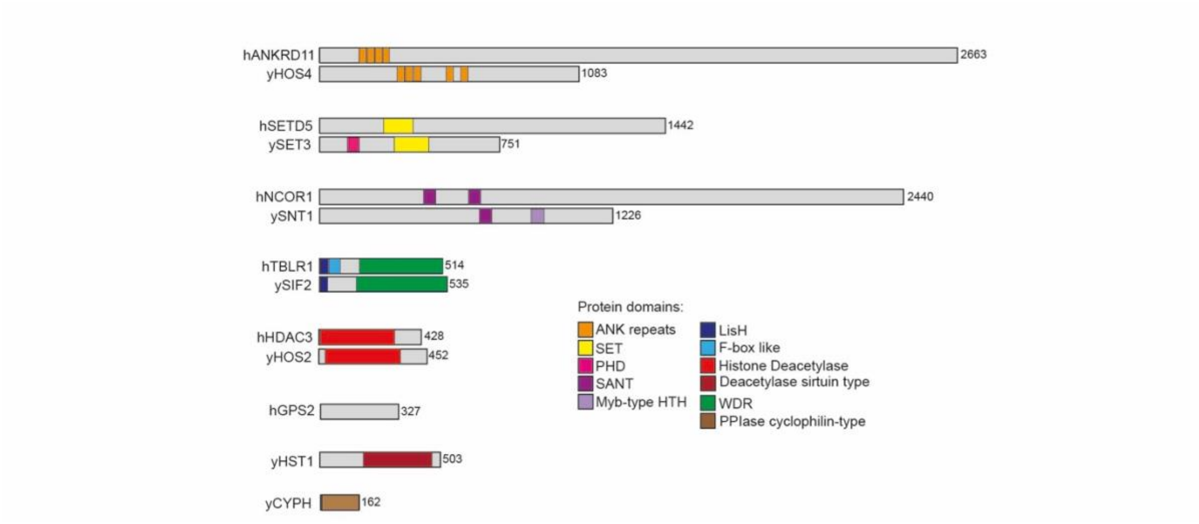

Supplementary Figure 2: The complex between ANKRD11, SETD5 and NCoR resembles the yeast SET3 complex.

A schematic showing domain structures of yeast (y) SET3 complex components and the analogous proteins in the human (h) assembly.

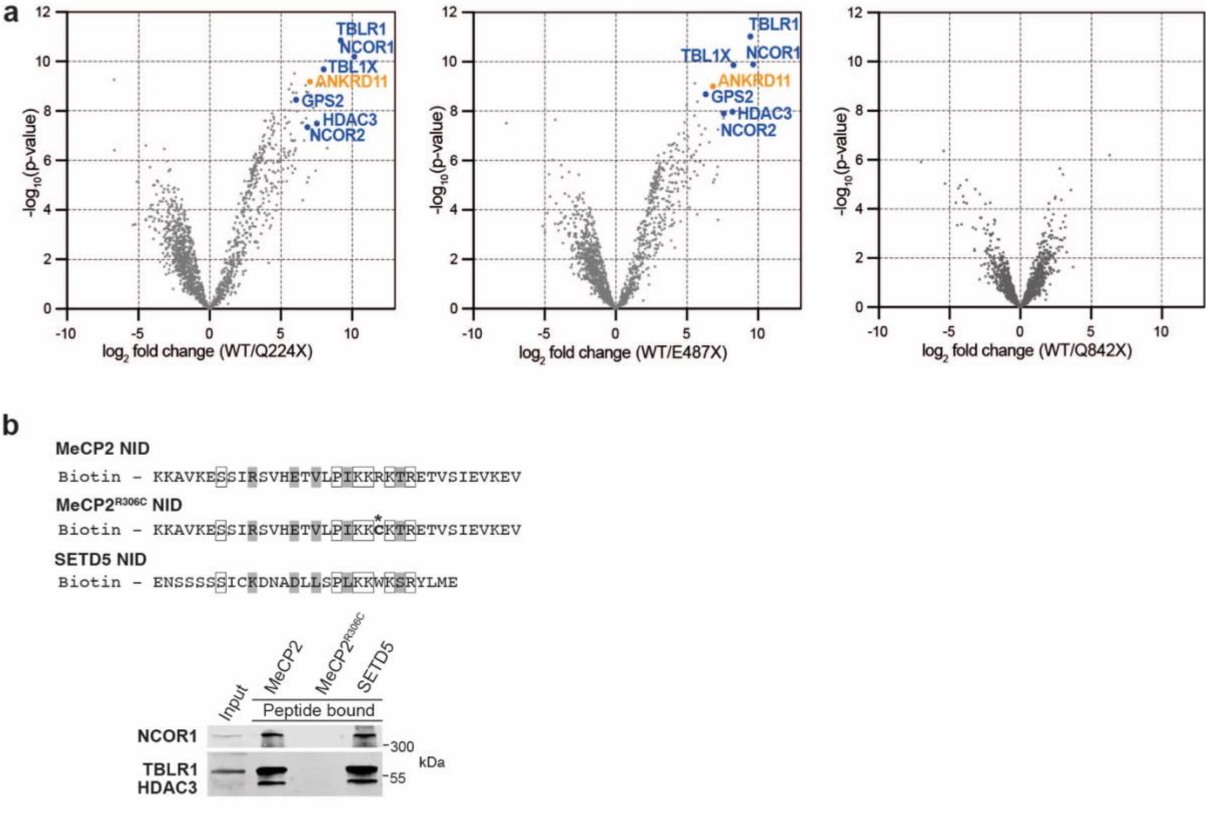

Supplementary Figure 3: The NID motif in SETD5 mediates the interaction with SET3C.

a, Volcano plots showing enrichment of protein interactions detected by mass spectrometry following immunoprecipitation of wild-type (WT) EGFP-SETD5 or indicated truncation constructs expressed in TBLR1^{WT} Flp-In™ T-REx™ 293 cells (n = 3 independent transfections). NCoR complex core components (blue) and ANKRD11 (orange) are labelled. Statistical significance was calculated using two-tailed moderated t-tests. **b,** Western blot for NCoR1, TBLR1 and HDAC3 after peptide pull-downs from wild-type mouse brain extracts. Sequences of the biotinylated peptides are shown with identical (white box) and similar (grey) amino acids indicated. Asterisks = R306C Rett Syndrome causing mutation in MeCP2 which is known to abolish TBLR1 binding. NID = NCoR interaction domain.

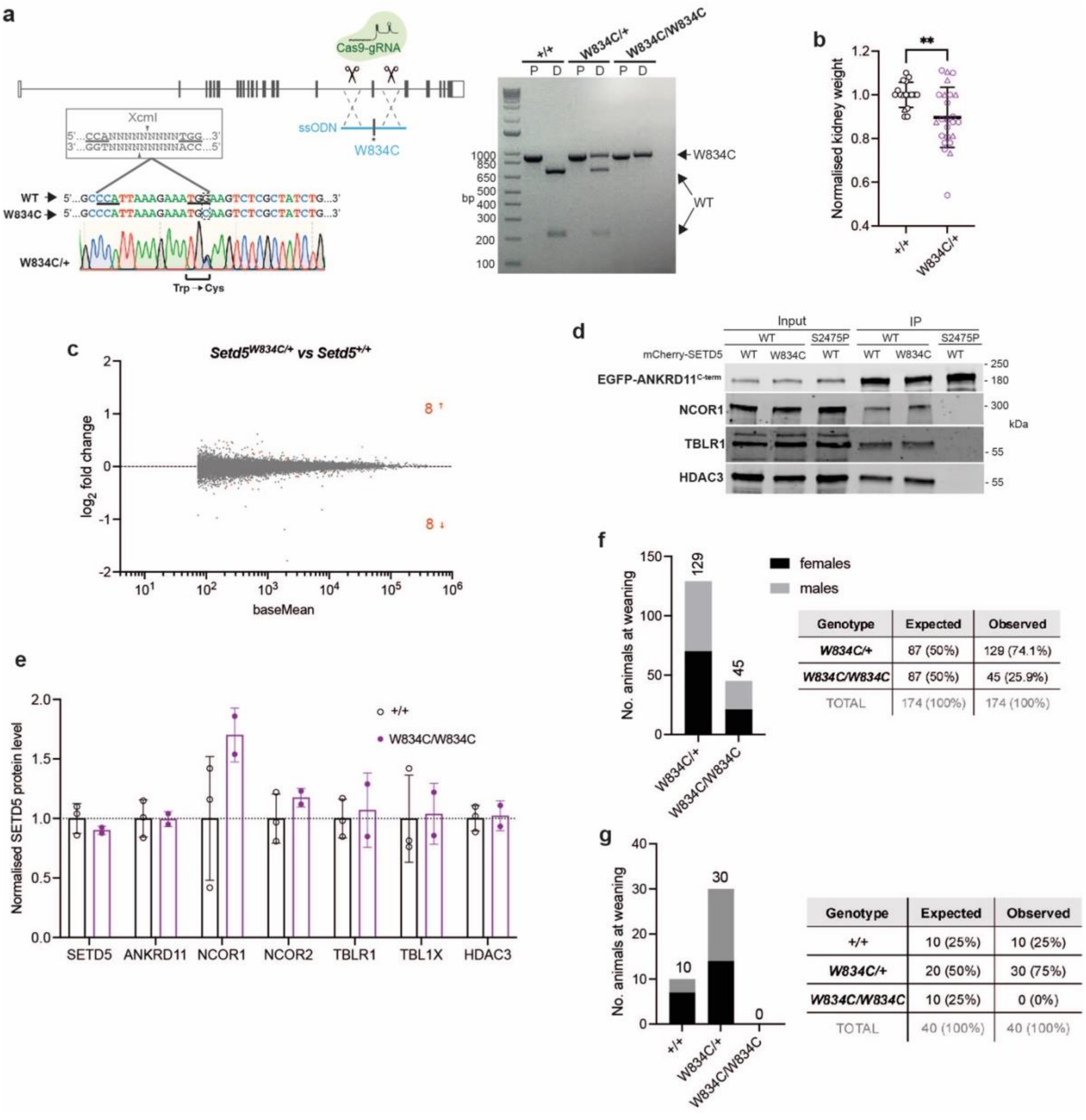

Supplementary Figure 4: Generation and characterisation of the *Setd5*^{W834C} mouse model.

a, Left: Targeting strategy to generate the heterozygous *Setd5*$^{W834C/+}$ embryonic stem cells used to make mice. A Sanger sequencing chromatogram of a PCR amplicon of the targeted region from a heterozygous sample is shown. The W834C mutation destroys an XcmI restriction enzyme site. Right: The genotyping strategy used to distinguish *Setd5*$^{+/+}$, *Setd5*$^{W834C/+}$ and *Setd5*$^{W834C/W834C}$ mice. Gel electrophoresis of PCR samples before and after XcmI digestion (P = PCR, D = digested). **b,** Kidney weight of heterozygous *Setd5*$^{W834C/+}$ mice (n = 24; n = 16 males (circles), n = 8 females (triangles)) and their wild-type *Setd5*$^{+/+}$ littermates (n = 17; n = 11 males (circles), n = 6 females (triangles)). Values were normalised to the average of the wild-type littermates of the same sex. Mean and +/- SD are indicated. Statistical significance was calculated using a two-tailed unpaired t-test with Welch's correction (kidney weight **p = 0.0023). **c**, MA plots of gene expression changes in cortical tissue from 4-week-old *Setd5*$^{W834C/+}$ and *Setd5*$^{+/+}$ mice. Genes with significantly increased or decreased expression levels (p adj < 0.05) are shown in red (n = 6 mice for each genotype, n = 3 females, n = 3 males). Statistical significance was calculated using two-tailed Wald test using Benjamini-Hochberg adjustment for multiple comparisons. **d**, Western blot analysis of SET3C components bound following immunoprecipitation of EGFP-ANKRD11$^{C\text{-term}}$ from extracts from EGFP-ANKRD11$^{C\text{-term}}$ (WT or S2475P) transfected wild-type (+/+) vs. Setd5$^{W834C/W834C}$ (W834C) ESCs. **e,** Mass spectrometry analysis of protein levels of SET3C components in extracts from wild-type and W834C homozygous mouse embryonic stem cells (*Setd5*$^{+/+}$ n = 3 separate clones, *Setd5*$^{W834C/W834C}$ n = 2 separate clones). Mean and +/- SD are indicated. **f,** Numbers of *Setd5*$^{W834C/W834C}$ and *Setd5*$^{W834C/+}$ offspring of weaning age from *Setd5*$^{W834C/W834C}$ X *Setd5*$^{W834C/+}$ crosses (on a mixed background). Statistical significance was calculated using a two-tailed binomial test (****p = 1x10^{-10}). **g**, Numbers of *Setd5*$^{W834C/W834C}$, *Setd5*$^{W834C/+}$ and *Setd5*$^{+/+}$ offspring at weaning from *Setd5*$^{W834C/+}$ X *Setd5*$^{W834C/+}$ crosses of inbred mice (>6 backcrosses to C57BL/6J mice). Statistical significance was calculated using a two-tailed chi-squared test (***p = 0.0006).

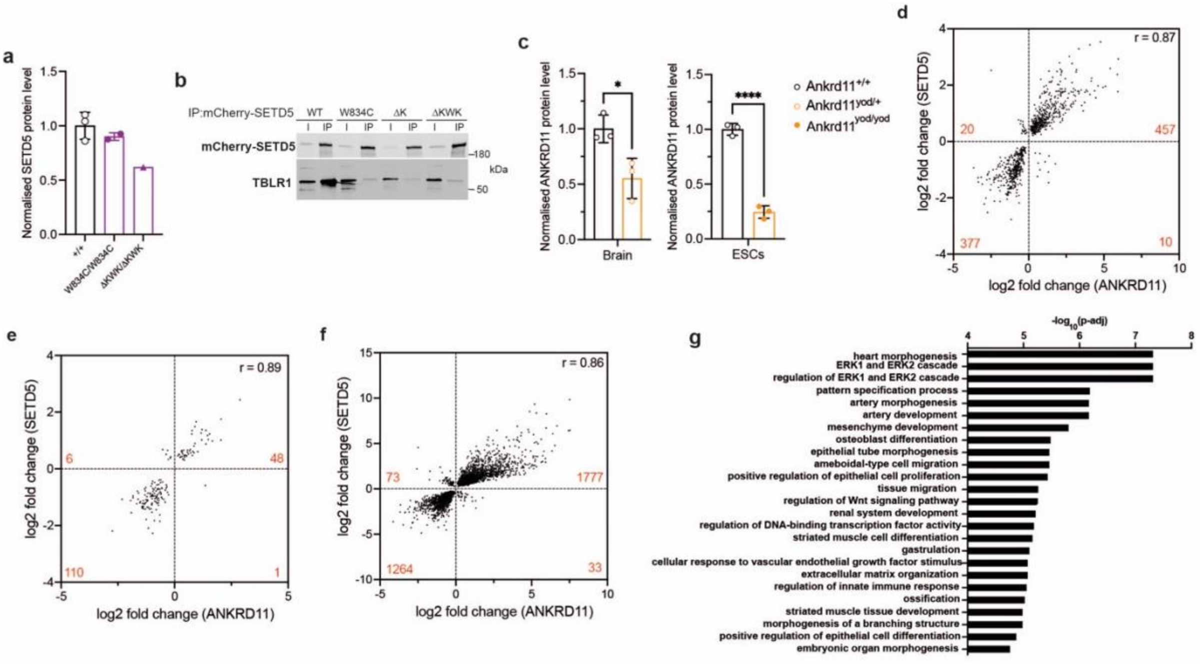

Supplementary Figure 5: **The SETD5 NID mutations destroy binding to TBLR1 without substantially affecting protein stability.**

a, Mass spectrometry analysis of SETD5 protein levels in extracts from wild-type, W834C homozygous and ΔKWK homozygous mouse embryonic stem cells (*Setd5*$^{+/+}$ n = 3 separate clones, *Setd5*$^{W834C/W834C}$ n = 2 separate clones, Setd5$^{\Delta KWK/\Delta KWK}$ n = 1 clone). Mean and +/- SD are indicated. **b,** Western blot for TBLR1 and mCherry after immunoprecipitation of wild-type (WT) and mutant mCherry-SETD5 expressed in TBLR1^{WT} Flp-In™ T-REx™ 293 cells. **c,** Mass spectrometry quantification of ANKRD11 protein levels in nuclear extracts from Yoda heterozygous male mouse brain (n = 3 mice for each genotype) and whole cell extracts from Yoda homozygous embryonic stem cells (ESCs) (n = 3 separate clones for each genotype). Mean and +/- SD are indicated. Statistical significance was calculated using a two-tailed unpaired t-test (Brains *p = 0.0242, ESCs ****p = 0.0000729). **d,** Scatterplot showing gene expression changes in *Ankrd11* and *Setd5* mutant mouse embryonic stem cells (Pearson r = 0.87) (n = 3 independent clones for each genotype). Points correspond to genes which are differentially expressed in both mutants (p adj < 0.01). Statistical significance was calculated using two-tailed Wald test using Benjamini-Hochberg adjustment for multiple comparisons. **e,** Scatterplot showing gene expression changes in *Ankrd11*$^{yod/yod}$ and *Setd5*$^{W834C/W834C}$ mutant mouse embryonic stem cells (Pearson r = 0.89) (Excluding ΔKWK *Setd5* mutant ES cell clone; n = 2 separate clones per genotype and n = 3 separate outgrowths per clone). Points correspond to genes which are differentially expressed in both genotypes (p adj < 0.05). Statistical significance was calculated using two-tailed Wald test using Benjamini-Hochberg adjustment for multiple comparisons. **f,** Scatterplot showing gene expression changes in *Ankrd11*$^{yod/yod}$ and *Setd5*$^{\Delta KWK/\Delta KWK}$ mutant mouse embryonic stem cells (Pearson r = 0.86) (*Ankrd11*$^{yod/yod}$ n = 3 separate clones and n = 3 separate outgrowths per clone, *Setd5*$^{\Delta KWK/\Delta KWK}$ n = 1 separate clone n = 3 separate outgrowths per clone). Points correspond to genes which are differentially expressed in both genotypes (p adj < 0.05). Statistical significance was calculated using two-tailed Wald test using Benjamini-Hochberg adjustment for multiple comparisons. **g,** GO term enrichment analysis of shared differentially expressed genes in *Setd5* and *Ankrd11* mutant mouse embryonic stem cells. Statistical significance was calculated using the one-tailed hypergeometric test using Benjamini-Hochberg adjustment for multiple comparisons.

Uncropped western blot for Supplementary Figure 1a.

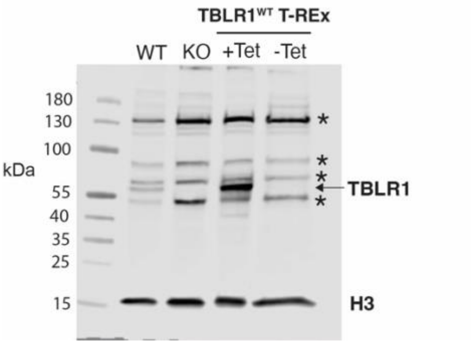

Uncropped western blot for Supplementary Figure 1c.

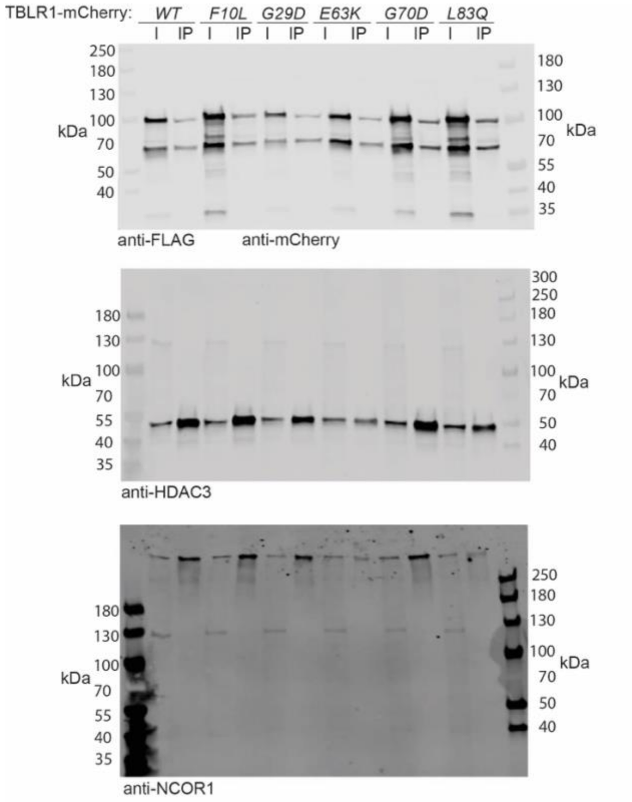

Uncropped western blot for Supplementary Figure 1d.

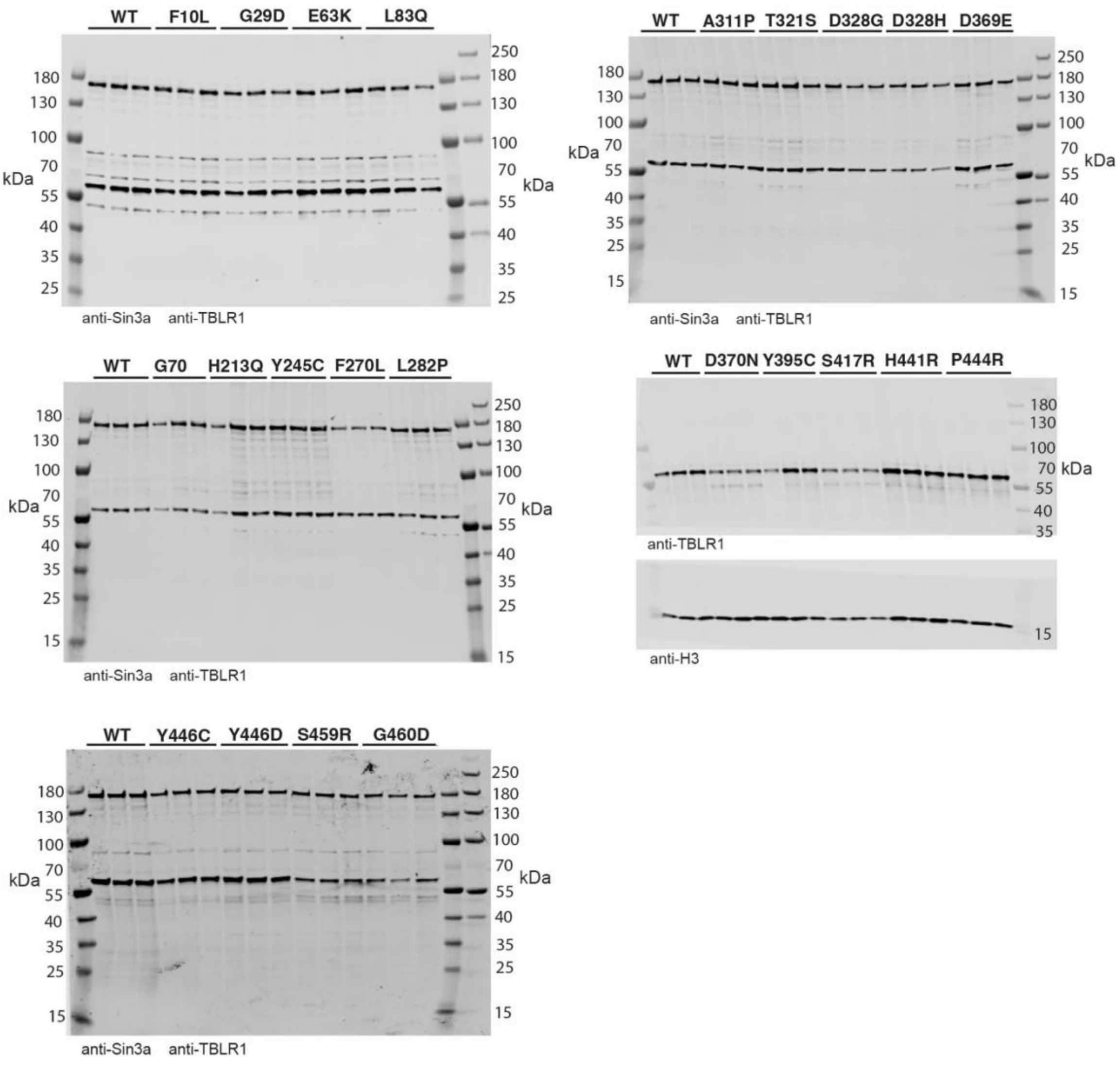

Uncropped western blot for Supplementary Figure 3b.

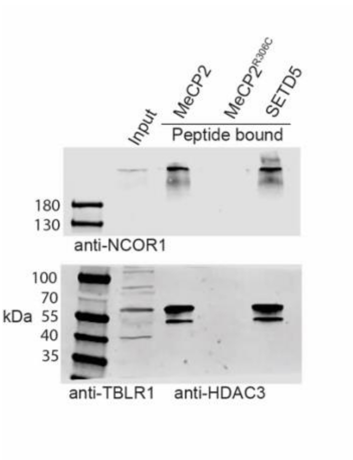

Uncropped western blot for Supplementary Figure 4d.

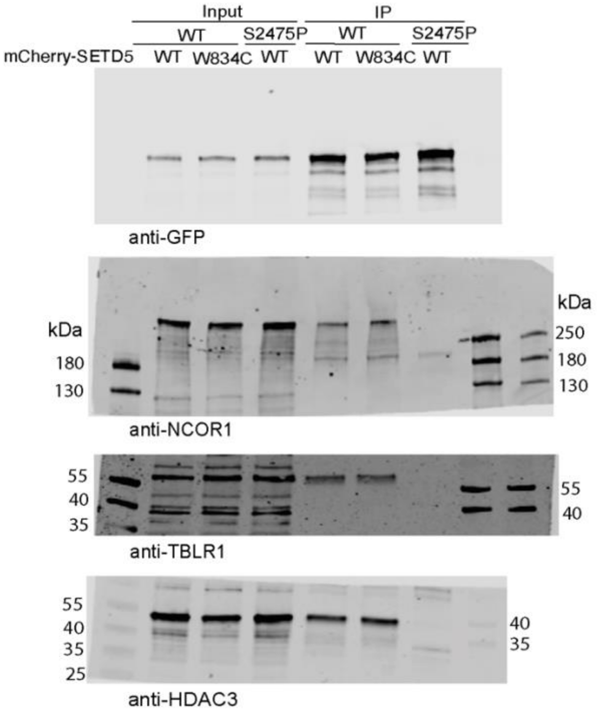

Uncropped western blot for Supplementary Figure 5b.

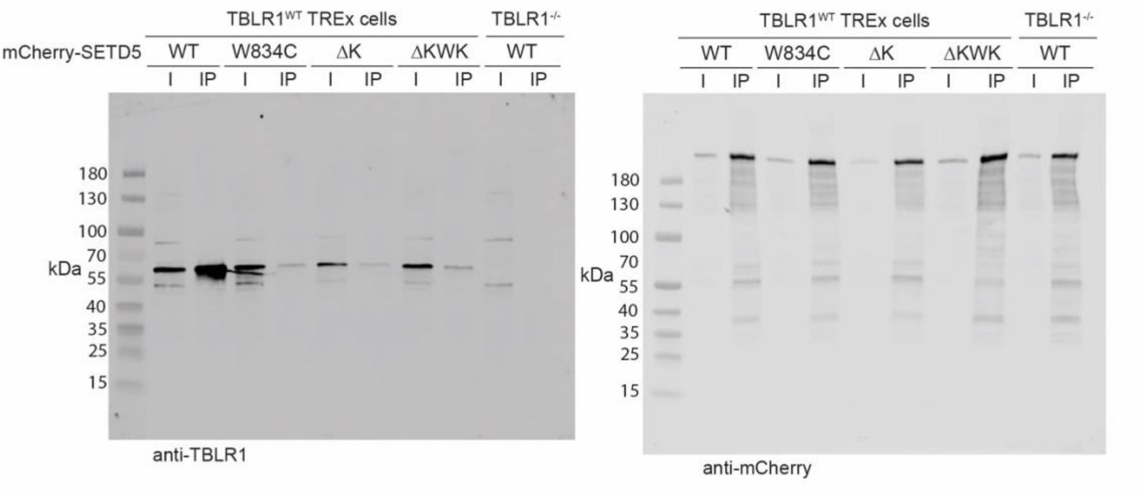

Supplement: Supplementary file 1 — Supplementary Information [file 41467_2026_73227_MOESM1_ESM.pdf]
