# Supplementary material for: Dysfunction of a SET3-like complex underlies a family of related neurological disorders
Source: Nat Commun. 2026 May 16;17:6729. doi: 10.1038/s41467-026-73227-5 (PMC13385800; doi:10.1038/s41467-026-73227-5)
Supplement: Supplementary file 2 — Description of Additional Supplementary Files [file 41467_2026_73227_MOESM2_ESM.pdf]

Description of Additional Supplementary Files

Title: Supplementary Data 1

Description:

Sheet 1) Full plasmid sequences. Sheet 2) Primer sequences used for mutagenesis and Gibson assembly to generate plasmid constructs. Sheet 3) CRISPR-Cas9 guide RNA sequences and homology repair template DNA sequences used to generate cell lines. Sheet 4) Peptide sequences used for peptide pull-downs. Sheet 5) Genotyping primer sequences.
