# Supplementary material for: Dysfunction of a SET3-like complex underlies a family of related neurological disorders
Source: Nat Commun. 2026 May 16;17:6729. doi: 10.1038/s41467-026-73227-5 (PMC13385800; doi:10.1038/s41467-026-73227-5)
Supplement: Supplementary file 4 — Reporting Summary [file 41467_2026_73227_MOESM4_ESM.pdf]

nature portfolio

Corresponding author(s): Adrian Bird and Matthew Lyst

Last updated by author(s): Apr 1, 2026

Reporting Summary

Nature Portfolio wishes to improve the reproducibility of the work that we publish. This form provides structure for consistency and transparency in reporting. For further information on Nature Portfolio policies, see our Editorial Policies and the Editorial Policy Checklist.

Statistics

For all statistical analyses, confirm that the following items are present in the figure legend, table legend, main text, or Methods section.

n/a	Confirmed	
☐	☒	The exact sample size (n) for each experimental group/condition, given as a discrete number and unit of measurement
☐	☒	A statement on whether measurements were taken from distinct samples or whether the same sample was measured repeatedly
☐	☒	The statistical test(s) used AND whether they are one- or two-sided<br>*Only common tests should be described solely by name; describe more complex techniques in the Methods section.*
☒	☐	A description of all covariates tested
☐	☒	A description of any assumptions or corrections, such as tests of normality and adjustment for multiple comparisons
☐	☒	A full description of the statistical parameters including central tendency (e.g. means) or other basic estimates (e.g. regression coefficient) AND variation (e.g. standard deviation) or associated estimates of uncertainty (e.g. confidence intervals)
☐	☒	For null hypothesis testing, the test statistic (e.g. F, t, r) with confidence intervals, effect sizes, degrees of freedom and P value noted<br>*Give P values as exact values whenever suitable.*
☒	☐	For Bayesian analysis, information on the choice of priors and Markov chain Monte Carlo settings
☒	☐	For hierarchical and complex designs, identification of the appropriate level for tests and full reporting of outcomes
☐	☒	Estimates of effect sizes (e.g. Cohen's d, Pearson's r), indicating how they were calculated

Our web collection on statistics for biologists contains articles on many of the points above.

Software and code

Policy information about availability of computer code

Data collection	Western blot Data was collected using LI-COR Odyssey CLx system and Image Studio Lite software v5.2.5 (LI-COR biosciences).
Data analysis	Data was analysed and Statistical test were performed using GraphPad Prism v10.6.1. Mass spectrometry data was analysed using MaxQuant82 v1.6.1.0, DIA-NN software platform v1.8.1 and DEP package v1.26.0 in R v4.4.0. RNA-seq data was analysed using DESeq2 v1.42.0. Gene Ontology analysis was performed using clusterProfiler v4.8.1. Alphafold 3 was used to analyse predicted protein structures. SnapGene v4.3.11 was used to analyse DNA sequencing Data. Code for RNA-seq and differential gene expression analysis is available at https://github.com/kashyapchhatbar/SET3C-manuscript-2026

For manuscripts utilizing custom algorithms or software that are central to the research but not yet described in published literature, software must be made available to editors and reviewers. We strongly encourage code deposition in a community repository (e.g. GitHub). See the Nature Portfolio guidelines for submitting code & software for further information.

Data

Policy information about availability of data

All manuscripts must include a data availability statement. This statement should provide the following information, where applicable:

- Accession codes, unique identifiers, or web links for publicly available datasets
- A description of any restrictions on data availability
- For clinical datasets or third party data, please ensure that the statement adheres to our policy

Raw data for Figures and associated Supplementary Figures are provided in Source data Zip file including uncropped western blot images, formatted and uncropped

western blots for replicate experiments and datasheets containing individual values underlying each plot. The mass spectrometry data generated in this study have been deposited in the PRIDE partner repository under the accession codes PXD063846 [https://www.ebi.ac.uk/pride/archive/projects/PXD063846] and PXD077916 [https://www.ebi.ac.uk/pride/archive/projects/PXD077916]. The RNA-sequencing data generated in this study have been deposited in the ArrayExpress database under the accession codes E-MTAB-15318 [https://www.ebi.ac.uk/biostudies/arrayexpress/studies/E-MTAB-15318] (ES cells) and E-MTAB-16803 [https://www.ebi.ac.uk/biostudies/arrayexpress/studies/E-MTAB-16803] (cortex). The Perturb-seq data used in this study are available in Figshare Plus under DOI 10.25452/figshare.plus.21632564.v1 [https://doi.org/10.25452/figshare.plus.21632564.v1] (p-values) and 10.25452/figshare.plus.20029387 [https://doi.org/10.25452/figshare.plus.20029387] (Z-scores).

Research involving human participants, their data, or biological material

Policy information about studies with human participants or human data. See also policy information about sex, gender (identity/presentation), and sexual orientation and race, ethnicity and racism.

Reporting on sex and gender	N/A - no human subjects
Reporting on race, ethnicity, or other socially relevant groupings	N/A - no human subjects
Population characteristics	N/A - no human subjects
Recruitment	N/A - no human subjects
Ethics oversight	N/A - no human subjects

Note that full information on the approval of the study protocol must also be provided in the manuscript.

Field-specific reporting

Please select the one below that is the best fit for your research. If you are not sure, read the appropriate sections before making your selection.

[x] Life sciences [ ] Behavioural & social sciences [ ] Ecological, evolutionary & environmental sciences

For a reference copy of the document with all sections, see nature.com/documents/nr-reporting-summary-flat.pdf

Life sciences study design

All studies must disclose on these points even when the disclosure is negative.

Sample size	Sample size for mouse phenotypic scoring experiments were selected based on our previous studies of Mecp2 mutant mice and practical considerations of animal availability; no formal power calculation was performed. For other experiments sample sizes were chosen as to be in line with other similar studies in the field and to allow for robust statistical analysis. No sample size calculations were performed.
Data exclusions	Mice which died for reasons unrelated to the Rett-like phenotype of MeCP2 mutants (such as fighting) were removed from the survival analysis.
Replication	We performed replicates of all experiments (see source data). No experiments failed to reproduce. Mass spectometry experiments were performed with 3 independent replicates. RNA-sequencing experiments were performed with 6 independent replicates per genotype. Mouse phenotypic scoring experiments were performed with a minimum of 14 biological replicates. Mouse weight/length analysis was performed on 8-24 biological replicates. Immunoprecipitation followed by western blot analysis was repeated at least twice. The NanoLuc complementation assay was performed with 3-5 independent replicates.
Randomization	N/A - experimental groups were wild-types vs. mutants.
Blinding	Investigators were blinded to the genotype of animals in our phenotypic scoring experiments. For other assays blinding was not applicable as these assays were quantitative and not subjective.

Reporting for specific materials, systems and methods

We require information from authors about some types of materials, experimental systems and methods used in many studies. Here, indicate whether each material, system or method listed is relevant to your study. If you are not sure if a list item applies to your research, read the appropriate section before selecting a response.

Materials & experimental systems

n/a	Involved in the study
☐	☒ Antibodies
☐	☒ Eukaryotic cell lines
☒	☐ Palaeontology and archaeology
☐	☒ Animals and other organisms
☒	☐ Clinical data
☒	☐ Dual use research of concern
☒	☐ Plants

Methods

n/a	Involved in the study
☒	☐ ChIP-seq
☒	☐ Flow cytometry
☒	☐ MRI-based neuroimaging

Antibodies

Antibodies used

HDAC3 (Abnova, H00008841-M02, lot 15281-3E11, clone 3E11)
HDAC3 (Abcam, ab32369, lot 1002834-27)
mCherry (Abcam, ab167453, lot 61100)
mCherry (Abcam, ab125096, lot GR309694-1, clone 1C51)
NCoR1 (Cell Signalling Technology, 5948S, lot 2)
GFP (Takara, 632592, lot 2310030)
GFP (Takara, 632381, lot A8034133, clone JL-8)
SIN3A (Abcam, ab3479, lot 1070214)
TBLR1 (Santa Cruz Biotechnology, sc-100908, lot J0115, clone L-08)
γ-tubulin (Sigma, T5326, lot 0000299270)
Histone H3 (Abcam ab1791, lot GR3421644-1)

Antibodies dilutions used for western blots were 1:1000 for all antibodies except H3 which was 1:10,000

Validation

HDAC3 (Abnova, H00008841-M02, lot 15281-3E11, clone 3E11) - tested in western blot by the manufacturer, epitope conserved in human and mouse (thermofisher.com).

HDAC3 (Abcam, ab32369, lot 1002834-27) - tested by the manufacturer in western blots against mouse and human samples, cited in 92 publications (abcam.com).

mCherry (Abcam, ab167453, lot 61100) - tested in western blot by the manufacturer, and used in 631 publications (abcam.com). We confirmed specificity in our assay using transfection of mCherry fused to different length fragments of SETD5.

mCherry (Abcam, ab125096, lot GR309694-1, clone 1C51) - tested in western blot by the manufacturer, and used in 180 publications (abcam.com). We confirmed specificity in our assay using a control without mCherry-SETD5 transfection.

NCoR1 (Cell Signalling Technology, 5948S, lot 2) - tested in Western blot by the manufacturer on mouse and human extracts. Used in 39 publications (https://www.cellsignal.com/). Confirmed to immunoprecipitate the NCoR complex by our mass spectrometry.

GFP (Takara, 632592, lot 2310030) - tested for western blotting by the manufacturer. We confirmed specificity by western blotting GFP-containing fusion proteins of different sizes (www.takarabio.com).

GFP (Takara, 632381, lot A8034133, clone JL-8) - tested in western blotting by the manufacturer. We confirmed specificity by western blotting GFP-containing fusion proteins of different sizes (www.takarabio.com).

SIN3A (Abcam, ab3479, lot 1070214) - tested in western blotting with human samples by the manufacturer, and used in 55 publications (abcam.com).

TBLR1 (Santa Cruz Biotechnology, sc-100908, lot J0115, clone L-08) - used in 13 publications (www.scbt.com) and specificity in western blots confirmed using our TBLR1 knockout TREx cell line

γ-tubulin (Sigma, T5326, lot 0000299270) - reactivity in human and mouse according to the manufacturer, and has 365 citations (sigmaaldrich.com)

Histone H3 (Abcam ab1791, lot GR3421644-1) - validated by the manufacturer for western blotting in human and mouse, and cited in 4740 publications (abcam.com)

Eukaryotic cell lines

Policy information about cell lines and Sex and Gender in Research

Cell line source(s)

Embryonic stem cells (ESCs) 129/Ola E14TG2a (Mus musculus, Sex: male, Source: A gift from Joe Mee from th University of Edinburgh)
Flp-In™ T-REx™ 293 cells (Human, Sex: female, Source: Thermo, R78007)

	HEK293 cells (Human, Sex: female, Source: ATCC, CRL-1573)
Authentication	Mouse ESCs were authenticated by the successful generation of mutant mice through blastocyst injection of modified ESCs. Flp-In™ T-REx™ 293 cells and HEK293 cells were purchased from commercial companies which ensure authentication.
Mycoplasma contamination	All cell lines tested negative for Mycoplasma contamination.
Commonly misidentified lines (See ICLAC register)	No common misidentified lines were used.

Animals and other research organisms

Policy information about studies involving animals; ARRIVE guidelines recommended for reporting animal research, and Sex and Gender in Research

Laboratory animals	Mus Musculus species of mice were used in this study. The Setd5W834C mice were generated by injection of heterozygous Setd5W834C/+ ESCs (129/Ola E14TG2a) into E3.5 blastocysts obtained from C57BL/6J (Charles River RRID:IMSR_JAX:000664) females after natural matings. Blastocysts were transferred to pseudo-pregnant recipient females and chimeric offspring were mated to C57BL/6J mice to establish the line. The Ankrd11Yod mice (C3H.Cg-Ankrd11Yod/H) were rederived from mouse sperm purchased from the EMMA mouse repository (EM:00380). Mice were bred and maintained at the University of Edinburgh animal facility under standard conditions with 12h dark/light cycles, an ambient temperature 20-24 degrees Celcius and relative humidity of 45-65%. All procedures were carried out by staff licensed by the UK Home Office and in accordance with the Animal and Scientific Procedures Act 1986. Weight and length measurements and tissues for molecular analysis were taken from 6 week-old mice. Cortical brain tissue for RNA-seq analysis was taken from 4-week old mice which were backcrossed to C57BL/6J mice for n = 5 generations.
Wild animals	No wild animals were used in this study.
Reporting on sex	Sex was considered a key parameter in the phenotypic analysis of Setd5W834C mice compared to their wild-type littermates (weight measurements and survival to weaning) and mass spectrometry quantification of protein levels in brain extracts, as the results of these experiments could be influenced by sex. We therefore included roughly equal numbers of each sex for these experiments and found that findings applied to both sexes. Immunoprecipitation of NCOR1 from brain extracts to analyse protein-protein interactions was performed using samples from male mice only as these findings should not be influenced by sex. For experiments analysing the phenotype of Mecp2-/y, Setd5W834C/+ compound mutant mice only males were analysed as the Mecp2 gene is X-linked and hemizygous male mice have a much more severe phenotype than female mice. It was therefore only appropriate to compare mutant male mice to their control male littermates for this experiment. Overall numbers of each sex: females: 200, males: 262. We show the male and female distributions for each experiment using shape-coding or stacked bar charts, and the numbers of each sex are stated in the figure legends. Disaggregated data for sex is provided in source data.
Field-collected samples	No-field collected samples were used in this study.
Ethics oversight	Procedures were carried out by certified persons, licensed by the UK Home Office and according to the Animals (Scientific Procedures) Act 1986 under project licences (PPLs) 60/4547 and PP4326006. Ethical review of the PPLs was carried out by the University of Edinburgh Animal Welfare and Ethical Review Board (AWERB).

Note that full information on the approval of the study protocol must also be provided in the manuscript.

Plants

Seed stocks	N/A
Novel plant genotypes	N/A
Authentication	N/A
