# Supplementary material for: Dysfunction of a SET3-like complex underlies a family of related neurological disorders
Source: Nat Commun. 2026 May 16;17:6729. doi: 10.1038/s41467-026-73227-5 (PMC13385800; doi:10.1038/s41467-026-73227-5)
Supplement: Supplementary file 6 — Source Data [file 41467_2026_73227_MOESM6_ESM.zip › Source data/Western blots - uncropped images and replicates/Replicate western blots/Formatted western blots_replicate experiments.docx]

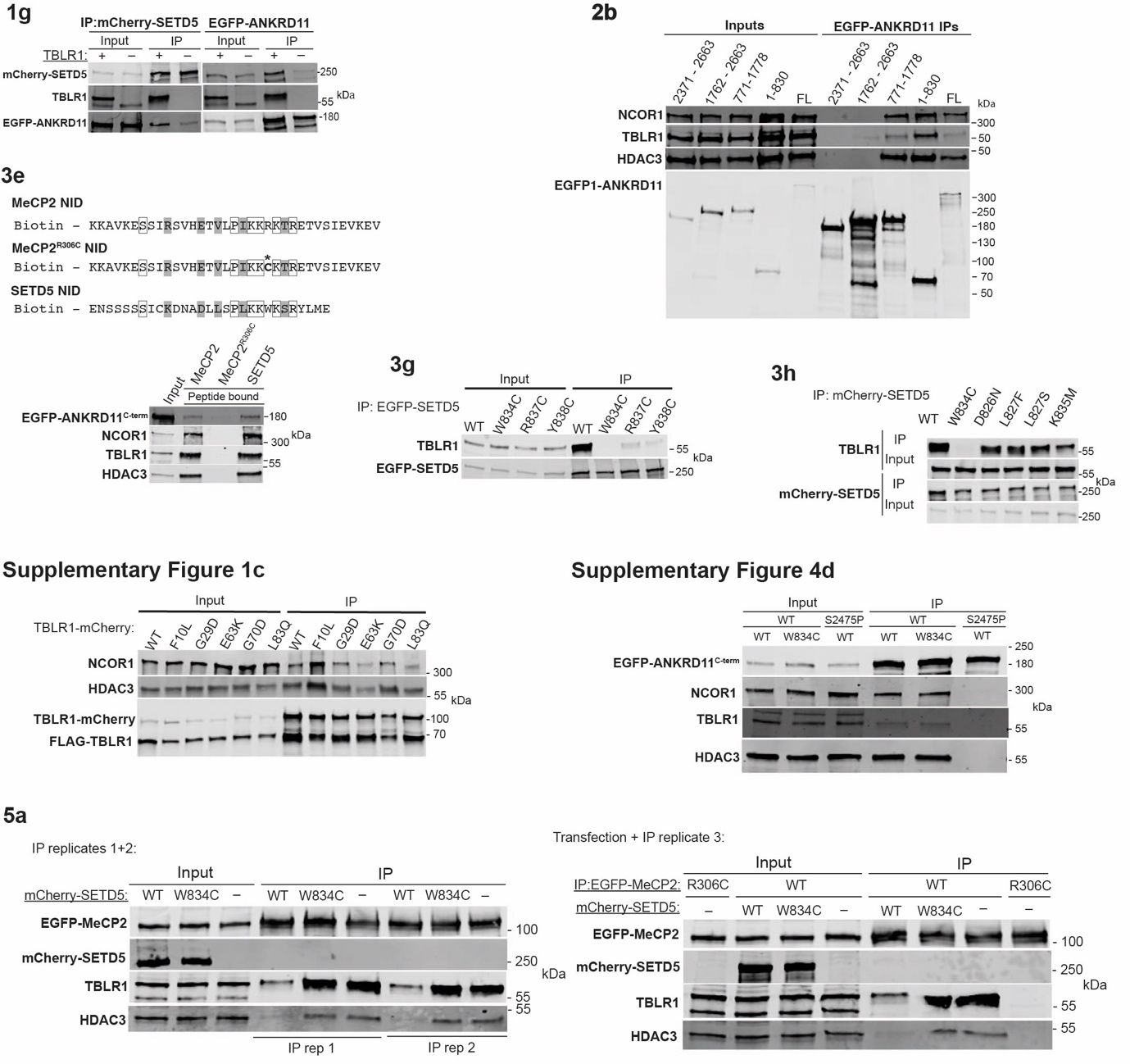


**1g,** Western blot for TBLR1, HDAC3, mCherry and EGFP following immunoprecipitation of mCherry-SETD5 or EGFP-ANKRD11^C-term^ in either TBLR1-null or TBLR1^WT^ Flp-In™ T-REx™ 293 cells.

**2b,** Western blot for TBLR1, NCoR1 and EGFP following immunoprecipitation of EGFP-ANKRD11 full length (FL) or truncation constructs from TBLR1^WT^ Flp-In™ T-REx™ 293 cells.

**3e,** Western blot for EGFP, TBLR1 and HDAC3 after peptide pull-downs from EGFP-ANKRD11^C-term^ transfected TBLR1^WT^ Flp-In™ T-REx™-293 cell lysates. Sequences of the biotinylated peptides are shown with identical (white box) and similar (grey) amino acids indicated. * = R306C Rett Syndrome causing mutation in MeCP2 which is known to abolish TBLR1 binding.

**3g,** Western blot for TBLR1 and EGFP following immunoprecipitation of wild-type (WT) or pathogenic mutant (R837C and Y838C) EGFP-SETD5 expressed in TBLR1^WT^ Flp-In™ T-REx™ 293 cells. The W834C mutation is used as a negative control.

3**h,** Western blot for TBLR1 and mCherry following immunoprecipitation of wild-type (WT) and mutant mCherry-SETD5 expressed inTBLR1^WT^ Flp-In™ T-REx™ 293 cells. The mutations tested are from gnomAD v2.1.1, v3.1.2 and ExAC (excluding mutations also in ClinVar).The W834C mutation is used as a negative control.

**S1c,** Western blot for NCoR1, HDAC3, mCherry and FLAG following immunoprecipitation of wild-type (WT) or indicated mutated forms of TBLR1-mCherry from TBLR1^WT^ Flp-In™ T-REx™ 293 cells that were co-transfected with TBLR1-mCherry and FLAG-TBLR1.

**S4d,** Western blot analysis of SET3C components bound following immunoprecipitation of EGFP-ANKRD11^C-term^ from extracts from EGFP-ANKRD11^C-term^ (WT or S2475P) transfected wild-type (+/+) vs. Setd5^W834C/W834C^ (W834C) ESCs. **d,** Mass spectrometry analysis of protein levels of SET3C components in extracts from wild-type and W834C homozygous mouse embryonic stem cells.

**5a,** Western blot for TBLR1, HDAC3, mCherry and EGFP following immunoprecipitation of EGFP-MeCP2 from mixed EGFP-MeCP2 (WT or R306C) and mCherry-SETD5 (WT or W834C) transfected HEK293T cell extracts. Cell extracts were mixed at a ratio of 20:1 mCherry-SETD5 to EGFP-MeCP2. Left panel: 2 replicate IPs using same starting cell extracts. Right panel: a third replicate, new transfections and IPs including EGFP-MeCP2^R306C^.
