# Supplementary figures and images for: Dysfunction of a SET3-like complex underlies a family of related neurological disorders
Source: Nat Commun. 2026 May 16;17:6729. doi: 10.1038/s41467-026-73227-5 (PMC13385800; doi:10.1038/s41467-026-73227-5)

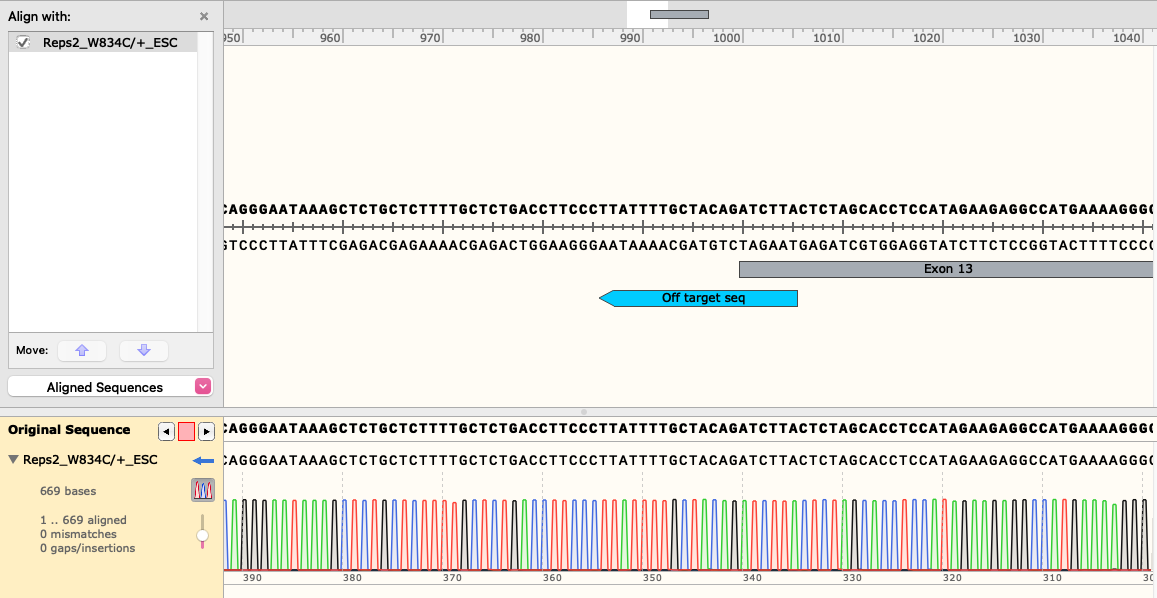

Supplement: Supplementary file 6 — Source Data [file 41467_2026_73227_MOESM6_ESM.zip › Source data/CRISPR off-targets sequencing/chromatograms/SEQ CRISPR off-target_Reps2_ESCs .png]

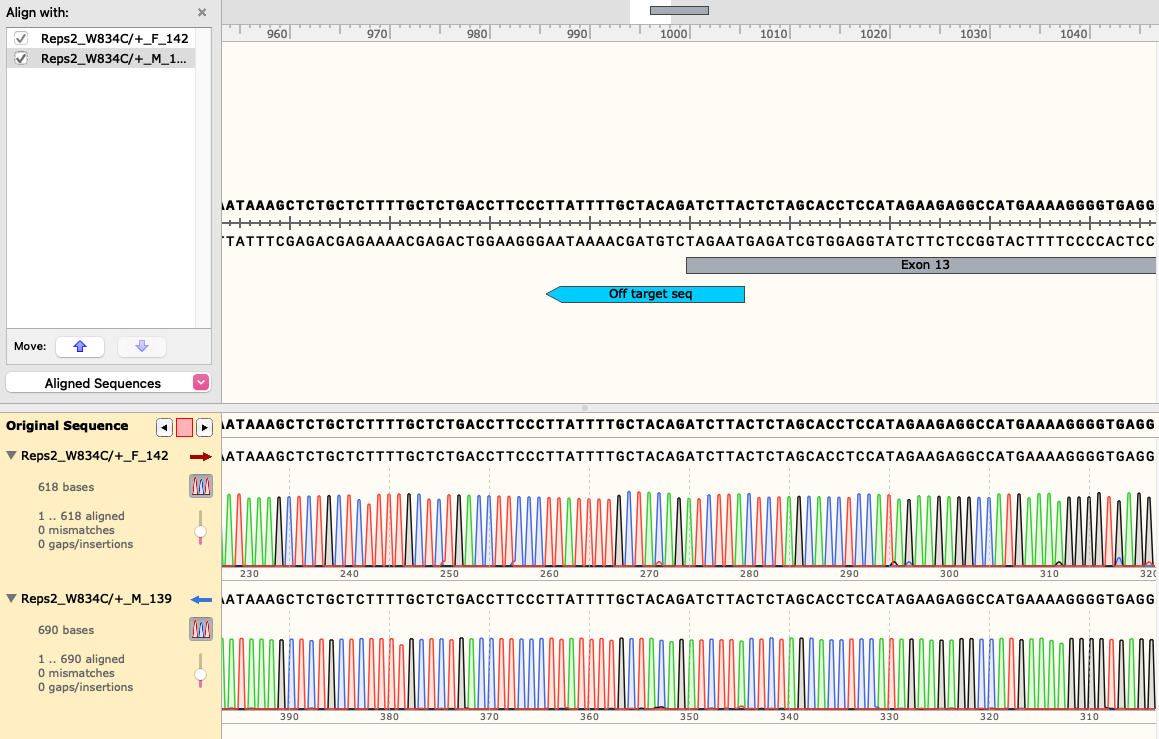

Supplement: Supplementary file 6 — Source Data [file 41467_2026_73227_MOESM6_ESM.zip › Source data/CRISPR off-targets sequencing/chromatograms/SEQ CRISPR off-target_Reps2_het mouse.png]

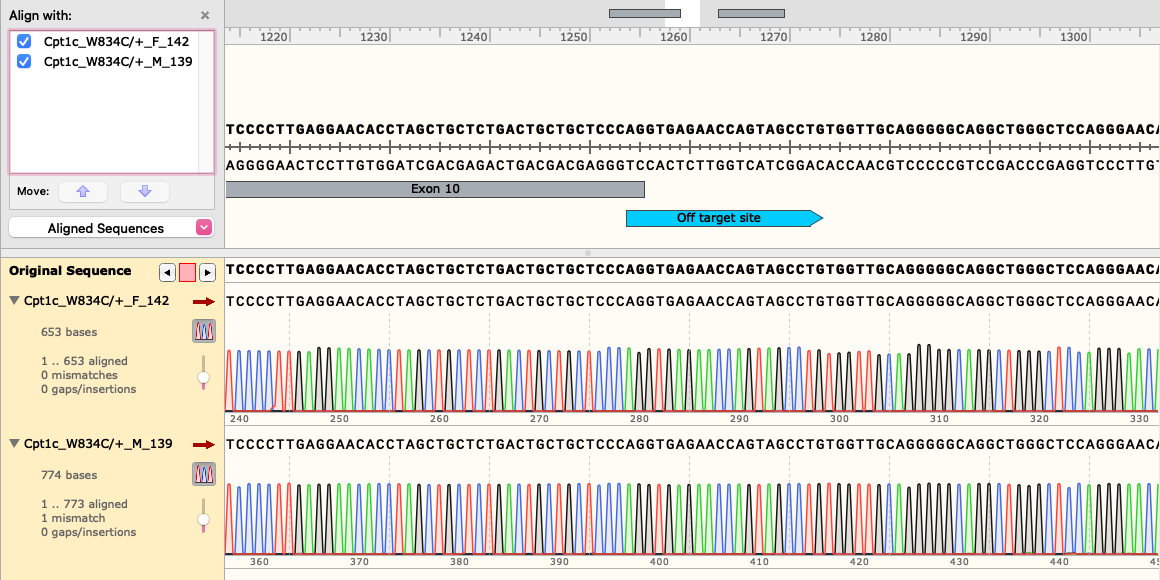

Supplement: Supplementary file 6 — Source Data [file 41467_2026_73227_MOESM6_ESM.zip › Source data/CRISPR off-targets sequencing/chromatograms/SEQ CRISPR off-target_Cpt1c_het mouse.png]

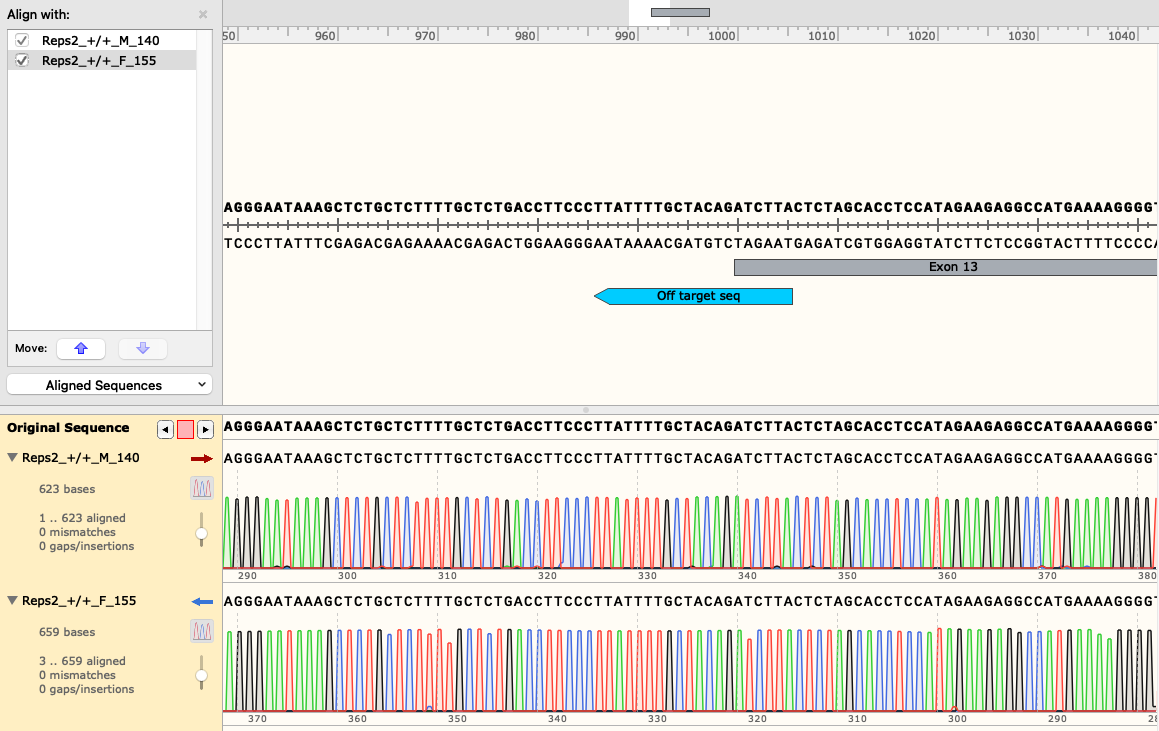

Supplement: Supplementary file 6 — Source Data [file 41467_2026_73227_MOESM6_ESM.zip › Source data/CRISPR off-targets sequencing/chromatograms/SEQ CRISPR off-target_Reps2_WT mouse.png]

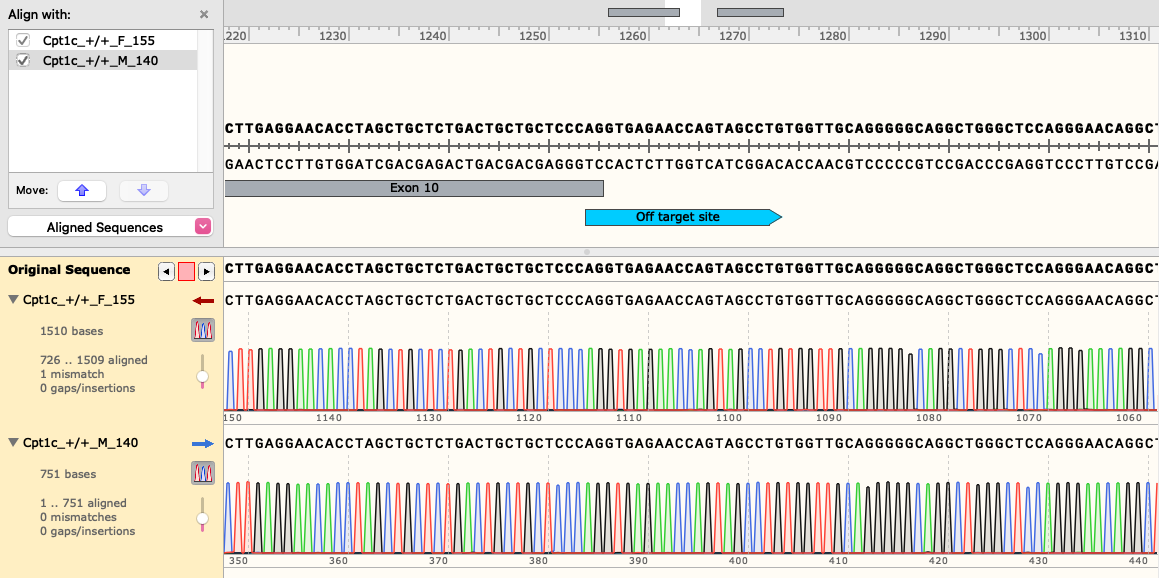

Supplement: Supplementary file 6 — Source Data [file 41467_2026_73227_MOESM6_ESM.zip › Source data/CRISPR off-targets sequencing/chromatograms/SEQ CRISPR off-target_Cpt1c_WT mouse.png]

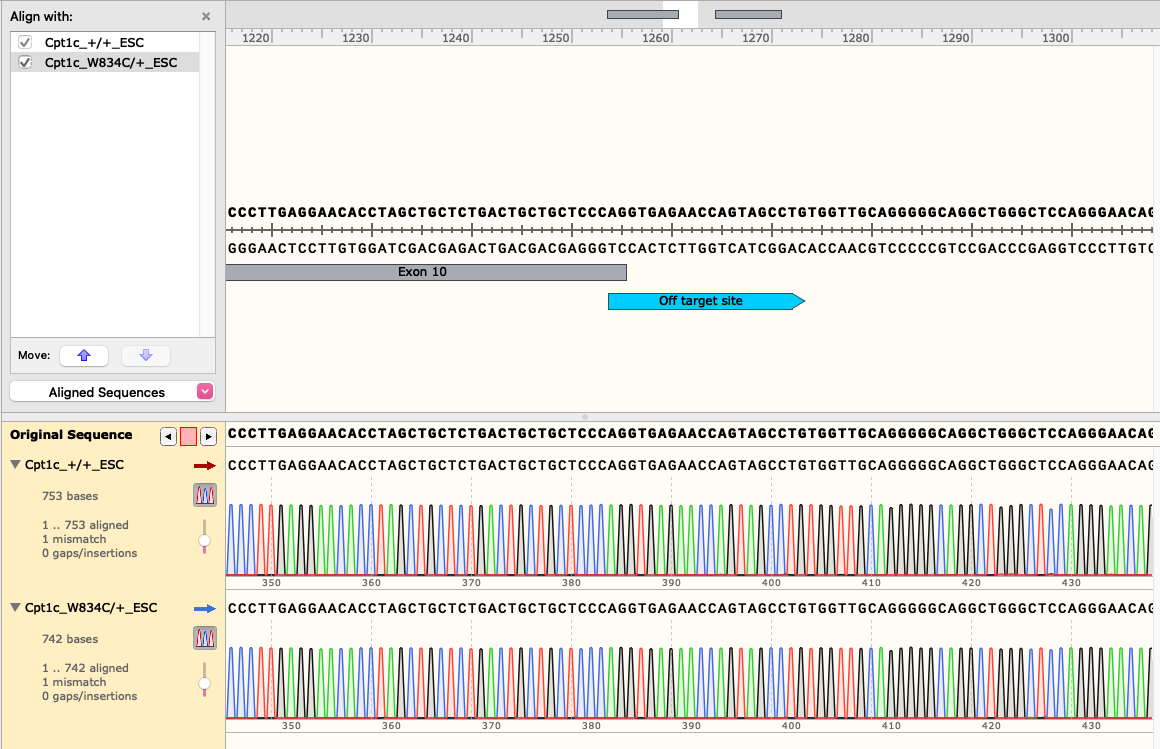

Supplement: Supplementary file 6 — Source Data [file 41467_2026_73227_MOESM6_ESM.zip › Source data/CRISPR off-targets sequencing/chromatograms/SEQ CRISPR off-target_Cpt1c_ESCs.png]

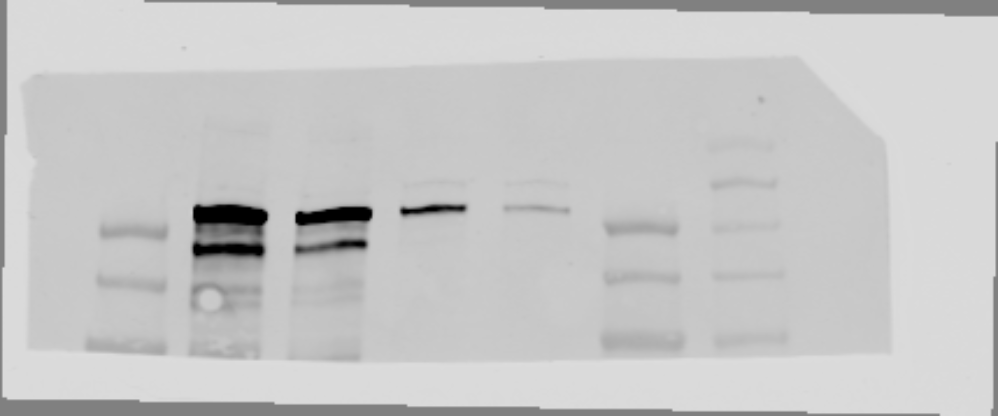

Supplement: Supplementary file 6 — Source Data [file 41467_2026_73227_MOESM6_ESM.zip › Source data/Western blots - uncropped images and replicates/Figure 1g/mCherry-SETD5 IPs in TBLR1 WT and KO cells + EGFP-ANKRD11_antiGFP.png]

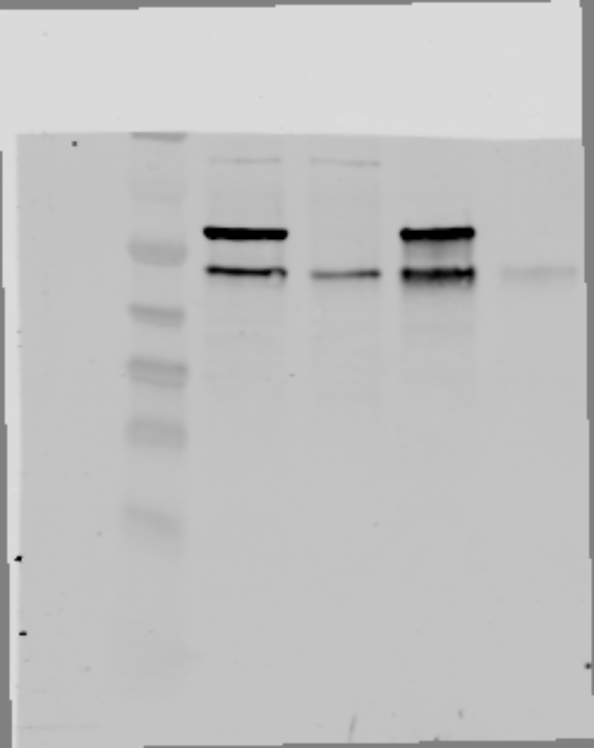

Supplement: Supplementary file 6 — Source Data [file 41467_2026_73227_MOESM6_ESM.zip › Source data/Western blots - uncropped images and replicates/Figure 1g/EGFP-ANKRD11 IPs in TBLR1 WT and KO cells + mCherry-SETD5_reprobe_antiHDAC3.png]

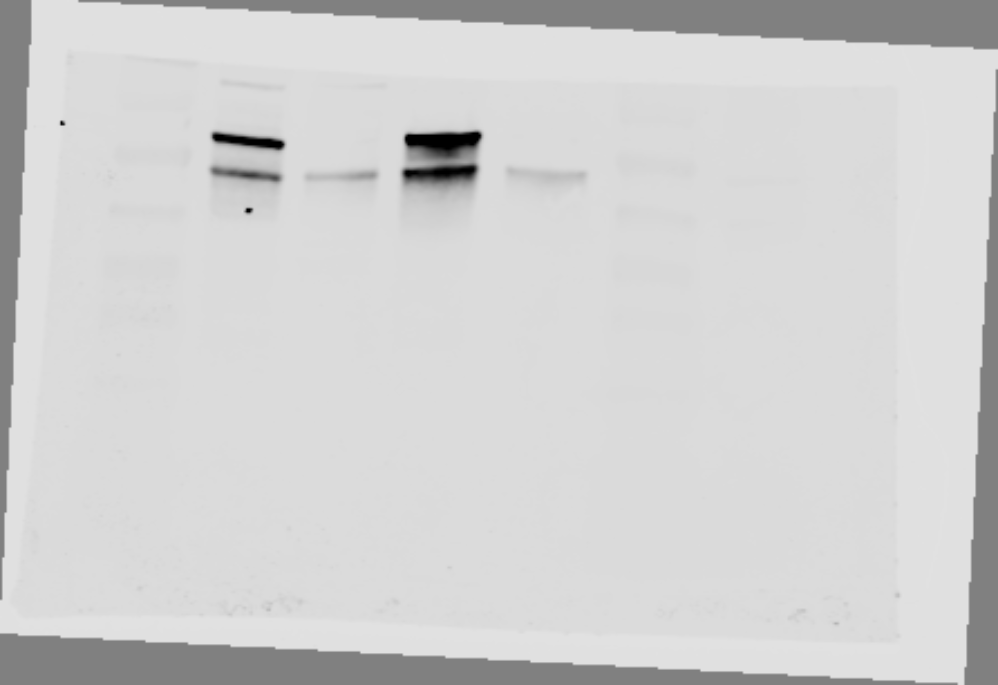

Supplement: Supplementary file 6 — Source Data [file 41467_2026_73227_MOESM6_ESM.zip › Source data/Western blots - uncropped images and replicates/Figure 1g/mCherry-SETD5 IPs in TBLR1 WT and KO cells + EGFP-ANKRD11_antiTBLR1_antiHDAC3.png]

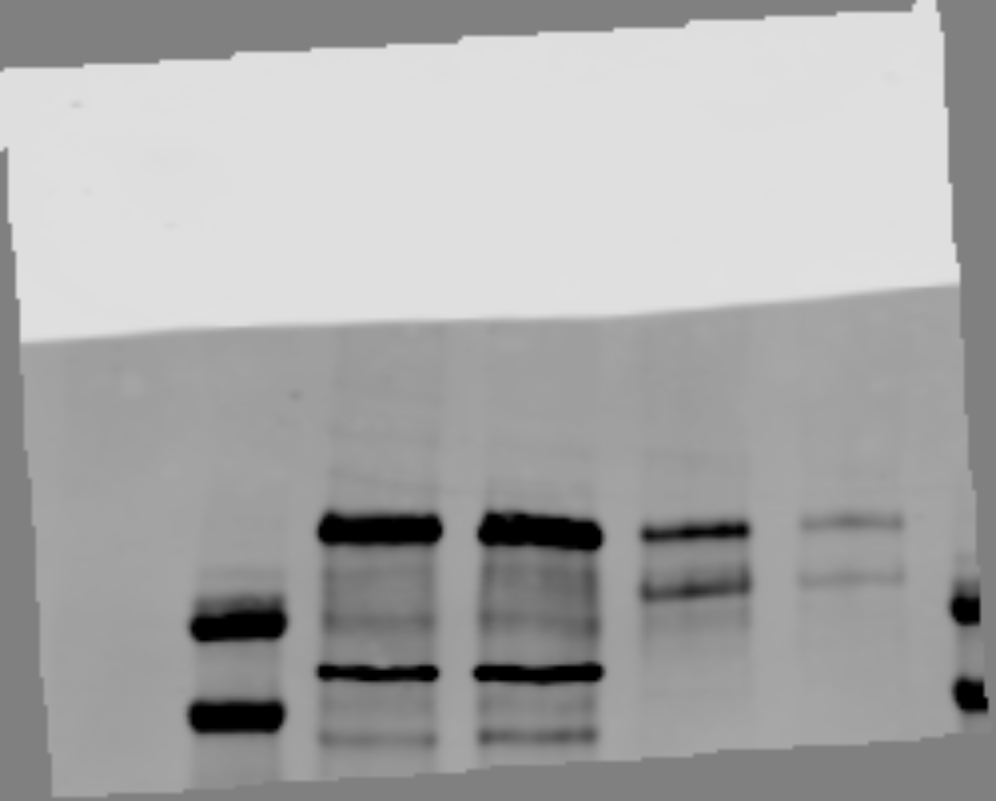

Supplement: Supplementary file 6 — Source Data [file 41467_2026_73227_MOESM6_ESM.zip › Source data/Western blots - uncropped images and replicates/Figure 1g/EGFP-ANKRD11 IPs in TBLR1 WT and KO cells + mCherry-Setd5_antimCherry.png]

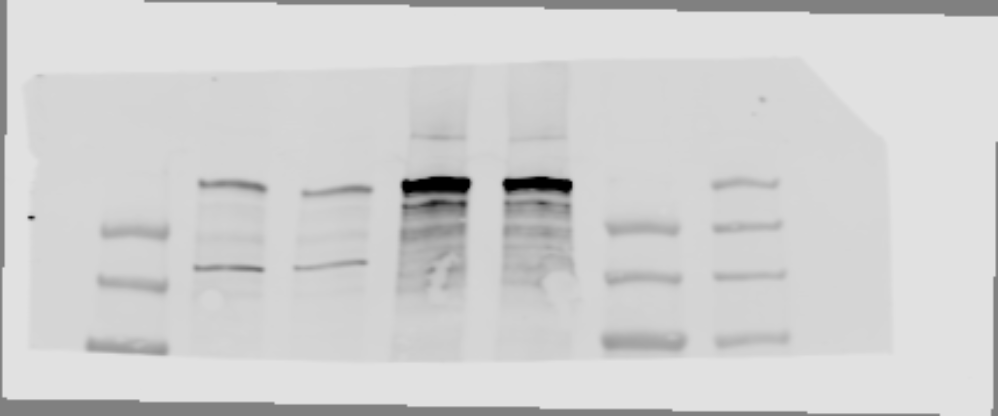

Supplement: Supplementary file 6 — Source Data [file 41467_2026_73227_MOESM6_ESM.zip › Source data/Western blots - uncropped images and replicates/Figure 1g/mCherry-SETD5 IPs in TBLR1 WT and KO cells + EGFP-ANKRD11_antimCherry.png]

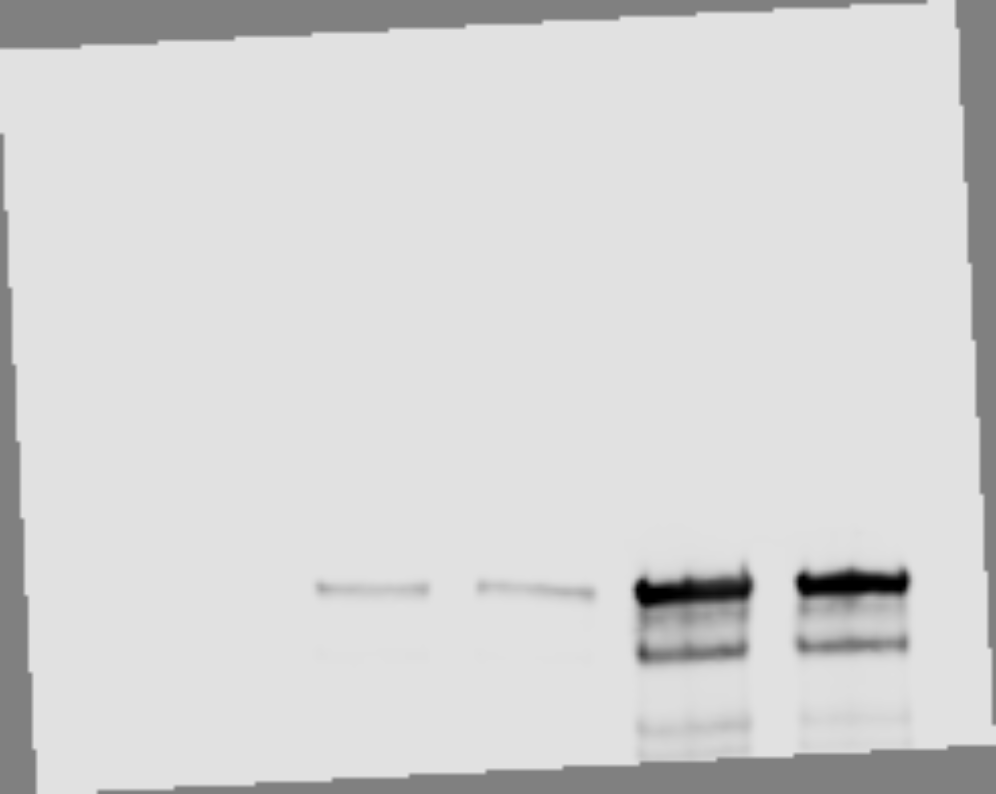

Supplement: Supplementary file 6 — Source Data [file 41467_2026_73227_MOESM6_ESM.zip › Source data/Western blots - uncropped images and replicates/Figure 1g/EGFP-ANKRD11 IPs in TBLR1 WT and KO cells + mCherry-Setd5_antiGFP.png]

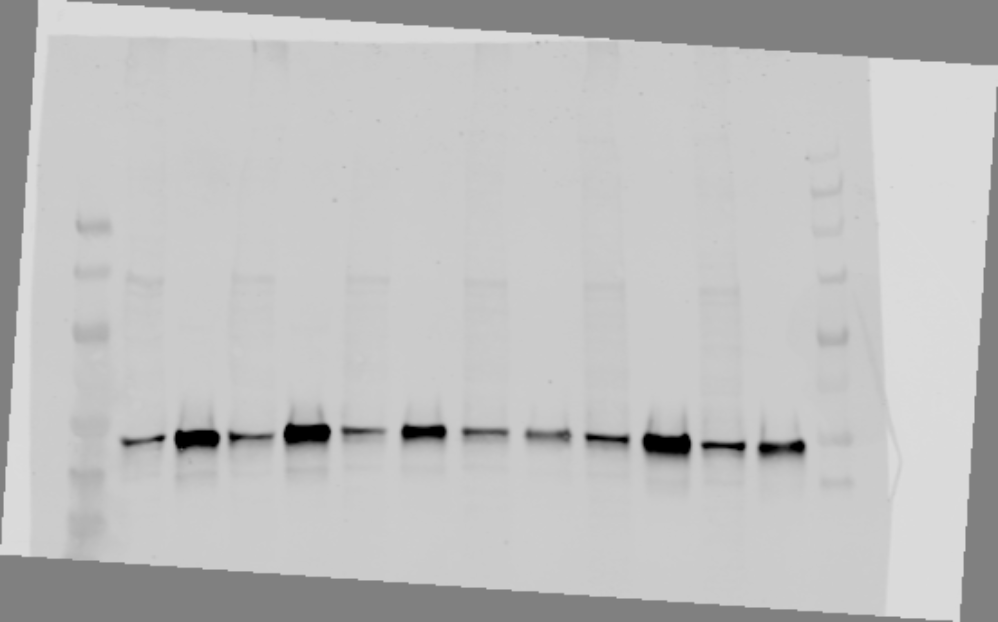

Supplement: Supplementary file 6 — Source Data [file 41467_2026_73227_MOESM6_ESM.zip › Source data/Western blots - uncropped images and replicates/Supplementary Figure 1c/mCherry-TBLR1 N-term mutants IPs in FLAG-TBLR1 transfected cells_antiHDAC3.png]

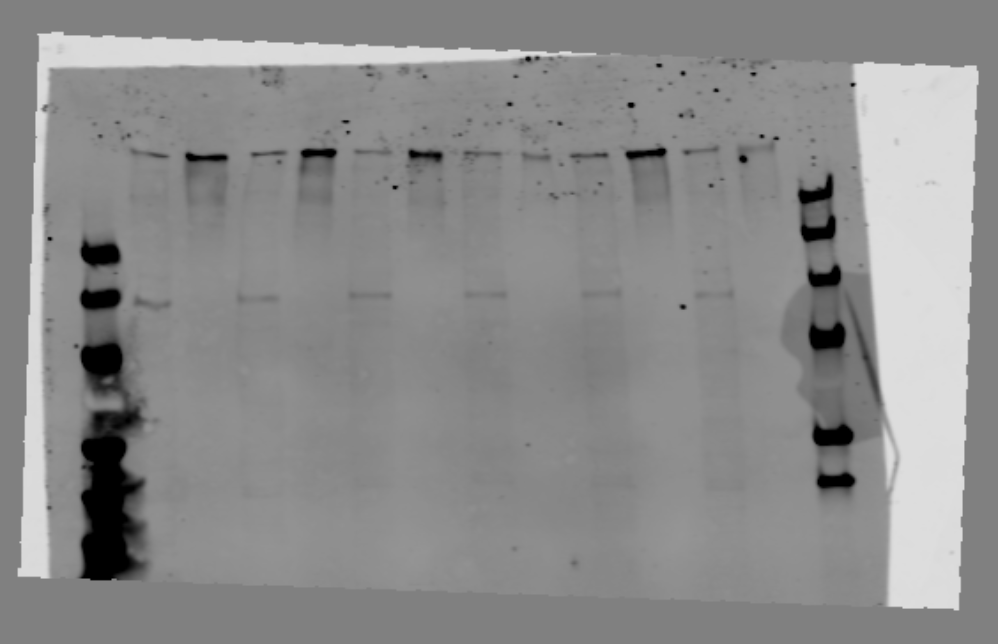

Supplement: Supplementary file 6 — Source Data [file 41467_2026_73227_MOESM6_ESM.zip › Source data/Western blots - uncropped images and replicates/Supplementary Figure 1c/mCherry-TBLR1 N-term mutants IPs in FLAG-TBLR1 transfected cells_antiNCOR1.png]

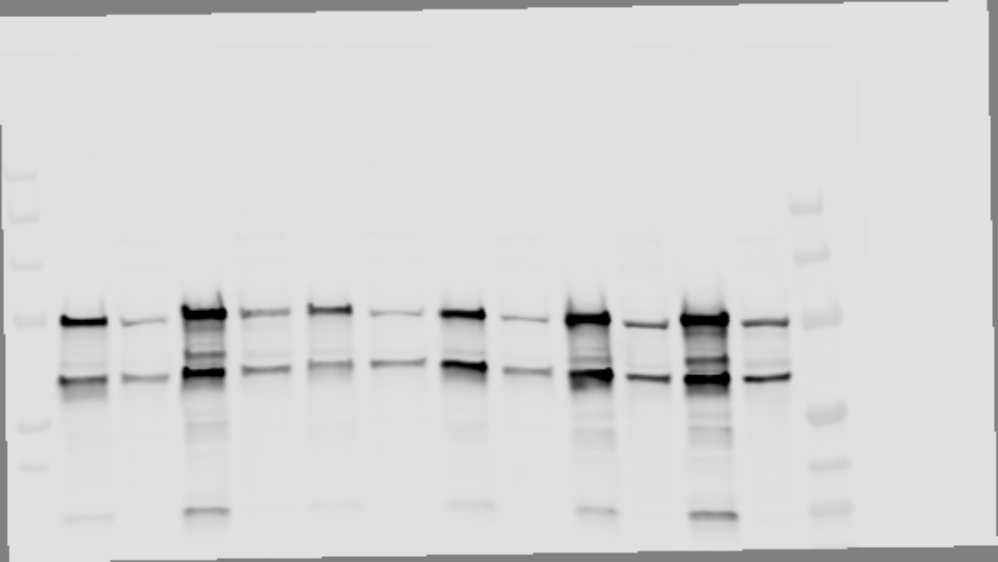

Supplement: Supplementary file 6 — Source Data [file 41467_2026_73227_MOESM6_ESM.zip › Source data/Western blots - uncropped images and replicates/Supplementary Figure 1c/mCherry-TBLR1 N-term mutants IPs in FLAG-TBLR1 transfected cells_antiFLAG_antimCherry.png]

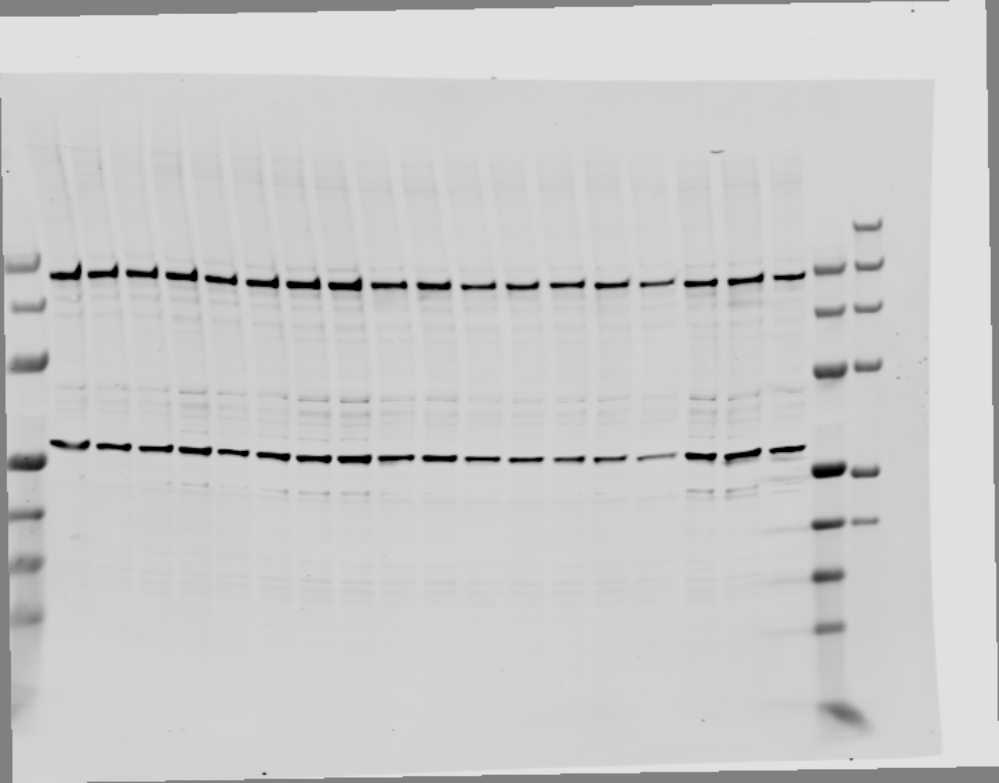

Supplement: Supplementary file 6 — Source Data [file 41467_2026_73227_MOESM6_ESM.zip › Source data/Western blots - uncropped images and replicates/Supplementary Figure 1d/Protein extracts TBLR1 mutant TREx cells_A311P-D369E_antiSIN3A_antiTBLR1.png]

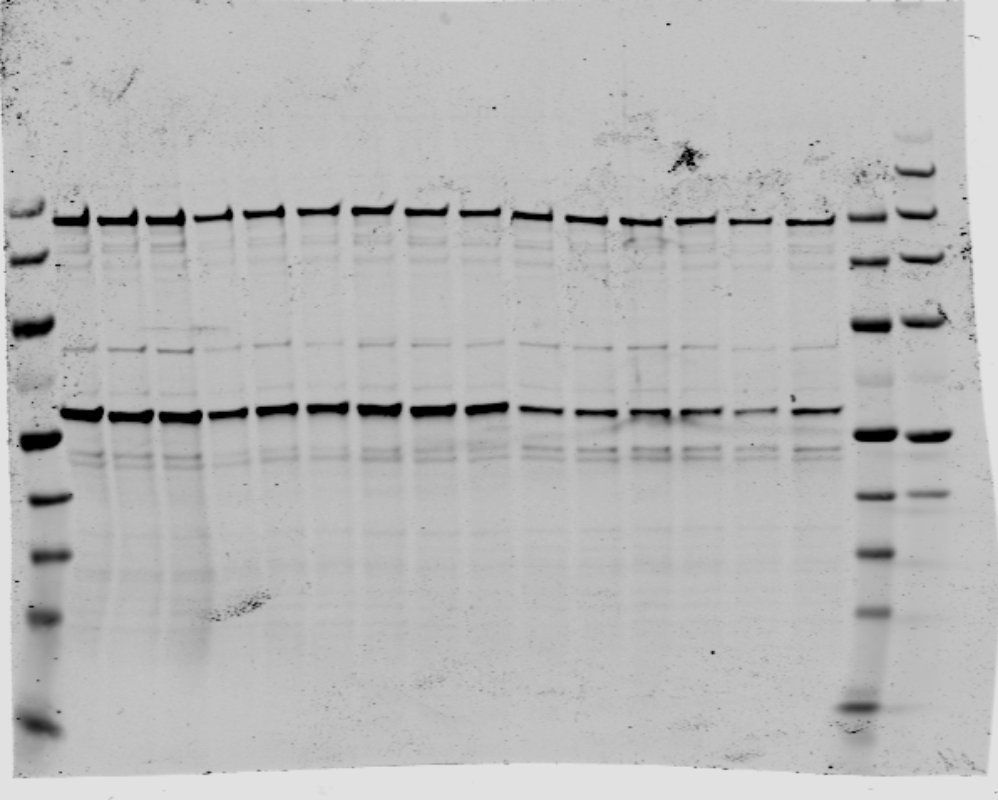

Supplement: Supplementary file 6 — Source Data [file 41467_2026_73227_MOESM6_ESM.zip › Source data/Western blots - uncropped images and replicates/Supplementary Figure 1d/Protein extracts TBLR1 mutant TREx cells_Y446C-G460D_antiTBLR1_antiSin3a.png]

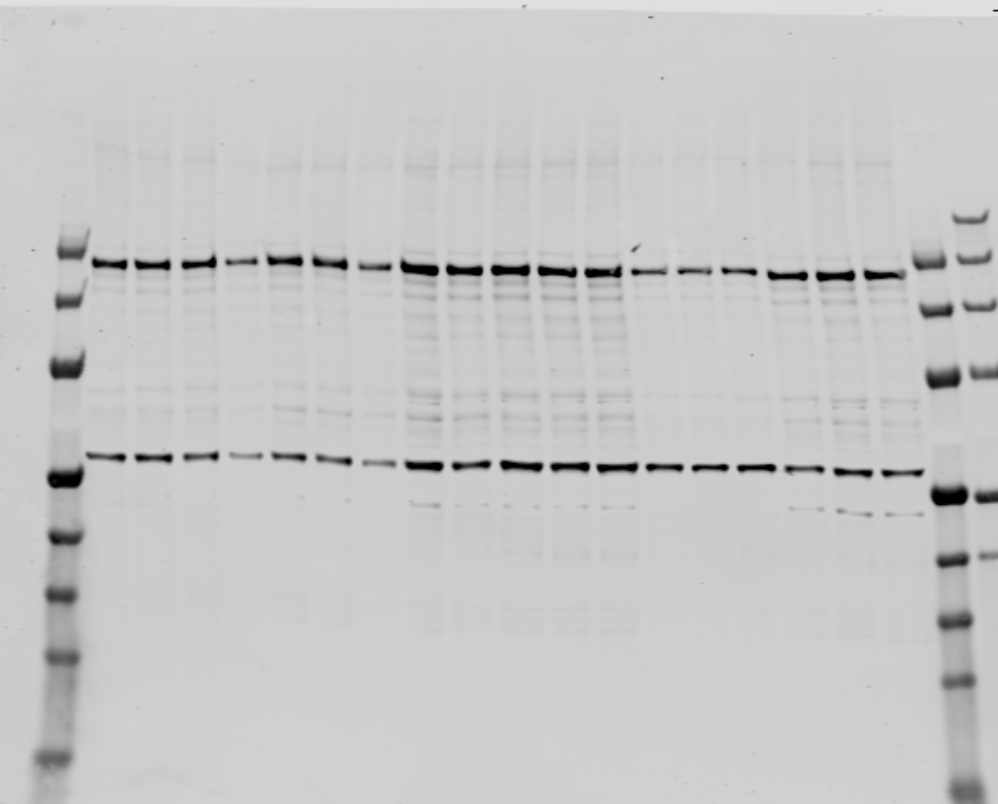

Supplement: Supplementary file 6 — Source Data [file 41467_2026_73227_MOESM6_ESM.zip › Source data/Western blots - uncropped images and replicates/Supplementary Figure 1d/Protein extracts TBLR1 mutant TREx cells_G70D-L282P_antiSIN3A_antiTBLR1.png]

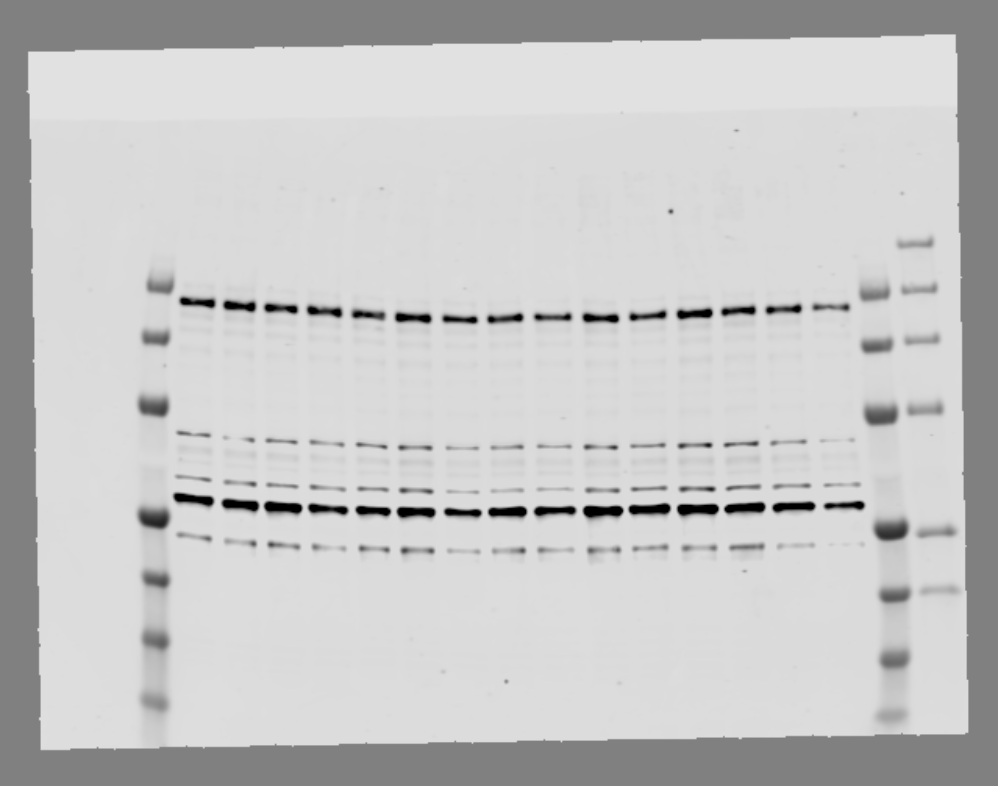

Supplement: Supplementary file 6 — Source Data [file 41467_2026_73227_MOESM6_ESM.zip › Source data/Western blots - uncropped images and replicates/Supplementary Figure 1d/Protein extracts TBLR1 mutant TREx cells_F10L-L83Q_antiSIN3A_antiTBLR1.png]

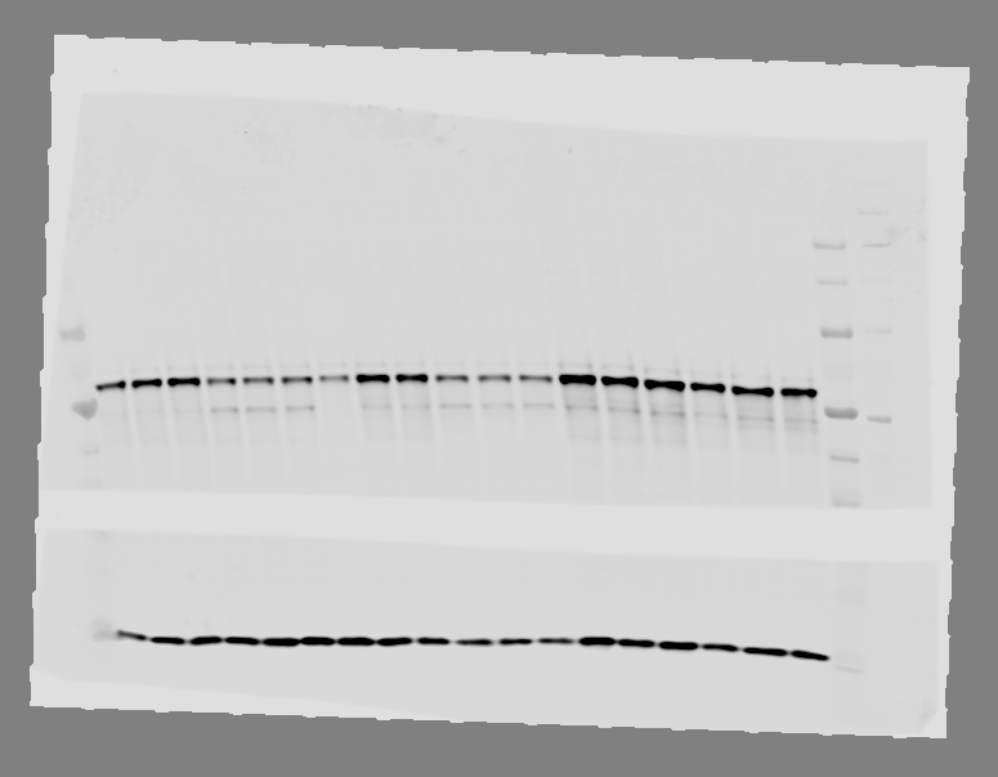

Supplement: Supplementary file 6 — Source Data [file 41467_2026_73227_MOESM6_ESM.zip › Source data/Western blots - uncropped images and replicates/Supplementary Figure 1d/Protein extracts TBLR1 mutant TREx cells_D370N-P444R_antiTBLR1_antiH3.png]

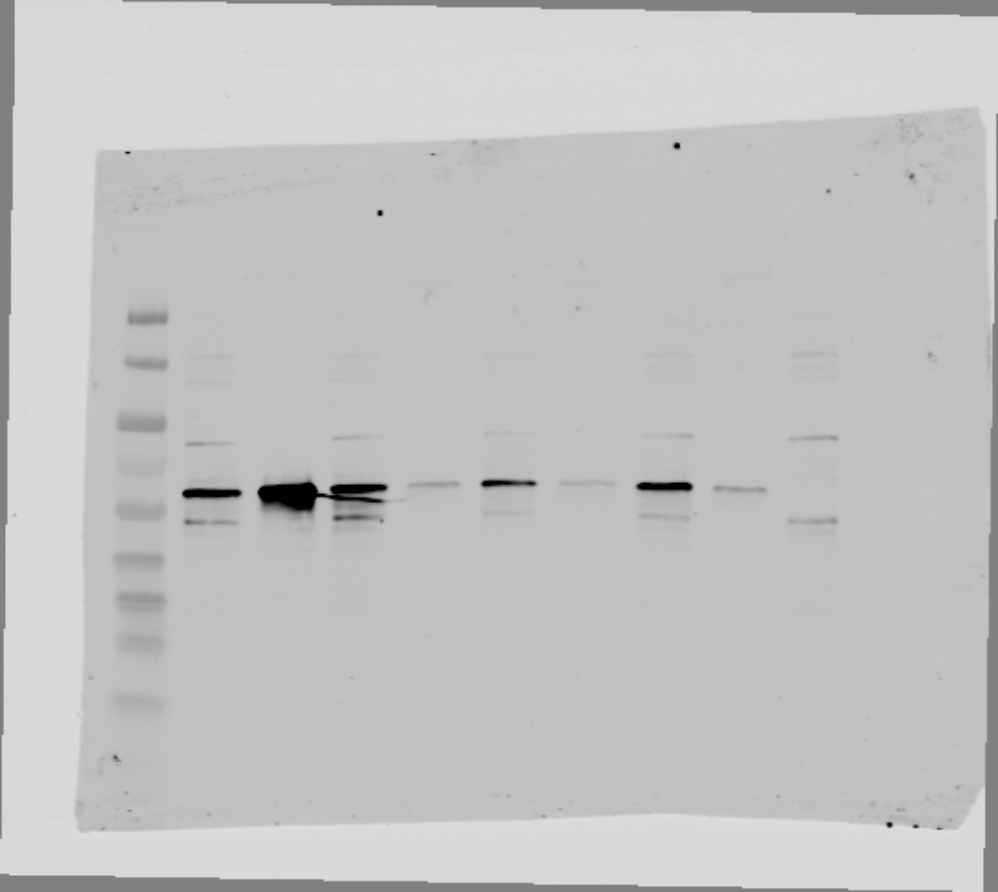

Supplement: Supplementary file 6 — Source Data [file 41467_2026_73227_MOESM6_ESM.zip › Source data/Western blots - uncropped images and replicates/Supplementary Figure 5b/mCherry-SETD5 K and KWK NID mutants IPs_antiTBLR1.png]

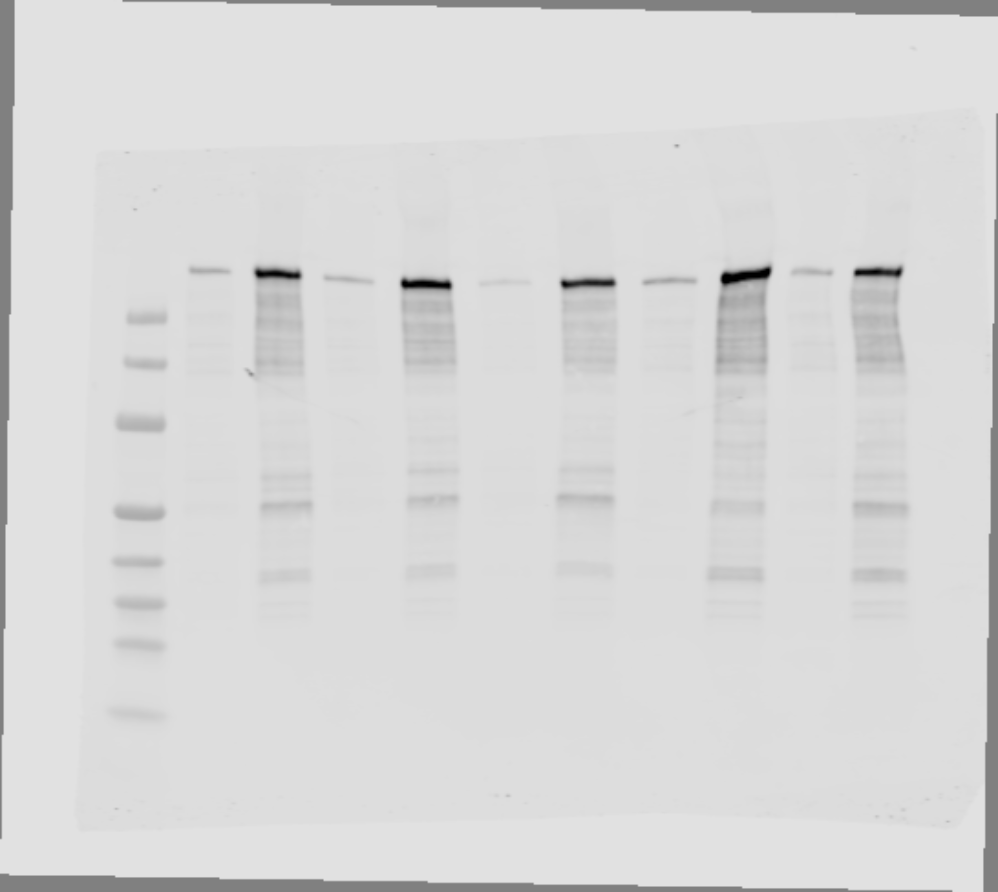

Supplement: Supplementary file 6 — Source Data [file 41467_2026_73227_MOESM6_ESM.zip › Source data/Western blots - uncropped images and replicates/Supplementary Figure 5b/mCherry-SETD5 K and KWK NID mutants IPs_antimCherry.png]

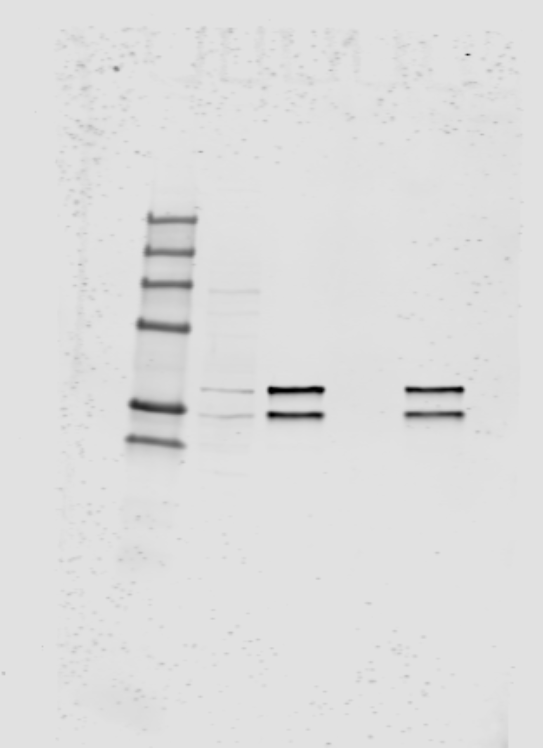

Supplement: Supplementary file 6 — Source Data [file 41467_2026_73227_MOESM6_ESM.zip › Source data/Western blots - uncropped images and replicates/Figure 3e/MeCP2 SETD5 NID peptide pulldown EGFP-ANKRD11(Cterm) transfected cells_antiTBLR1_antiHDAC3.png]

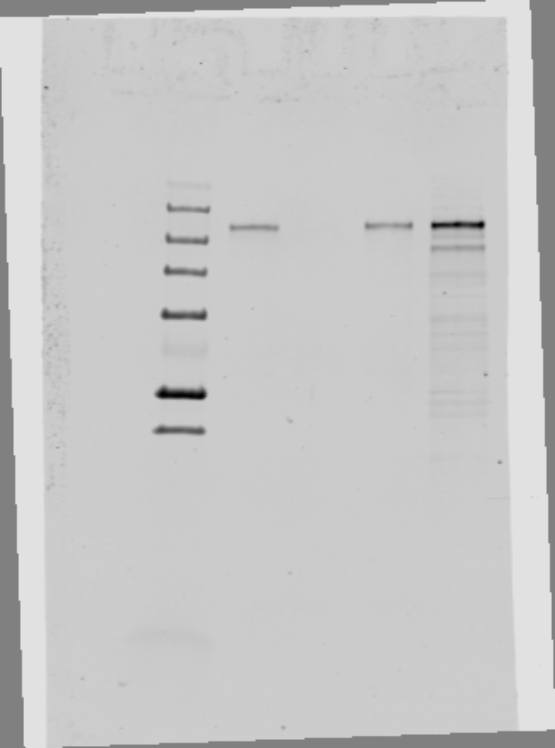

Supplement: Supplementary file 6 — Source Data [file 41467_2026_73227_MOESM6_ESM.zip › Source data/Western blots - uncropped images and replicates/Figure 3e/MeCP2 SETD5 NID peptide pulldown EGFP-ANKRD11(Cterm) transfected cells_antiGFP.png]

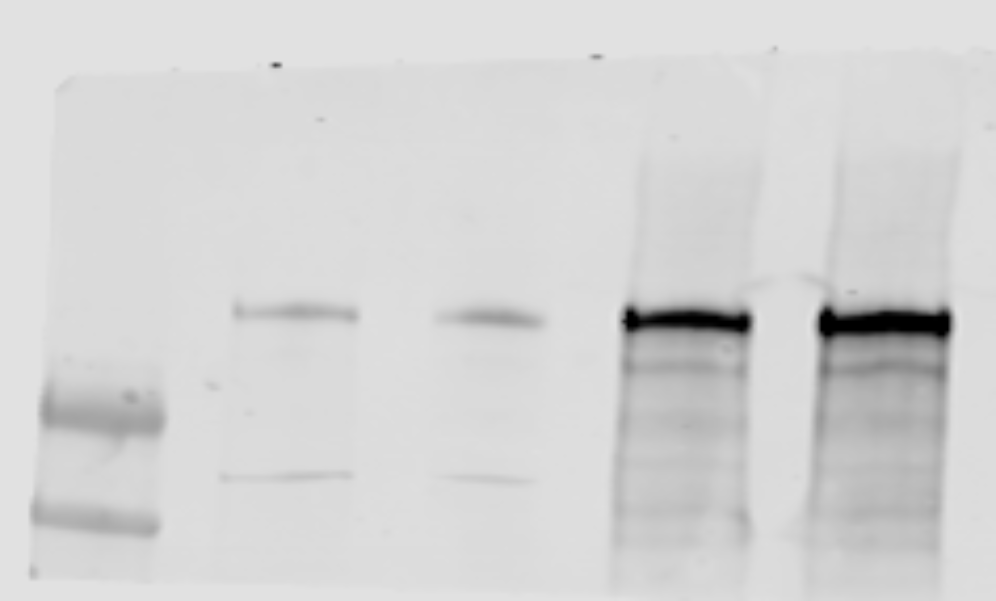

Supplement: Supplementary file 6 — Source Data [file 41467_2026_73227_MOESM6_ESM.zip › Source data/Western blots - uncropped images and replicates/Figure 3d/mCherry-SETD5 IPs in GFP-ANKRD11(C-term) transfected cells_gel 2_antimcherry.png]

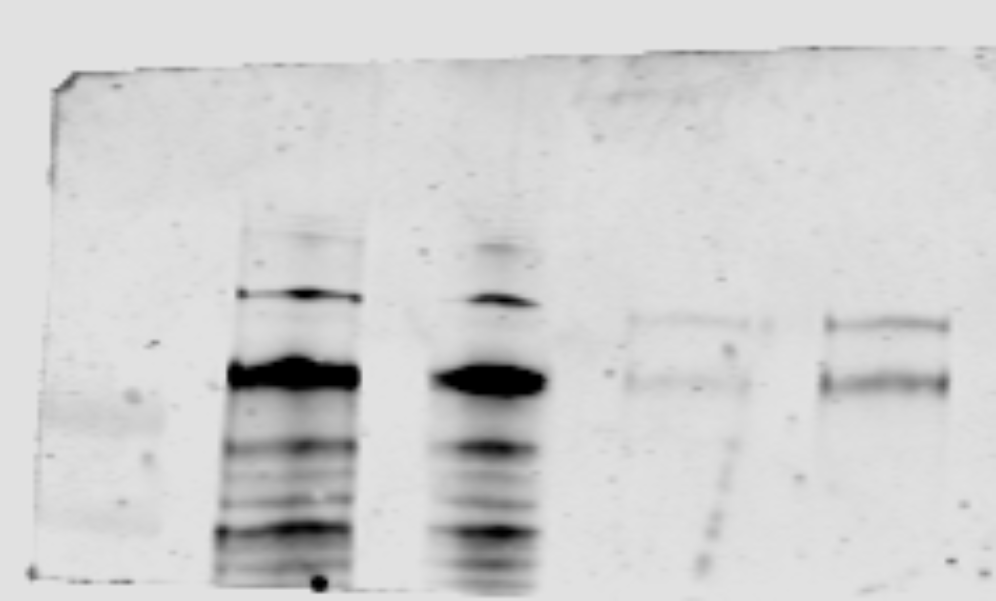

Supplement: Supplementary file 6 — Source Data [file 41467_2026_73227_MOESM6_ESM.zip › Source data/Western blots - uncropped images and replicates/Figure 3d/mCherry-SETD5 IPs in GFP-ANKRD11(C-term) transfected cells_gel 2_antiGFP.png]

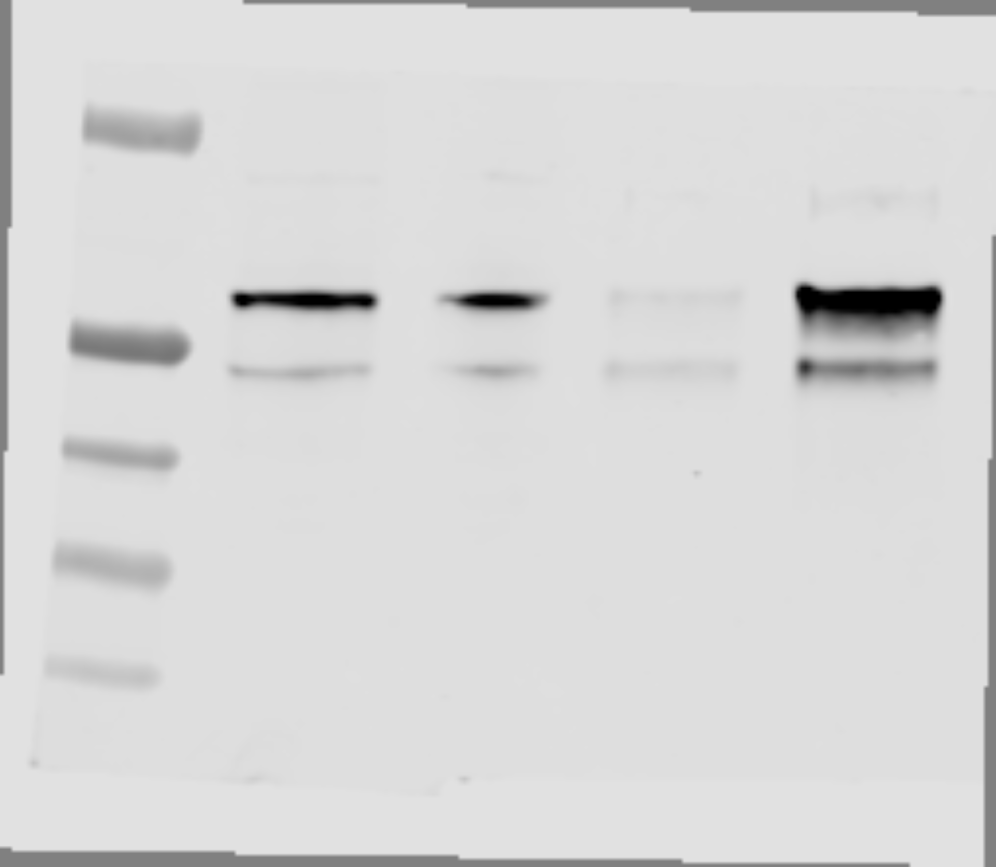

Supplement: Supplementary file 6 — Source Data [file 41467_2026_73227_MOESM6_ESM.zip › Source data/Western blots - uncropped images and replicates/Figure 3d/mCherry-SETD5 IPs in GFP-ANKRD11(C-term) transfected cells_gel 2_antiTBLR1.png]

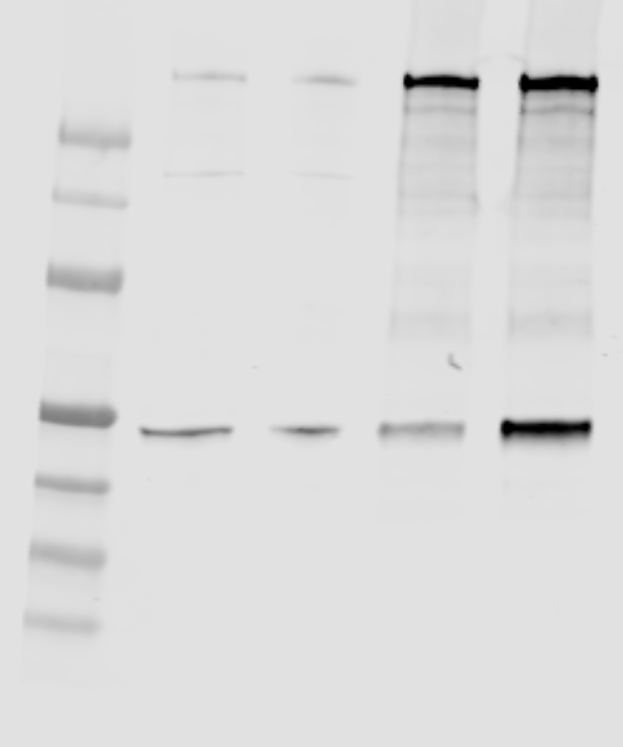

Supplement: Supplementary file 6 — Source Data [file 41467_2026_73227_MOESM6_ESM.zip › Source data/Western blots - uncropped images and replicates/Figure 3d/mCherry-SETD5 IPs in GFP-ANKRD11(C-term) transfected cells_gel 1_antimCherry_antiHDAC3.png]

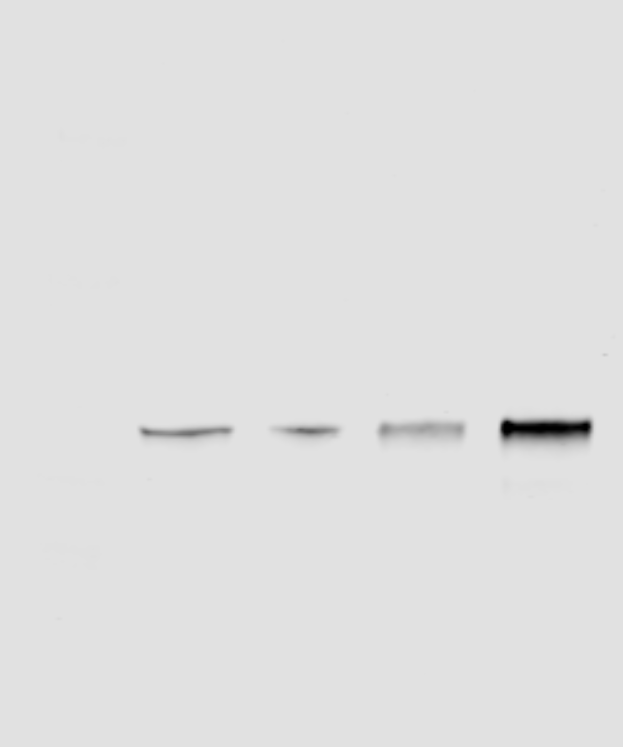

Supplement: Supplementary file 6 — Source Data [file 41467_2026_73227_MOESM6_ESM.zip › Source data/Western blots - uncropped images and replicates/Figure 3d/mCherry-SETD5 IPs in GFP-ANKRD11(C-term) transfected cells_gel 1_antiHDAC3.png]

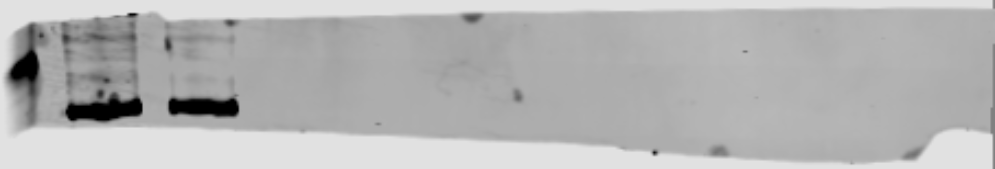

Supplement: Supplementary file 6 — Source Data [file 41467_2026_73227_MOESM6_ESM.zip › Source data/Western blots - uncropped images and replicates/Figure 5a/competition assay EGFP-MeCP2 IP from EGFP-MeCP2 + mCherry-SETD5 transfected extracts_antimCherry.png]

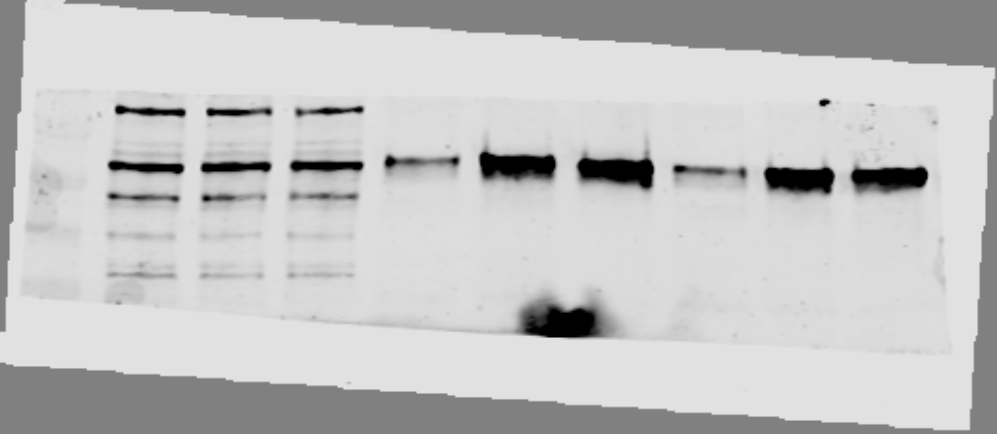

Supplement: Supplementary file 6 — Source Data [file 41467_2026_73227_MOESM6_ESM.zip › Source data/Western blots - uncropped images and replicates/Figure 5a/competition assay EGFP-MeCP2 IP from EGFP-MeCP2 + mCherry-SETD5 transfected extracts_antiTBLR1.png]

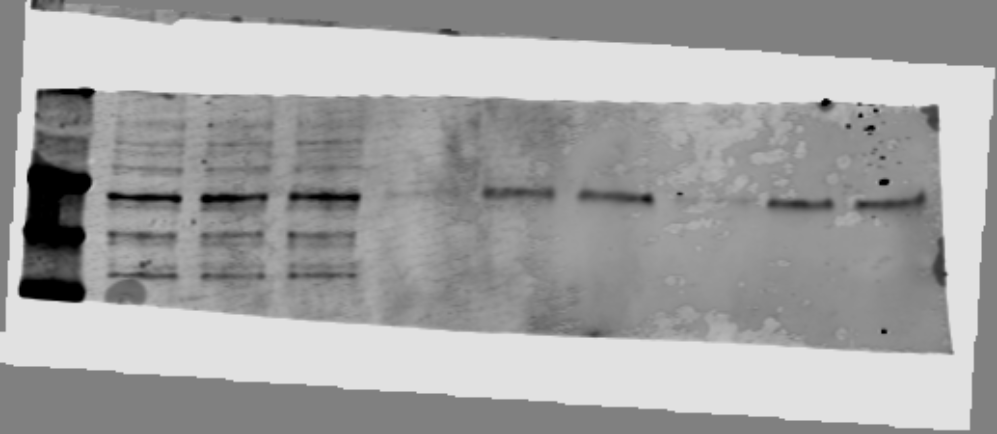

Supplement: Supplementary file 6 — Source Data [file 41467_2026_73227_MOESM6_ESM.zip › Source data/Western blots - uncropped images and replicates/Figure 5a/competition assay EGFP-MeCP2 IP from EGFP-MeCP2 + mCherry-SETD5 transfected extracts_antiHDAC3.png]

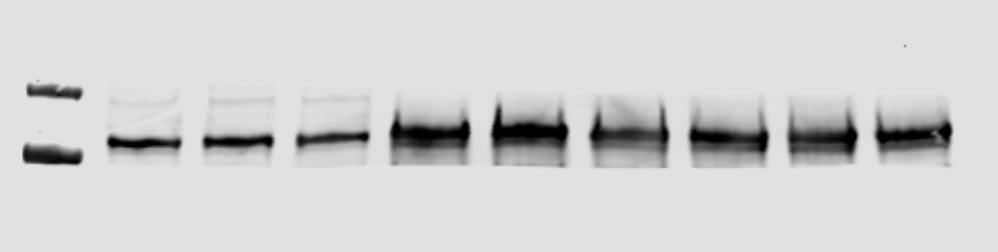

Supplement: Supplementary file 6 — Source Data [file 41467_2026_73227_MOESM6_ESM.zip › Source data/Western blots - uncropped images and replicates/Figure 5a/competition assay EGFP-MeCP2 IP from EGFP-MeCP2 + mCherry-SETD5 transfected extracts_antiGFP.png]

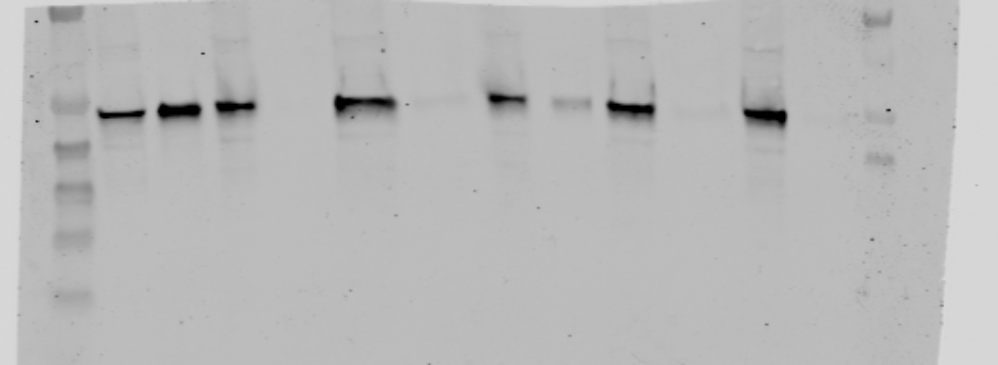

Supplement: Supplementary file 6 — Source Data [file 41467_2026_73227_MOESM6_ESM.zip › Source data/Western blots - uncropped images and replicates/Figure 2d/EGFP-ANKRD11-full length NID mutants_IPs_antiHDAC3.png]

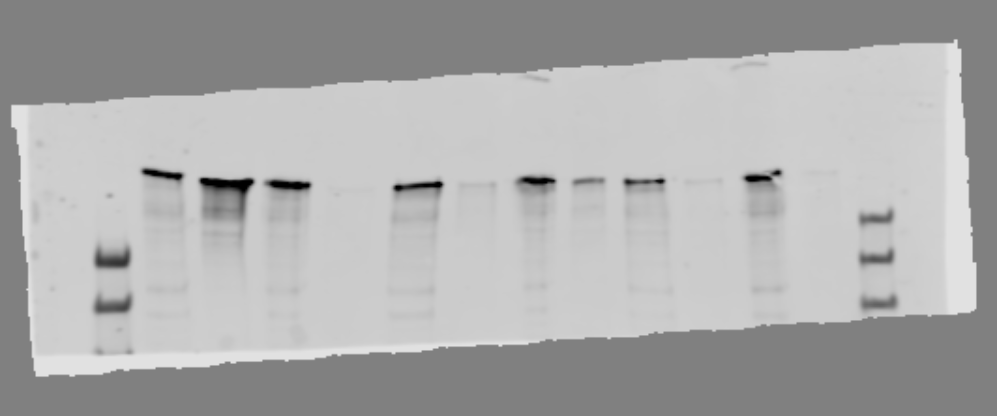

Supplement: Supplementary file 6 — Source Data [file 41467_2026_73227_MOESM6_ESM.zip › Source data/Western blots - uncropped images and replicates/Figure 2d/EGFP-ANKRD11-full length NID mutants_IPs_antiNCOR1.png]

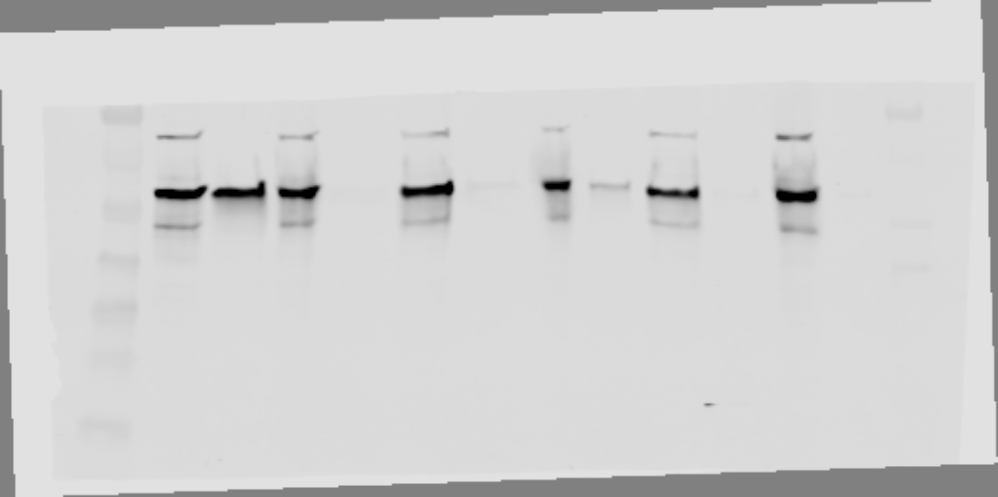

Supplement: Supplementary file 6 — Source Data [file 41467_2026_73227_MOESM6_ESM.zip › Source data/Western blots - uncropped images and replicates/Figure 2d/EGFP-ANKRD11-full length NID mutants_IPs_antiTBLR1.png]

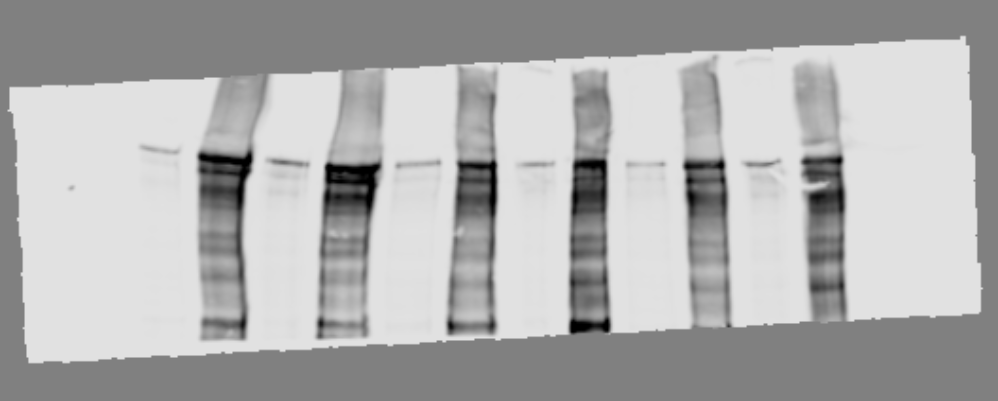

Supplement: Supplementary file 6 — Source Data [file 41467_2026_73227_MOESM6_ESM.zip › Source data/Western blots - uncropped images and replicates/Figure 2d/EGFP-ANKRD11-full length NID mutants IPs_antiGFP.png]

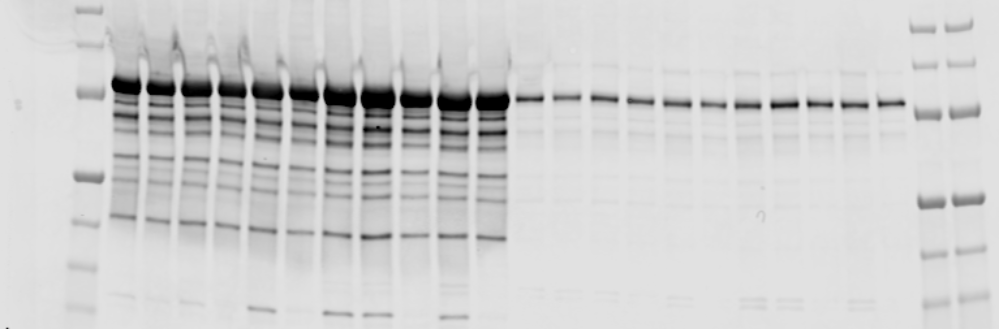

Supplement: Supplementary file 6 — Source Data [file 41467_2026_73227_MOESM6_ESM.zip › Source data/Western blots - uncropped images and replicates/Figure 1e/EGFP-MeCP2 IPs in TBLR1 mutants_G70D_D369E_antiGFP.png]

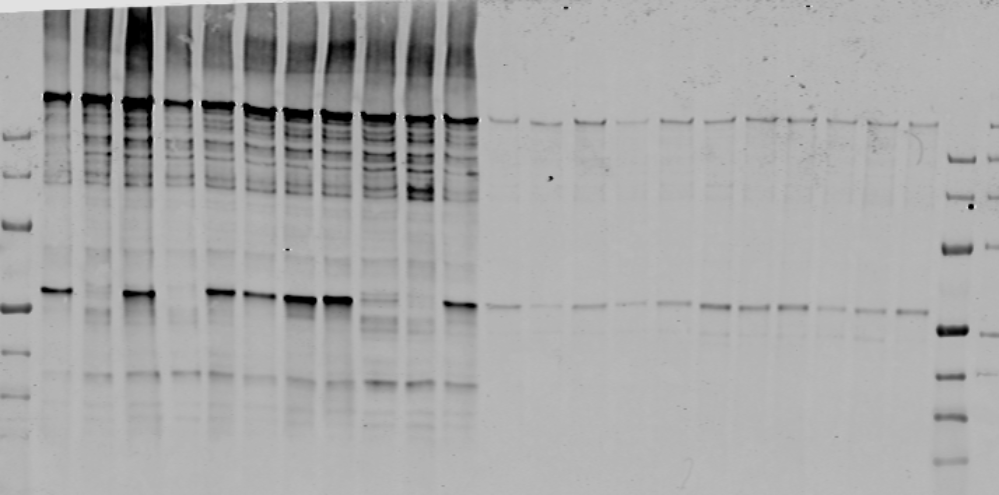

Supplement: Supplementary file 6 — Source Data [file 41467_2026_73227_MOESM6_ESM.zip › Source data/Western blots - uncropped images and replicates/Figure 1e/EGFP-SETD5 IPs in TBLR1 mutants D370N_G460D.png]

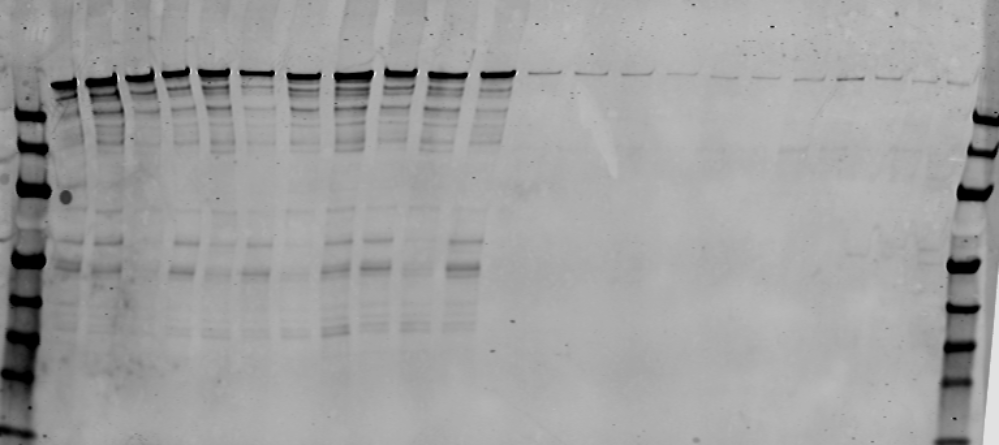

Supplement: Supplementary file 6 — Source Data [file 41467_2026_73227_MOESM6_ESM.zip › Source data/Western blots - uncropped images and replicates/Figure 1e/EGFP-SETD5 IPs in TBLR1 mutants G70D_D369E_antiGFP.png]

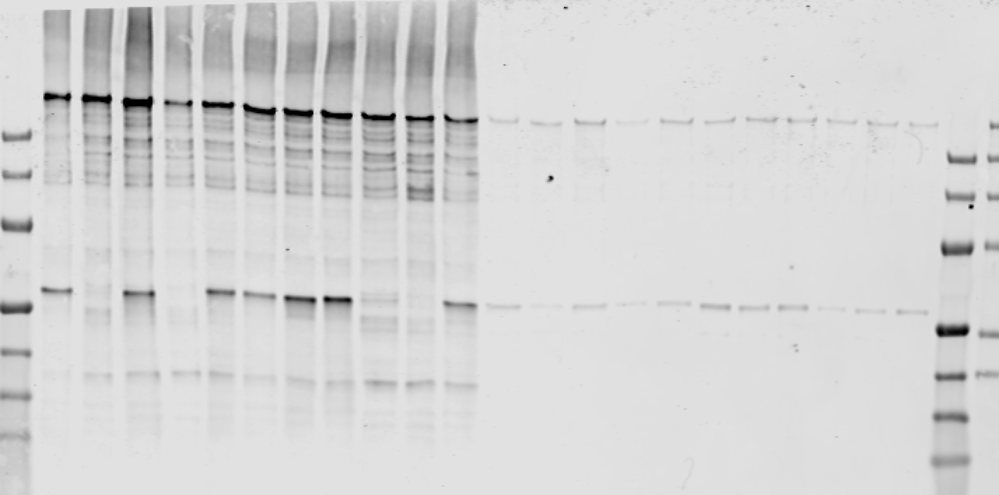

Supplement: Supplementary file 6 — Source Data [file 41467_2026_73227_MOESM6_ESM.zip › Source data/Western blots - uncropped images and replicates/Figure 1e/EGFP-SETD5 IPs in TBLR1 mutants D370N_G460D_reduced intensity.png]

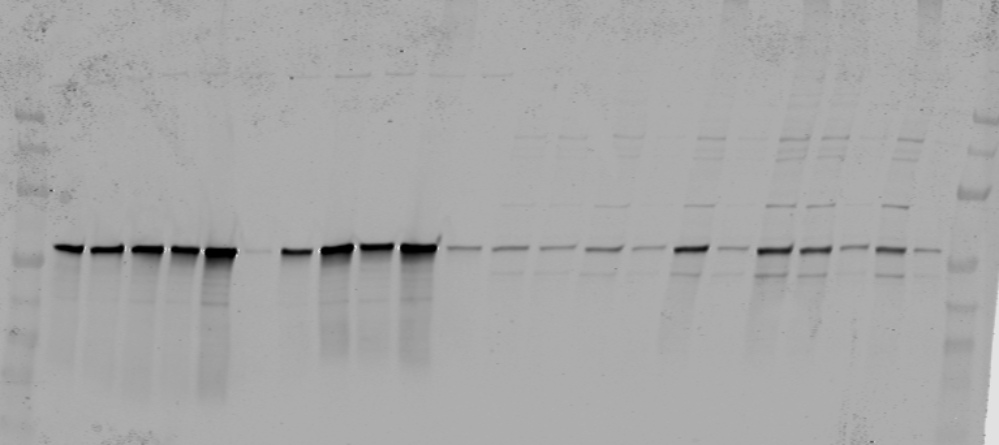

Supplement: Supplementary file 6 — Source Data [file 41467_2026_73227_MOESM6_ESM.zip › Source data/Western blots - uncropped images and replicates/Figure 1e/EGFP-SETD5 IPs in TBLR1 mutants G70D_D369E_antiTBLR1.png]

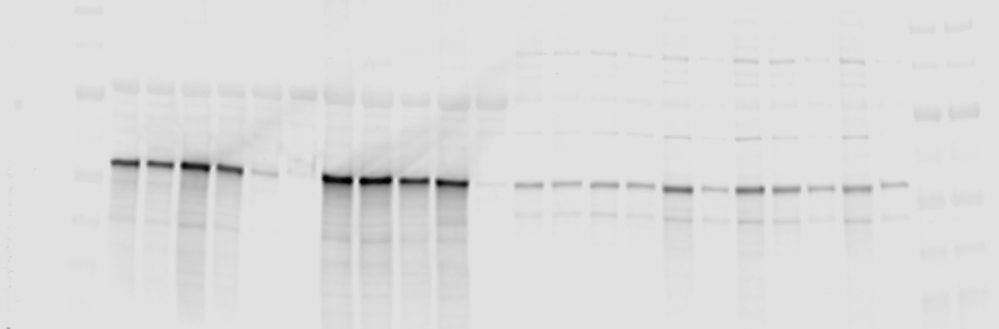

Supplement: Supplementary file 6 — Source Data [file 41467_2026_73227_MOESM6_ESM.zip › Source data/Western blots - uncropped images and replicates/Figure 1e/EGFP-MeCP2 IPs in TBLR1 mutants_G70D_D369E_antiTBLR1.png]

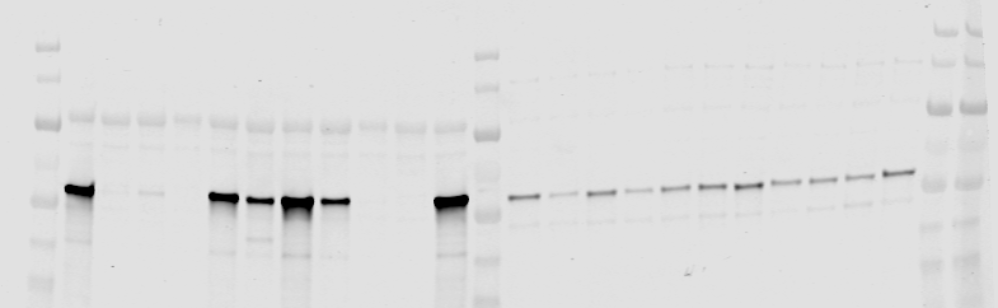

Supplement: Supplementary file 6 — Source Data [file 41467_2026_73227_MOESM6_ESM.zip › Source data/Western blots - uncropped images and replicates/Figure 1e/EGFP-MeCP2 IPs in TBLR1 mutants_D370N_G460D_antiTBLR1.png]

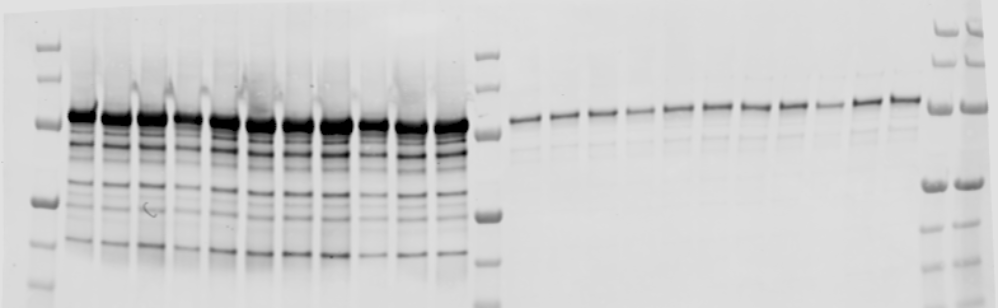

Supplement: Supplementary file 6 — Source Data [file 41467_2026_73227_MOESM6_ESM.zip › Source data/Western blots - uncropped images and replicates/Figure 1e/EGFP-MeCP2 IPs in TBLR1 mutants_D370N_G460D_antiGFP.png]

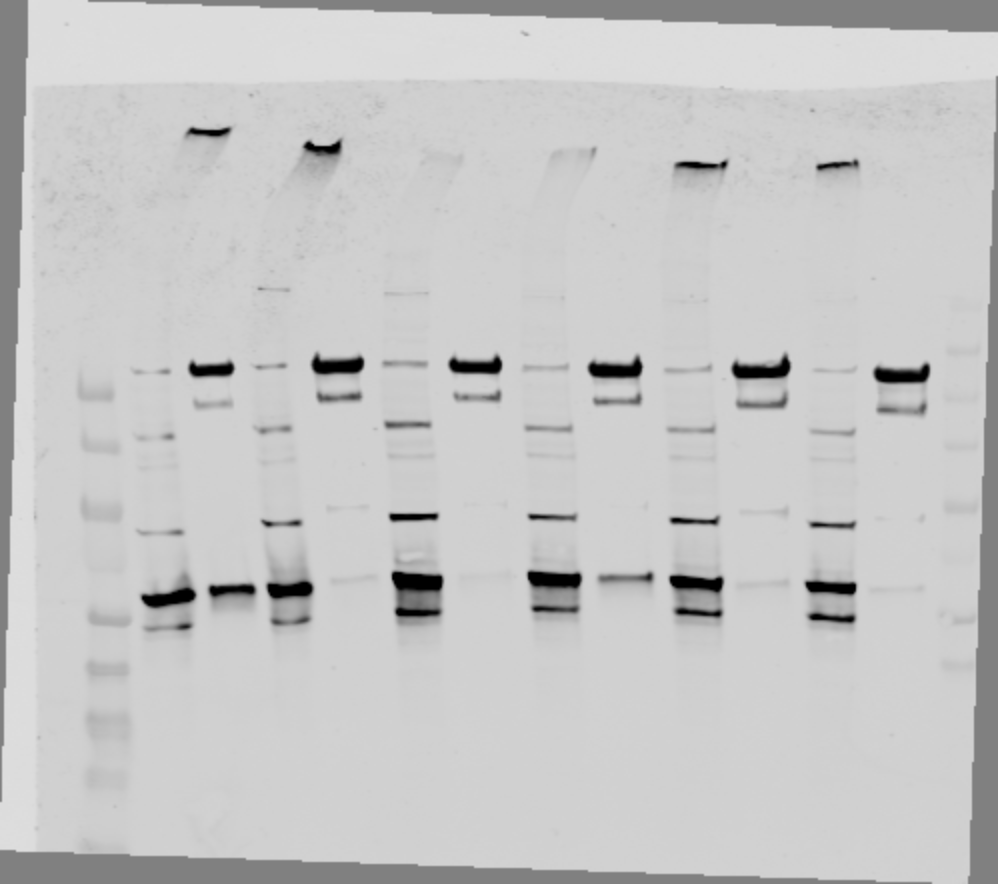

Supplement: Supplementary file 6 — Source Data [file 41467_2026_73227_MOESM6_ESM.zip › Source data/Western blots - uncropped images and replicates/Figure 1c/EGFP-ANKRD11 IPs in TBLR1 N-term mutants_F10L-L83Q_antiGFP_antiTBLR1.png]

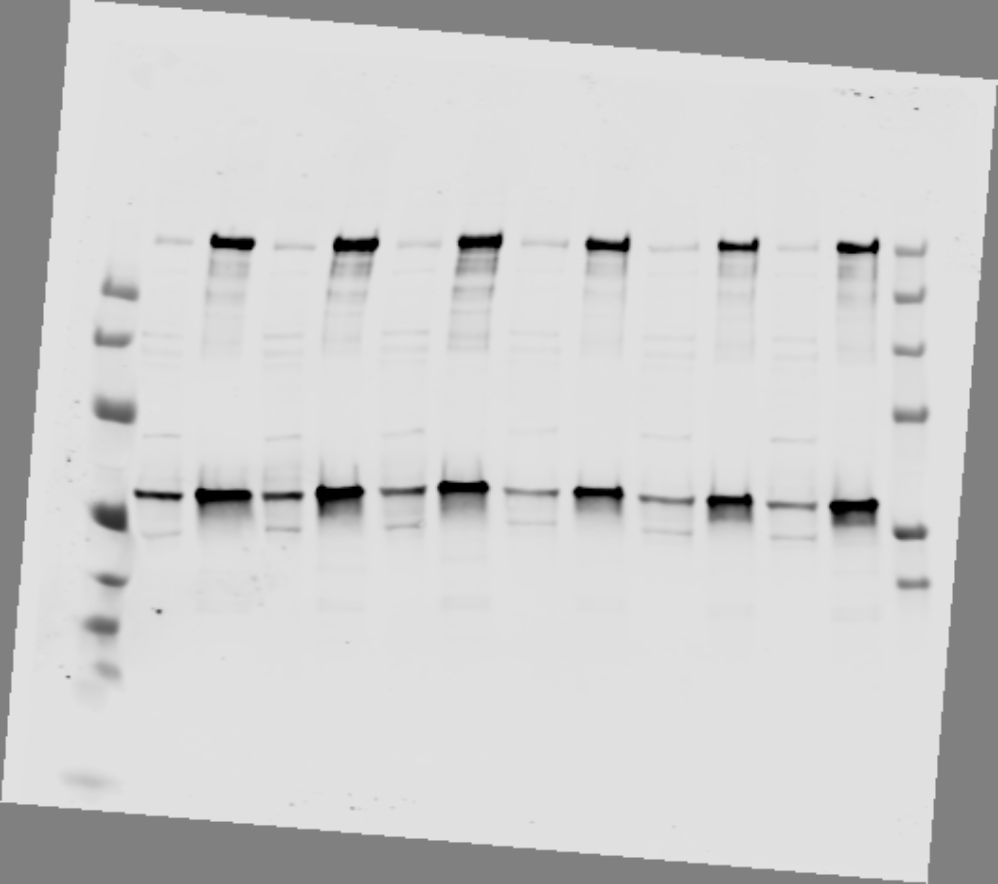

Supplement: Supplementary file 6 — Source Data [file 41467_2026_73227_MOESM6_ESM.zip › Source data/Western blots - uncropped images and replicates/Figure 1c/EGFP-SETD5 IPs in TBLR1 N-term mutants_F10L-L83Q_antiGFP_antiTBLR1.png]

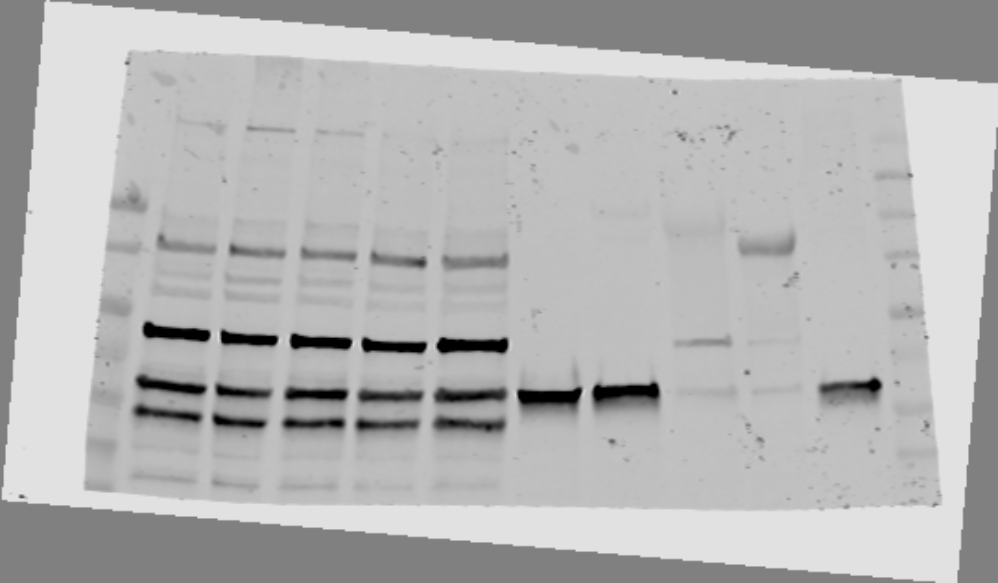

Supplement: Supplementary file 6 — Source Data [file 41467_2026_73227_MOESM6_ESM.zip › Source data/Western blots - uncropped images and replicates/Figure 2b/EGFP-ANKRD11 truncations IPs_antiTBLR1.png]

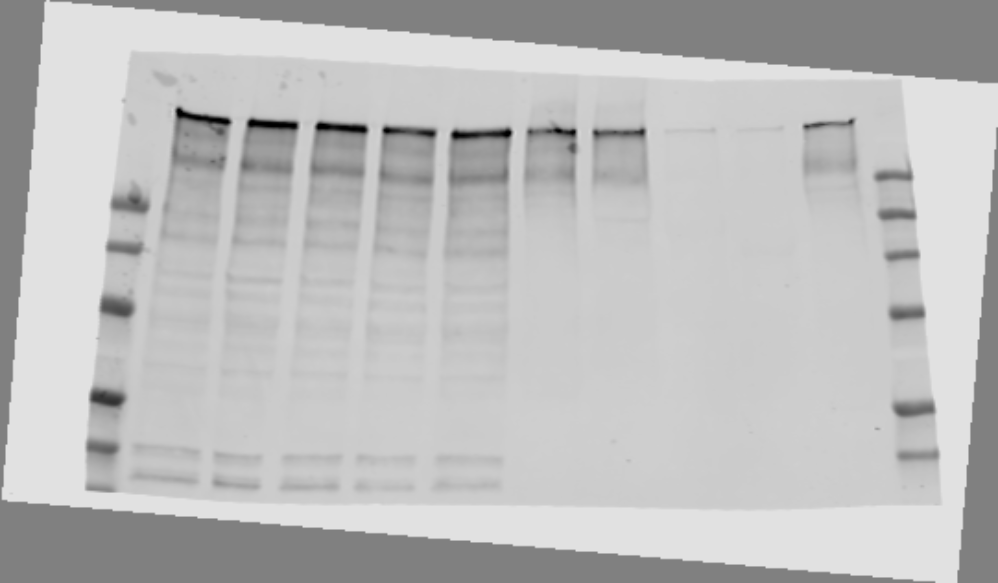

Supplement: Supplementary file 6 — Source Data [file 41467_2026_73227_MOESM6_ESM.zip › Source data/Western blots - uncropped images and replicates/Figure 2b/EGFP-ANKRD11 truncations IPs_antiNCOR1.png]

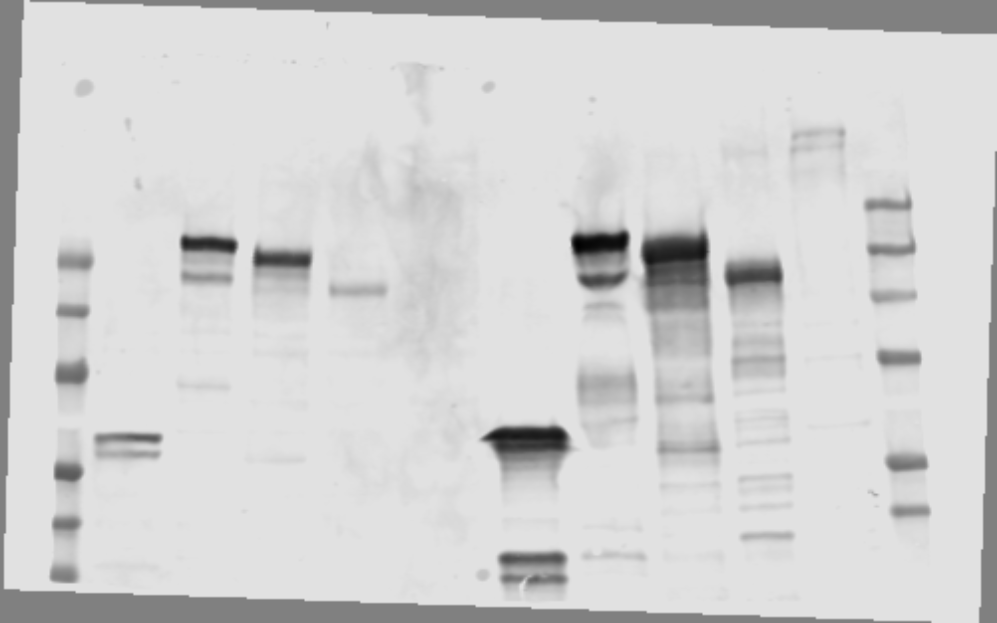

Supplement: Supplementary file 6 — Source Data [file 41467_2026_73227_MOESM6_ESM.zip › Source data/Western blots - uncropped images and replicates/Figure 2b/EGFP-ANKRD11 truncations IPs_antiGFP.png]

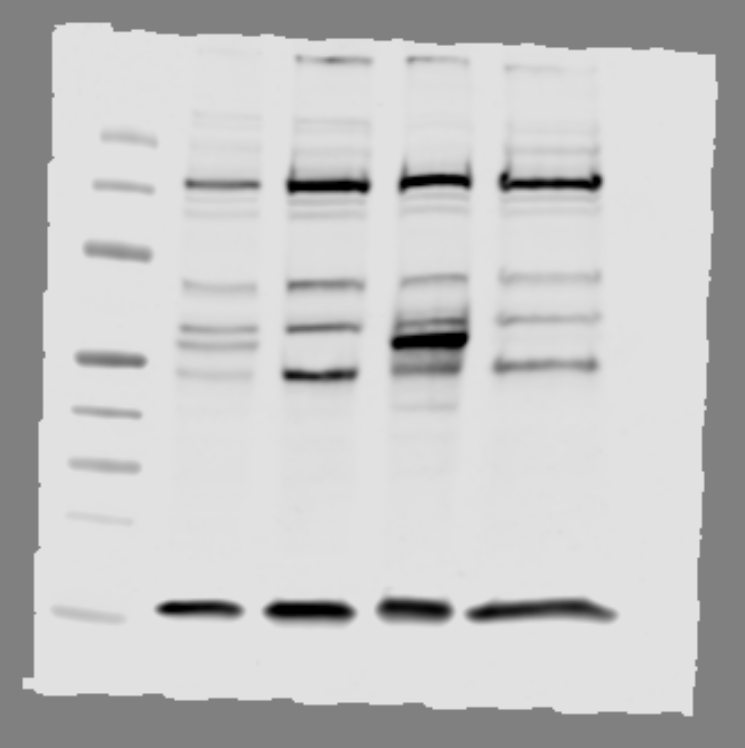

Supplement: Supplementary file 6 — Source Data [file 41467_2026_73227_MOESM6_ESM.zip › Source data/Western blots - uncropped images and replicates/Supplementary Figure 1a/TBLR1 knock-out TREx cell protein extracts_antiTBLR1_antiH3.png]

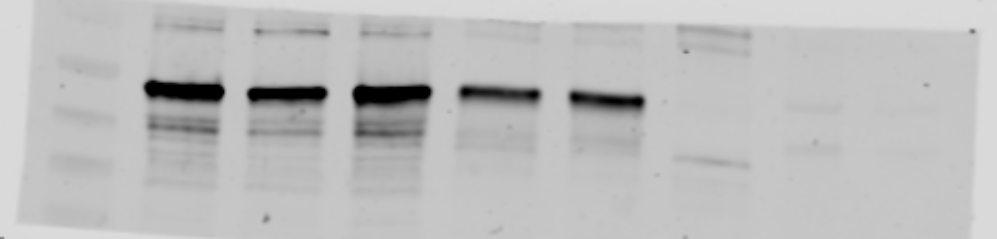

Supplement: Supplementary file 6 — Source Data [file 41467_2026_73227_MOESM6_ESM.zip › Source data/Western blots - uncropped images and replicates/Supplementary Figure 4d/EGFP-ANKRD11 C-term WT or S2475P in SETD5 WT or W834C ESCs_antiHDAC3 rerun gel 2.png]

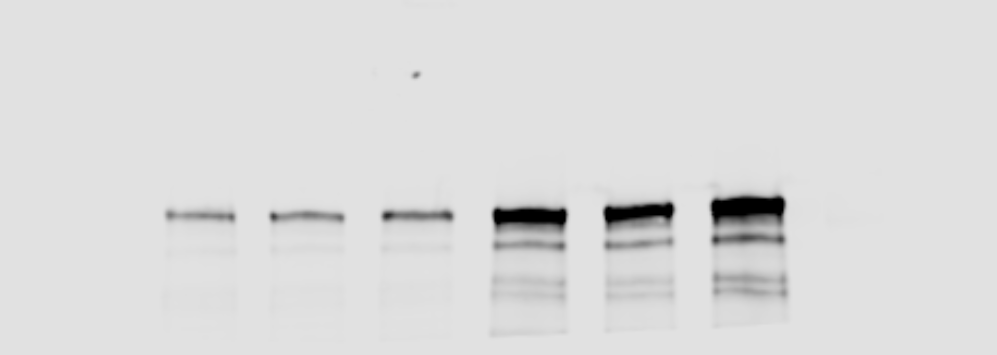

Supplement: Supplementary file 6 — Source Data [file 41467_2026_73227_MOESM6_ESM.zip › Source data/Western blots - uncropped images and replicates/Supplementary Figure 4d/EGFP-ANKRD11 C-term WT or S2475P in SETD5 WT or W834C ESCs_antiGFP rerun gel 2.png]

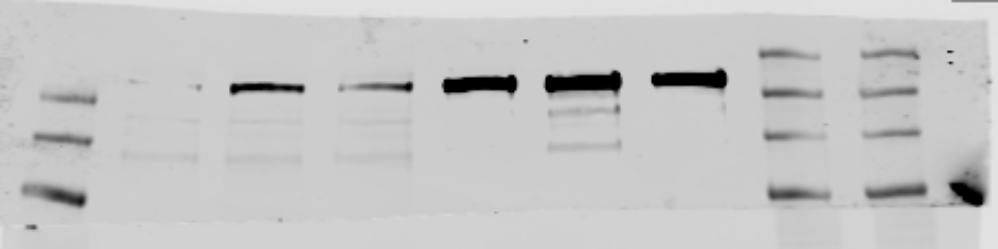

Supplement: Supplementary file 6 — Source Data [file 41467_2026_73227_MOESM6_ESM.zip › Source data/Western blots - uncropped images and replicates/Supplementary Figure 4d/EGFP-ANKRD11 C-term WT or S2475P in SETD5 WT or W834C ESCs_antiGFP.png]

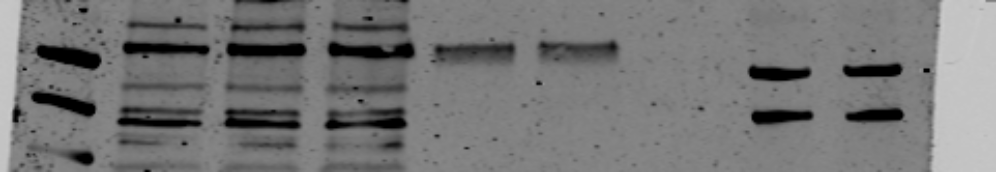

Supplement: Supplementary file 6 — Source Data [file 41467_2026_73227_MOESM6_ESM.zip › Source data/Western blots - uncropped images and replicates/Supplementary Figure 4d/EGFP-ANKRD11 C-term WT or S2475P in SETD5 WT or W834C ESCs_antiTBLR1.png]

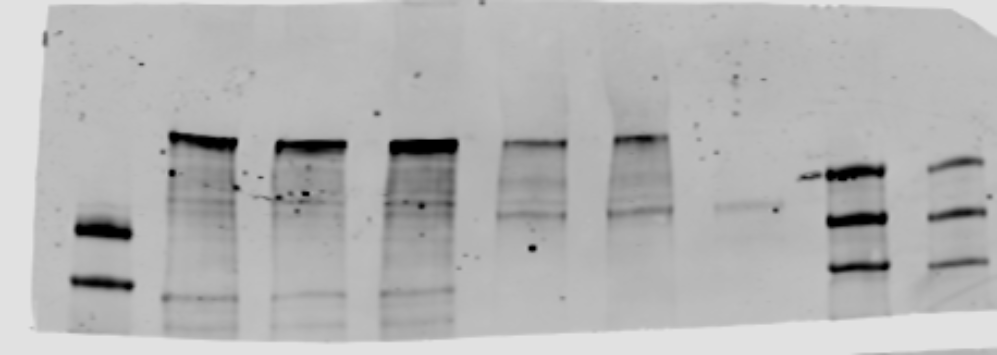

Supplement: Supplementary file 6 — Source Data [file 41467_2026_73227_MOESM6_ESM.zip › Source data/Western blots - uncropped images and replicates/Supplementary Figure 4d/EGFP-ANKRD11 C-term WT or S2475P in SETD5 WT or W834C ESCs_antiNCOR1 rerun gel 2.png]

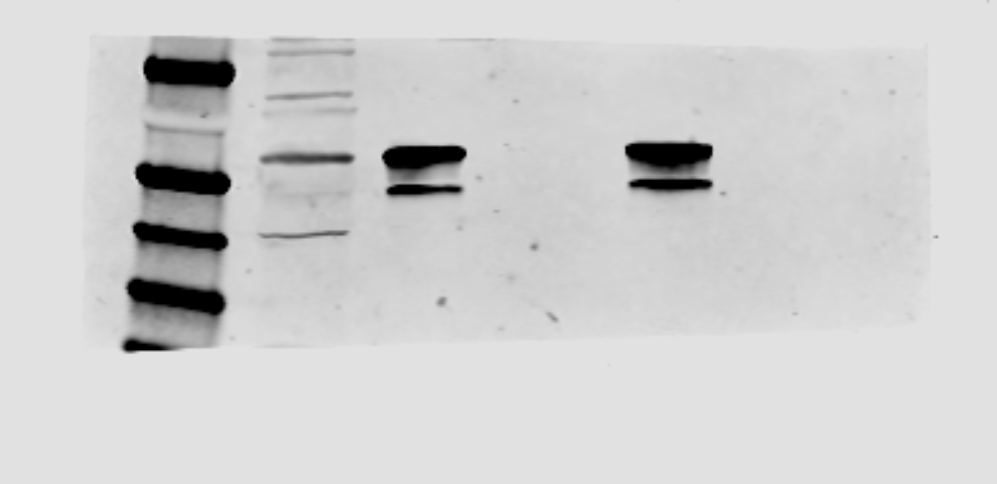

Supplement: Supplementary file 6 — Source Data [file 41467_2026_73227_MOESM6_ESM.zip › Source data/Western blots - uncropped images and replicates/Supplementary Figure 3b/MeCP2 SETD5 NID peptide pull down_mouse brain extracts_antiTBLR1_antiHDAC3.png]

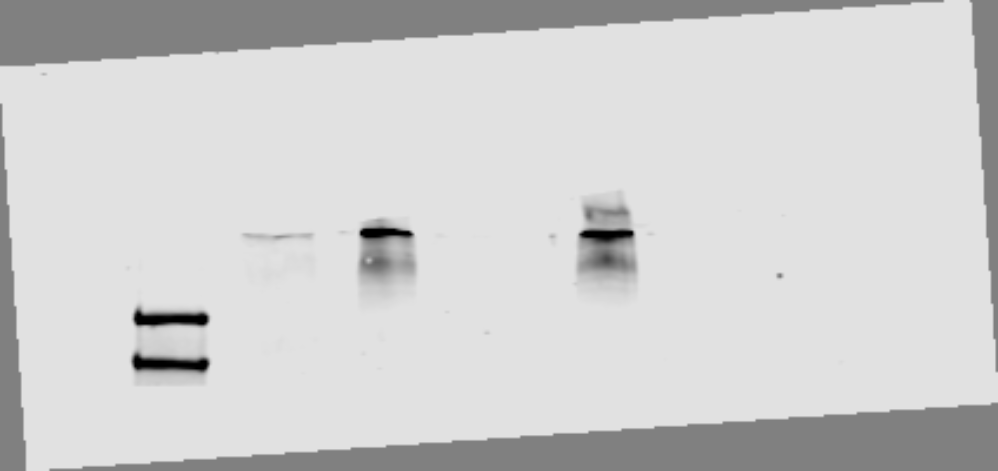

Supplement: Supplementary file 6 — Source Data [file 41467_2026_73227_MOESM6_ESM.zip › Source data/Western blots - uncropped images and replicates/Supplementary Figure 3b/MeCP2 SETD5 NID peptide pull down_mouse brain extracts_antiNCOR1.png]

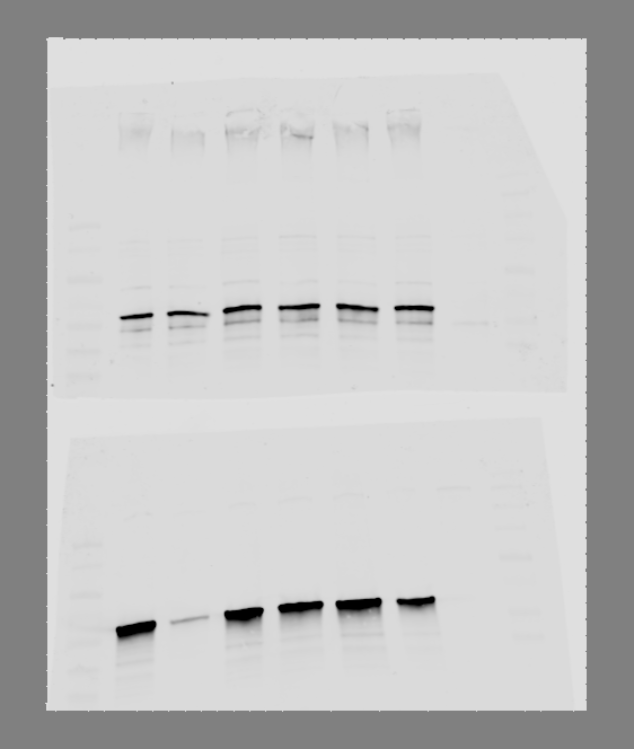

Supplement: Supplementary file 6 — Source Data [file 41467_2026_73227_MOESM6_ESM.zip › Source data/Western blots - uncropped images and replicates/Figure 3h/mCherry-SETD5 gnomAD mutants IPs_inputs top_IPs bottom_antiTBLR1.png]

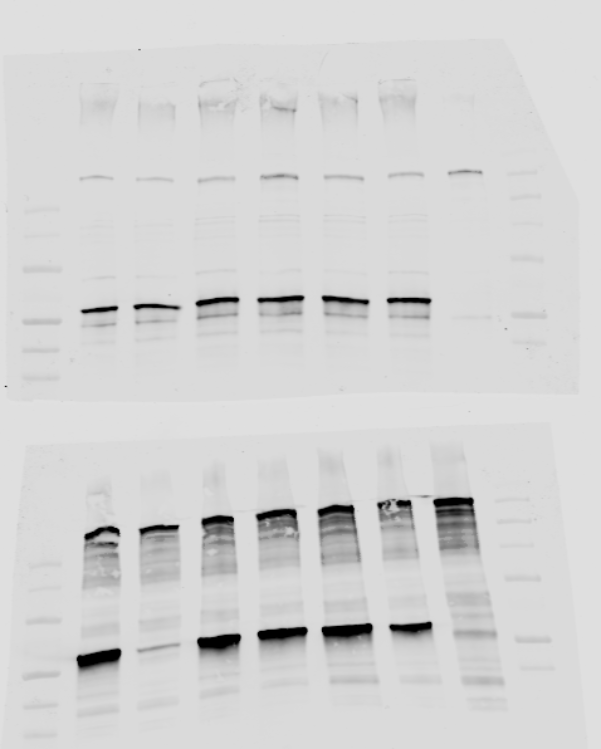

Supplement: Supplementary file 6 — Source Data [file 41467_2026_73227_MOESM6_ESM.zip › Source data/Western blots - uncropped images and replicates/Figure 3h/mCherry-SETD5 gnomAD mutants IPs_inputs top_IPs bottom_antiTBLR1_antimCherry.png]

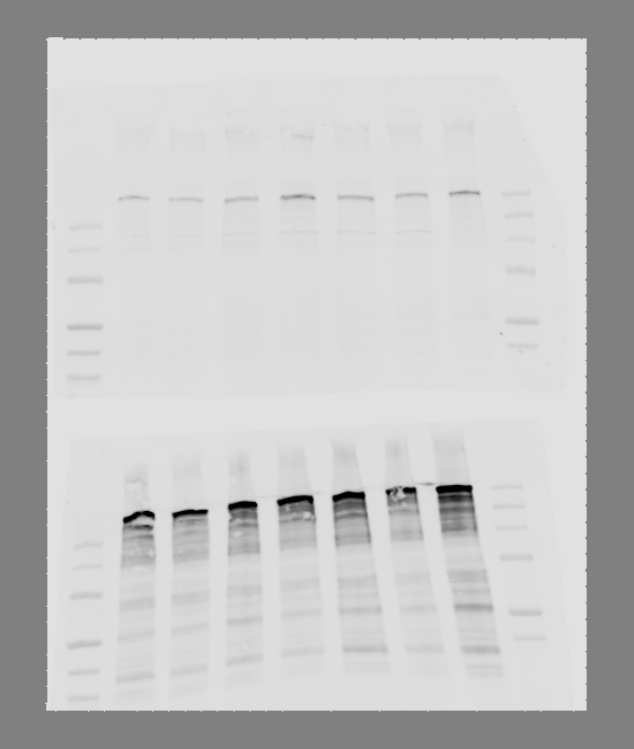

Supplement: Supplementary file 6 — Source Data [file 41467_2026_73227_MOESM6_ESM.zip › Source data/Western blots - uncropped images and replicates/Figure 3h/mCherry-SETD5 gnomAD mutants IPs_inputs top_IPs bottom_antimCherry.png]

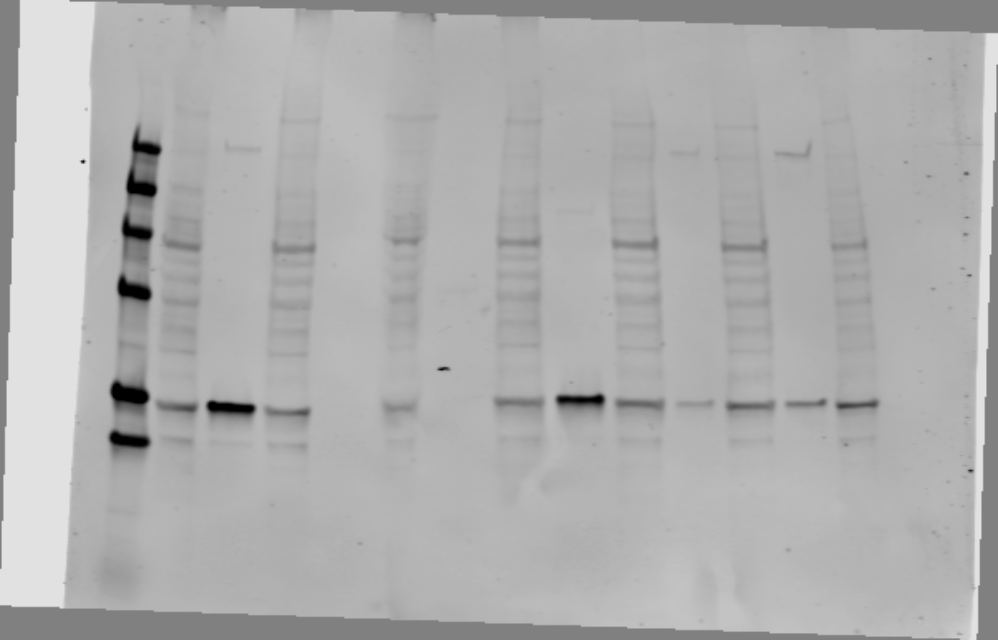

Supplement: Supplementary file 6 — Source Data [file 41467_2026_73227_MOESM6_ESM.zip › Source data/Western blots - uncropped images and replicates/Figure 3a/mCherry-SETD5 truncations IPs_antiHDAC3.png]

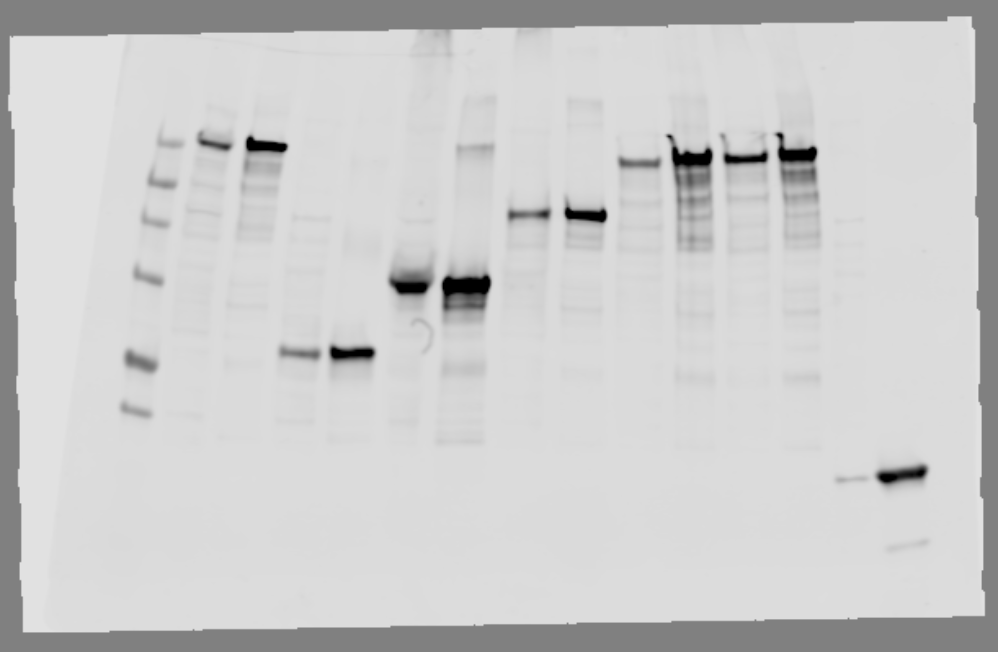

Supplement: Supplementary file 6 — Source Data [file 41467_2026_73227_MOESM6_ESM.zip › Source data/Western blots - uncropped images and replicates/Figure 3a/mCherry-SETD5 truncations IPs _antimCherry.png]

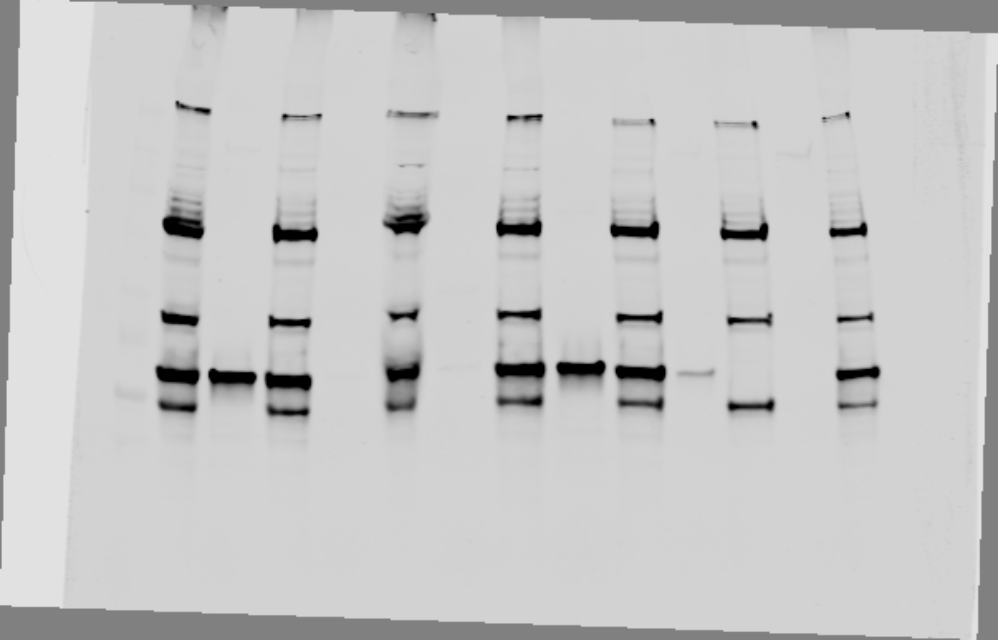

Supplement: Supplementary file 6 — Source Data [file 41467_2026_73227_MOESM6_ESM.zip › Source data/Western blots - uncropped images and replicates/Figure 3a/mCherry-SETD5 truncations IPs_antiTBLR1.png]

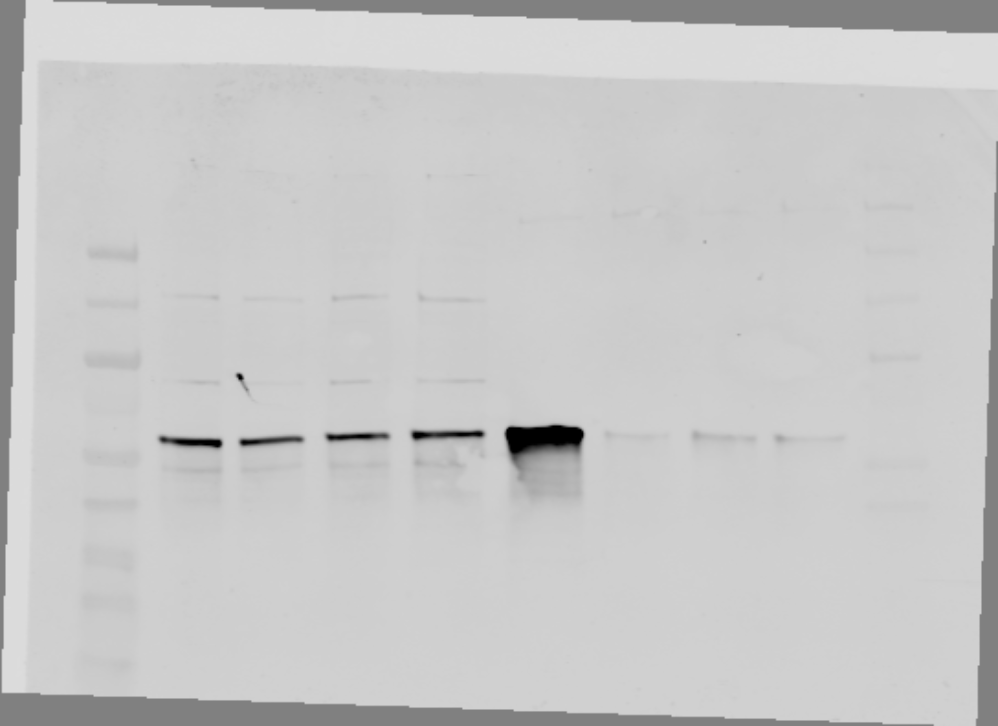

Supplement: Supplementary file 6 — Source Data [file 41467_2026_73227_MOESM6_ESM.zip › Source data/Western blots - uncropped images and replicates/Figure 3g/EGFP-SETD5 pathogenic mutants IPs_antiTBLR1.png]

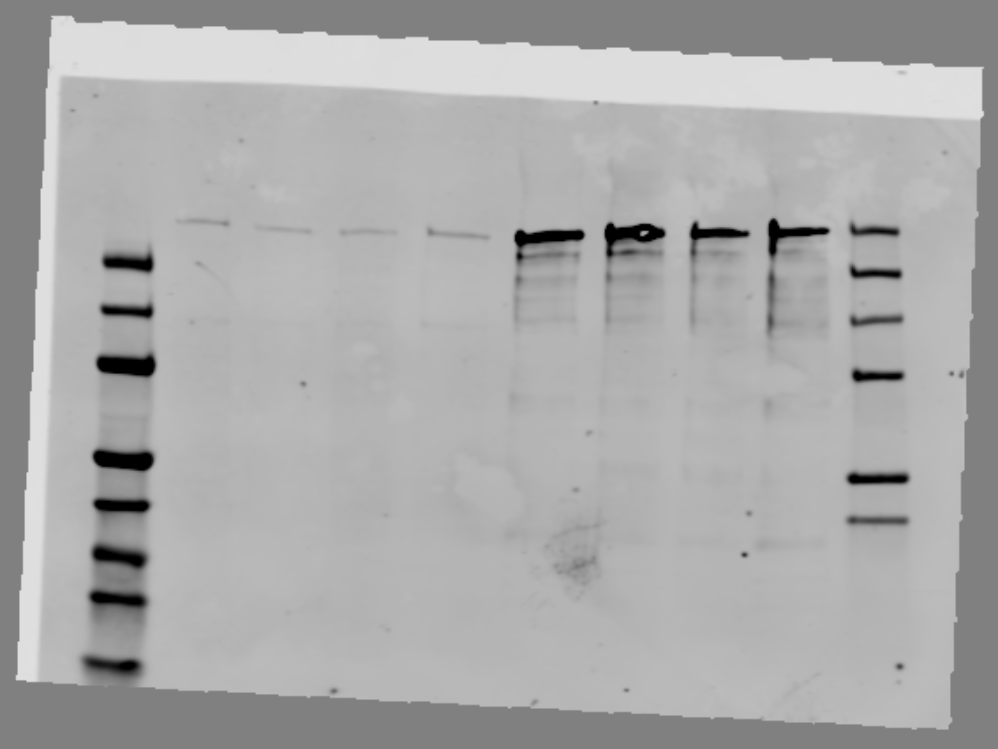

Supplement: Supplementary file 6 — Source Data [file 41467_2026_73227_MOESM6_ESM.zip › Source data/Western blots - uncropped images and replicates/Figure 3g/EGFP-SETD5 pathogenic mutants IPs_antiGFP.png]

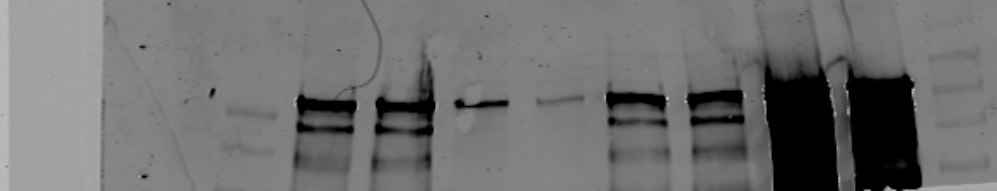

Supplement: Supplementary file 6 — Source Data [file 41467_2026_73227_MOESM6_ESM.zip › Source data/Western blots - uncropped images and replicates/Replicate western blots/Figure 1g/EGFP-ANKRD11 or mCherry-SETD5 IPs in TBLR1 WT and KO cells_antiGFP_high intensity.png]

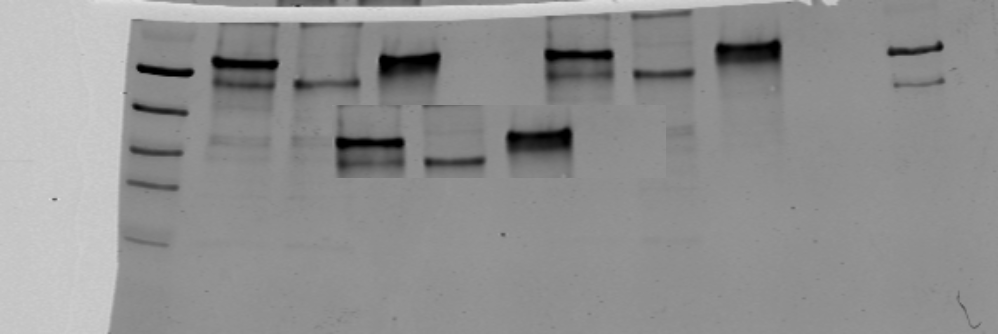

Supplement: Supplementary file 6 — Source Data [file 41467_2026_73227_MOESM6_ESM.zip › Source data/Western blots - uncropped images and replicates/Replicate western blots/Figure 1g/EGFP-ANKRD11 or mCherry-SETD5 IPs in TBLR1 WT and KO cells_antiTBLR1.png]

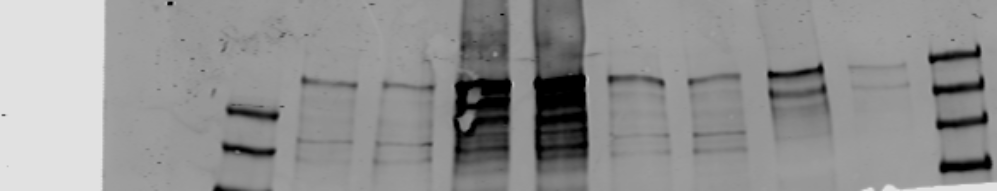

Supplement: Supplementary file 6 — Source Data [file 41467_2026_73227_MOESM6_ESM.zip › Source data/Western blots - uncropped images and replicates/Replicate western blots/Figure 1g/EGFP-ANKRD11 or mCherry-SETD5 IPs in TBLR1 WT and KO cells_antimCherry.png]

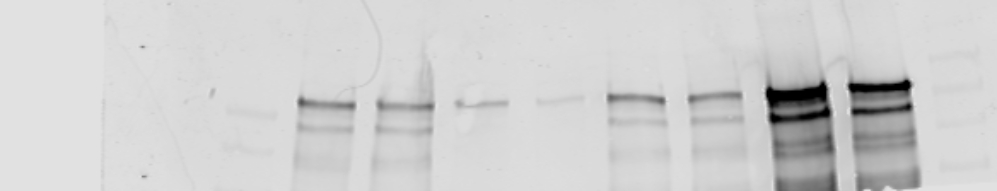

Supplement: Supplementary file 6 — Source Data [file 41467_2026_73227_MOESM6_ESM.zip › Source data/Western blots - uncropped images and replicates/Replicate western blots/Figure 1g/EGFP-ANKRD11 or mCherry-SETD5 IPs in TBLR1 WT and KO cells_antiGFP_low intensity.png]

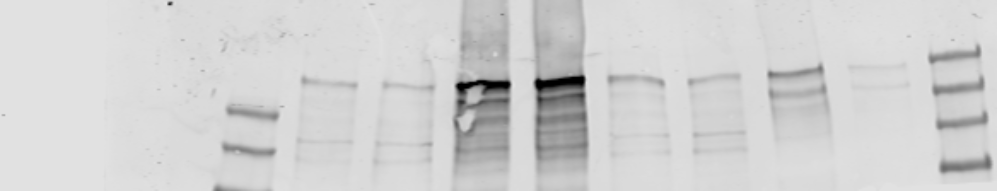

Supplement: Supplementary file 6 — Source Data [file 41467_2026_73227_MOESM6_ESM.zip › Source data/Western blots - uncropped images and replicates/Replicate western blots/Figure 1g/EGFP-ANKRD11 or mCherry-SETD5 IPs in TBLR1 WT and KO cells_antimCherry_low intensity.png]

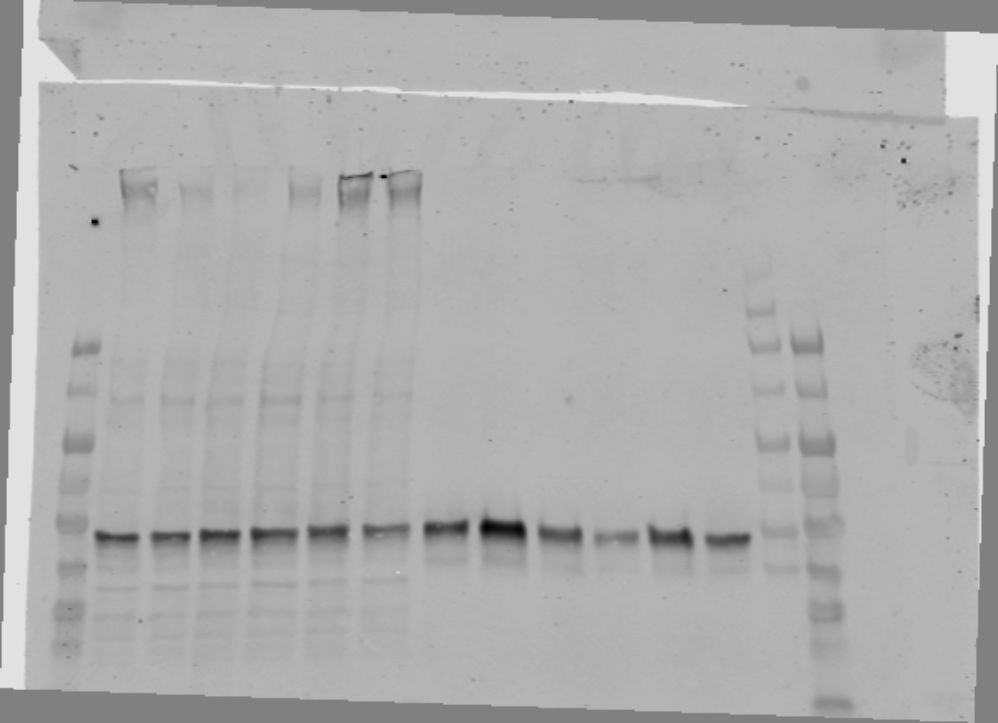

Supplement: Supplementary file 6 — Source Data [file 41467_2026_73227_MOESM6_ESM.zip › Source data/Western blots - uncropped images and replicates/Replicate western blots/Supplementary Figure 1c/mCherry-TBLR1 N-term mutants IPs in FLAG-TBLR1 transfected cells_antiHDAC3.png]

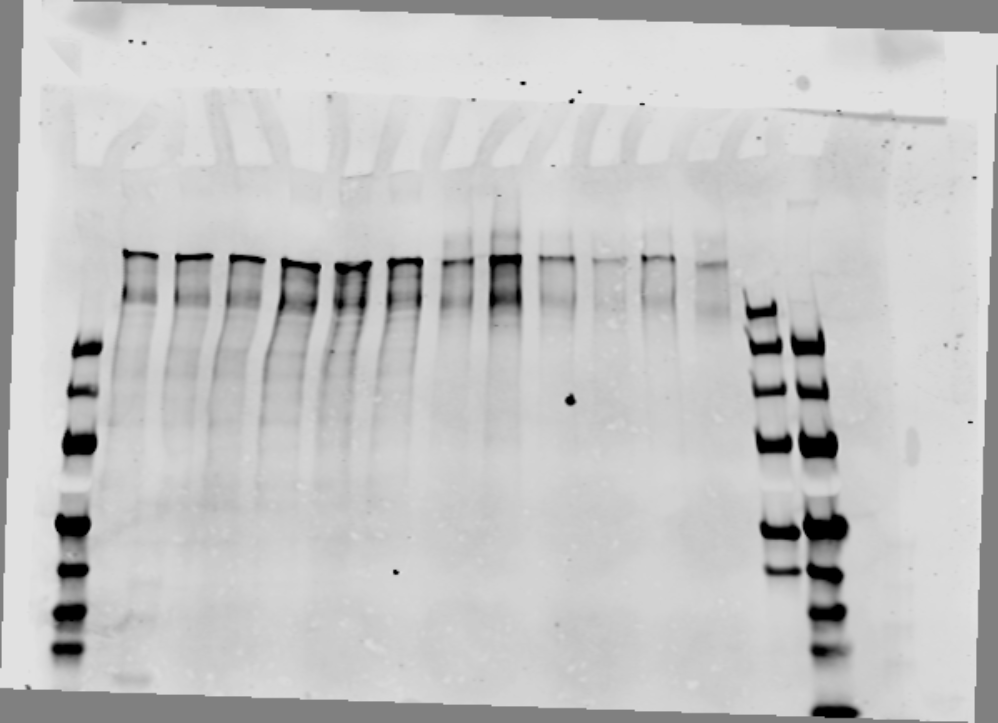

Supplement: Supplementary file 6 — Source Data [file 41467_2026_73227_MOESM6_ESM.zip › Source data/Western blots - uncropped images and replicates/Replicate western blots/Supplementary Figure 1c/mCherry-TBLR1 N-term mutants IPs in FLAG-TBLR1 transfected cells_antiNCOR1.png]

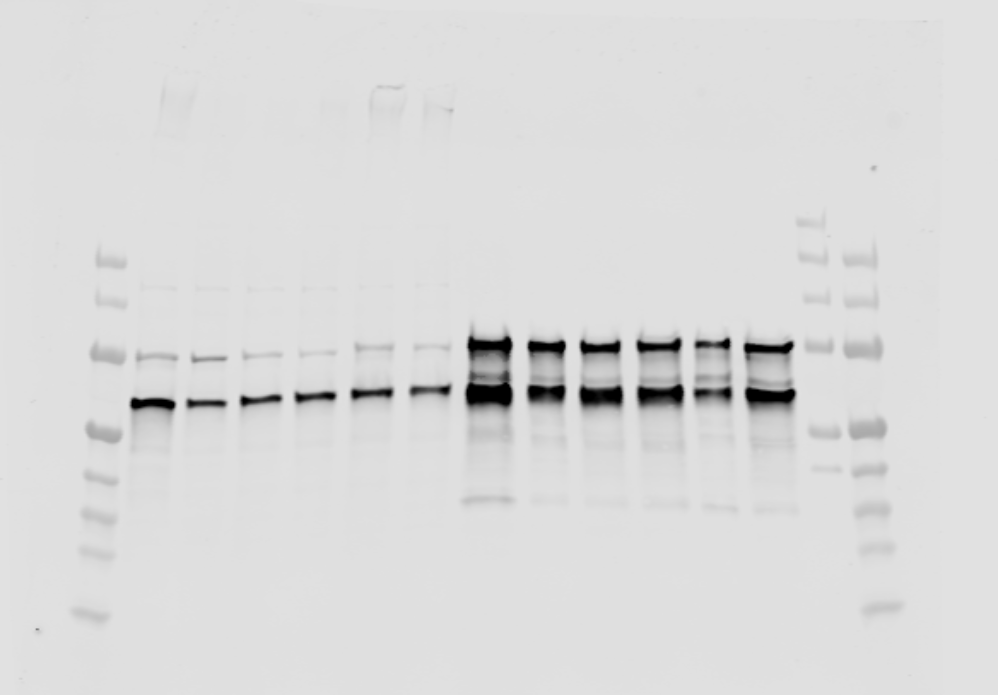

Supplement: Supplementary file 6 — Source Data [file 41467_2026_73227_MOESM6_ESM.zip › Source data/Western blots - uncropped images and replicates/Replicate western blots/Supplementary Figure 1c/mCherry-TBLR1 N-term mutants IPs in FLAG-TBLR1 transfected cells_antiFLAG.png]

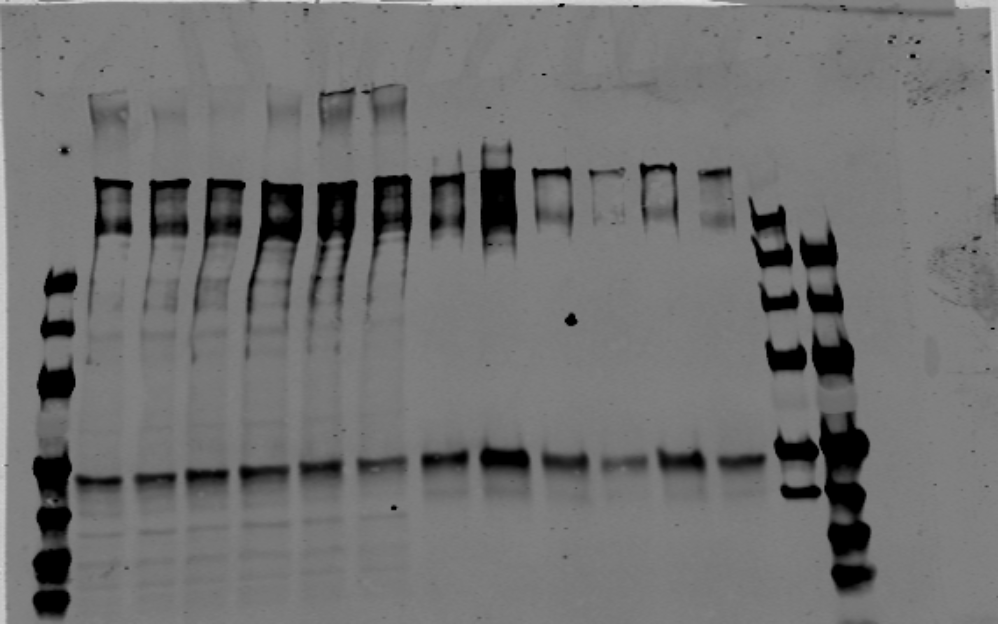

Supplement: Supplementary file 6 — Source Data [file 41467_2026_73227_MOESM6_ESM.zip › Source data/Western blots - uncropped images and replicates/Replicate western blots/Supplementary Figure 1c/mCherry-TBLR1 N-term mutants IPs in FLAG-TBLR1 transfected cells_antiNCOR1_antiHDAC3.png]

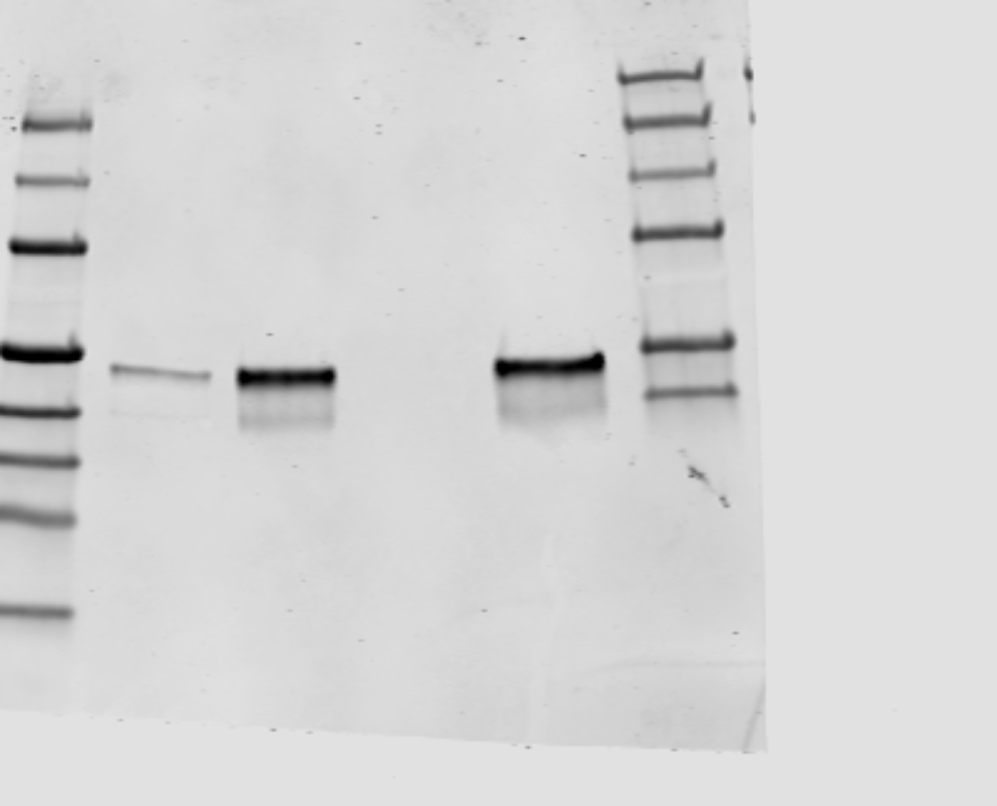

Supplement: Supplementary file 6 — Source Data [file 41467_2026_73227_MOESM6_ESM.zip › Source data/Western blots - uncropped images and replicates/Replicate western blots/Figure 3e/MeCP2 SETD5 NID peptide pulldown EGFP-ANKRD11(Cterm) transfected cells_rerun probed with_antiHDAC3.png]

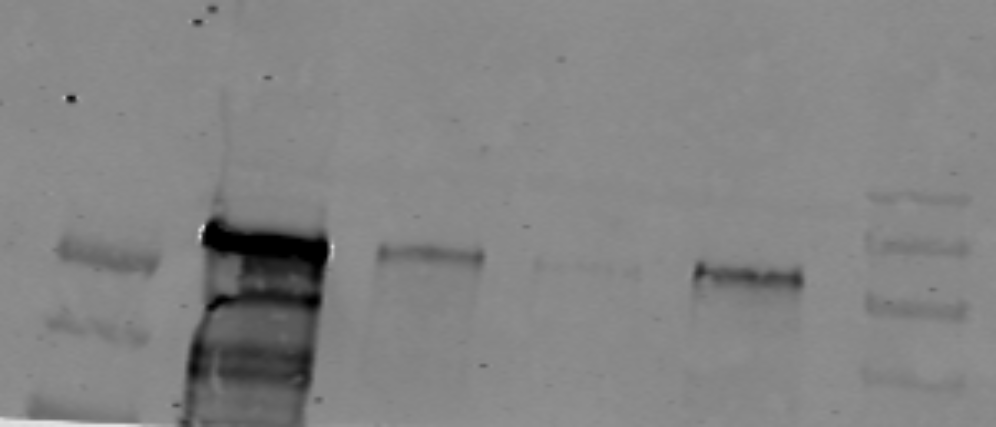

Supplement: Supplementary file 6 — Source Data [file 41467_2026_73227_MOESM6_ESM.zip › Source data/Western blots - uncropped images and replicates/Replicate western blots/Figure 3e/MeCP2 SETD5 NID peptide pulldown EGFP-ANKRD11(Cterm) transfected cells_antiGFP.png]

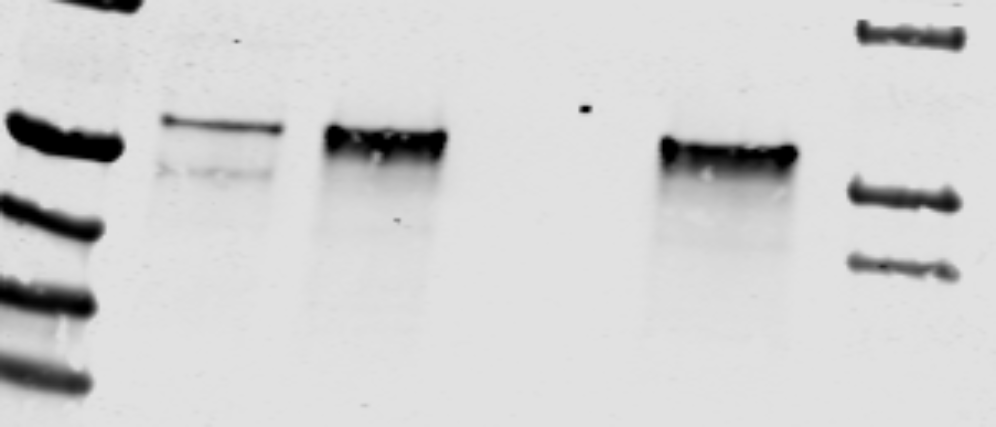

Supplement: Supplementary file 6 — Source Data [file 41467_2026_73227_MOESM6_ESM.zip › Source data/Western blots - uncropped images and replicates/Replicate western blots/Figure 3e/MeCP2 SETD5 NID peptide pulldown EGFP-ANKRD11(Cterm) transfected cells_antiTBLR1.png]

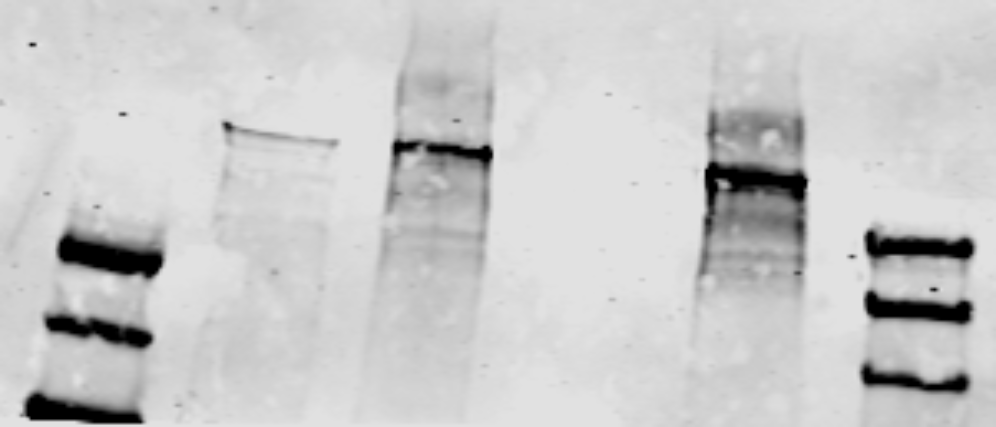

Supplement: Supplementary file 6 — Source Data [file 41467_2026_73227_MOESM6_ESM.zip › Source data/Western blots - uncropped images and replicates/Replicate western blots/Figure 3e/MeCP2 SETD5 NID peptide pulldown EGFP-ANKRD11(Cterm) transfected cells_antiNCOR1.png]

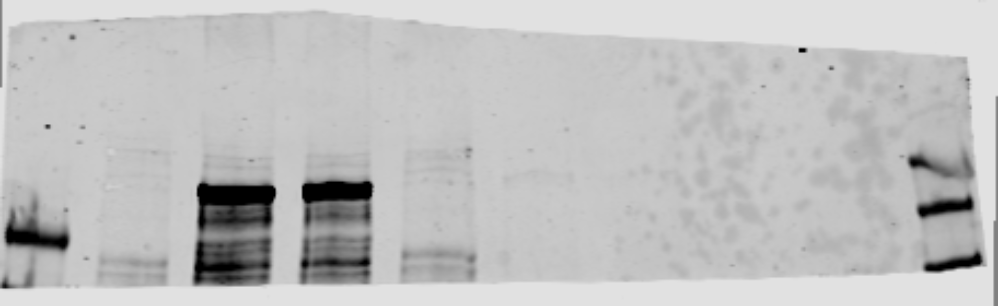

Supplement: Supplementary file 6 — Source Data [file 41467_2026_73227_MOESM6_ESM.zip › Source data/Western blots - uncropped images and replicates/Replicate western blots/Figure 5a/competition assay EGFP-MeCP2 IP from EGFP-MeCP2 + mCherry-SETD5 transfected extracts_antimCherry.png]

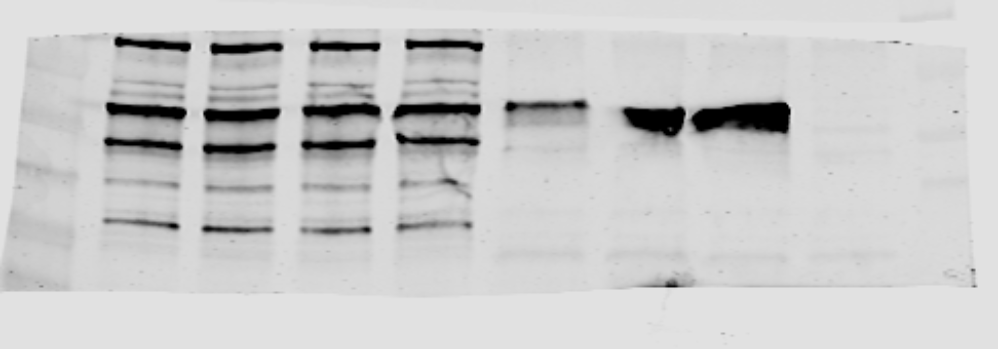

Supplement: Supplementary file 6 — Source Data [file 41467_2026_73227_MOESM6_ESM.zip › Source data/Western blots - uncropped images and replicates/Replicate western blots/Figure 5a/competition assay EGFP-MeCP2 IP from EGFP-MeCP2 + mCherry-SETD5 transfected extracts_antiTBLR1.png]

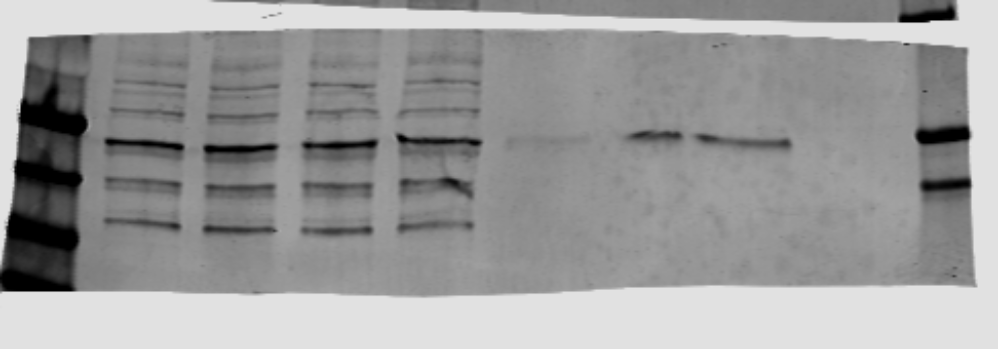

Supplement: Supplementary file 6 — Source Data [file 41467_2026_73227_MOESM6_ESM.zip › Source data/Western blots - uncropped images and replicates/Replicate western blots/Figure 5a/competition assay EGFP-MeCP2 IP from EGFP-MeCP2 + mCherry-SETD5 transfected extracts_antiHDAC3.png]

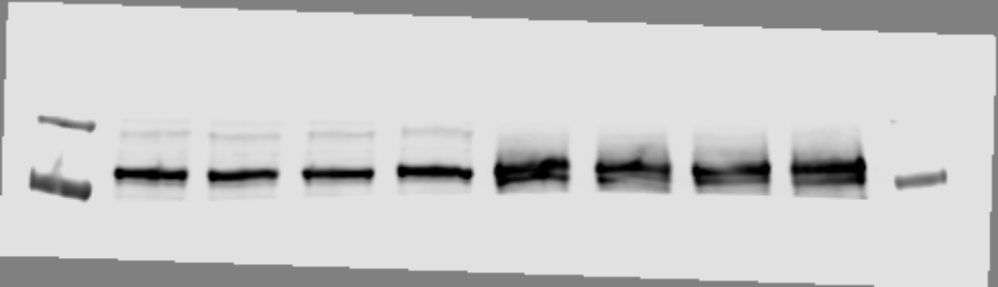

Supplement: Supplementary file 6 — Source Data [file 41467_2026_73227_MOESM6_ESM.zip › Source data/Western blots - uncropped images and replicates/Replicate western blots/Figure 5a/competition assay EGFP-MeCP2 IP from EGFP-MeCP2 + mCherry-SETD5 transfected extracts_antiMeCP2.png]

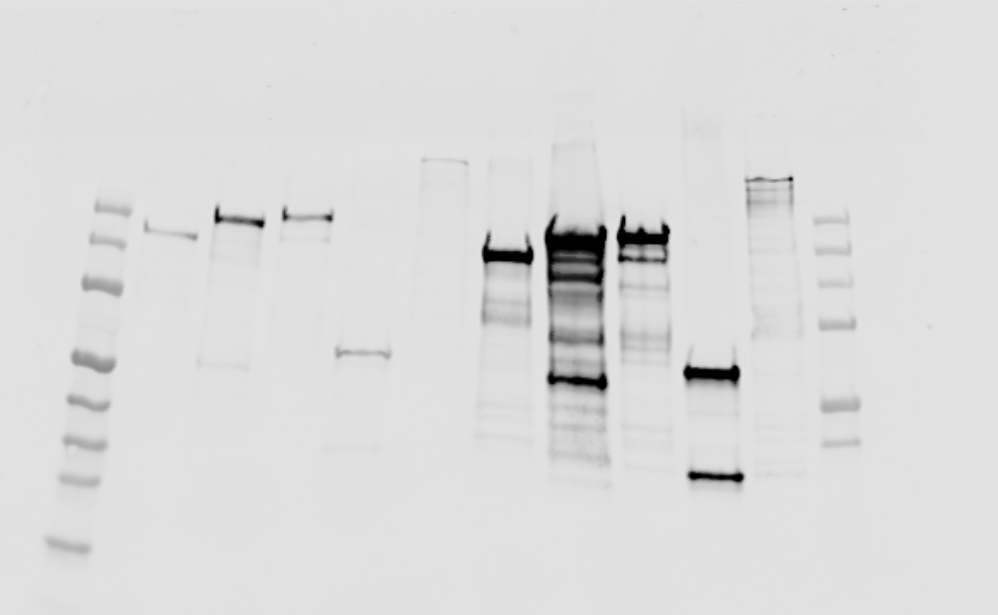

Supplement: Supplementary file 6 — Source Data [file 41467_2026_73227_MOESM6_ESM.zip › Source data/Western blots - uncropped images and replicates/Replicate western blots/Figure 2b/EGFP-ANKRD11 truncations IPs_antiEGFP.png]

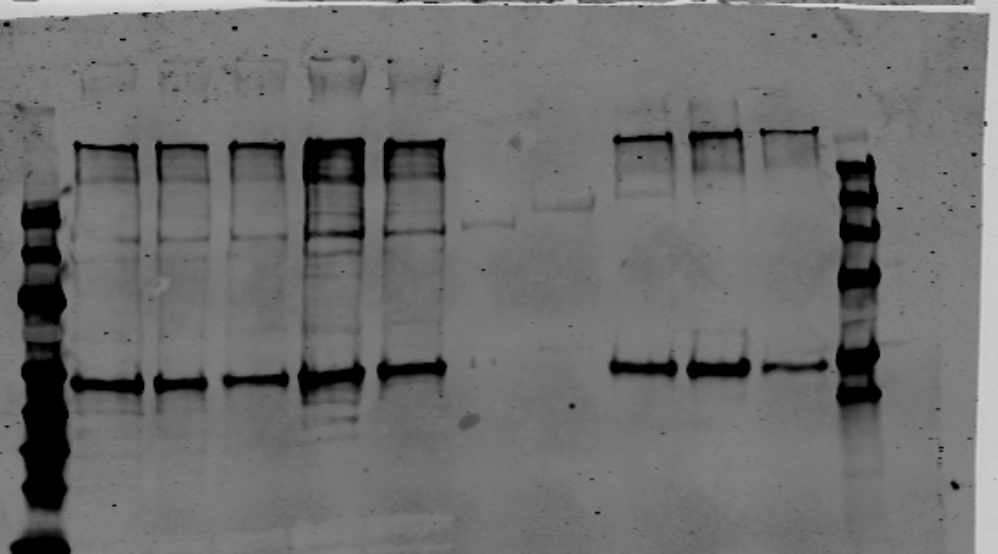

Supplement: Supplementary file 6 — Source Data [file 41467_2026_73227_MOESM6_ESM.zip › Source data/Western blots - uncropped images and replicates/Replicate western blots/Figure 2b/EGFP-ANKRD11 truncations IPs_antiNCOR1_antiHDAC3.png]

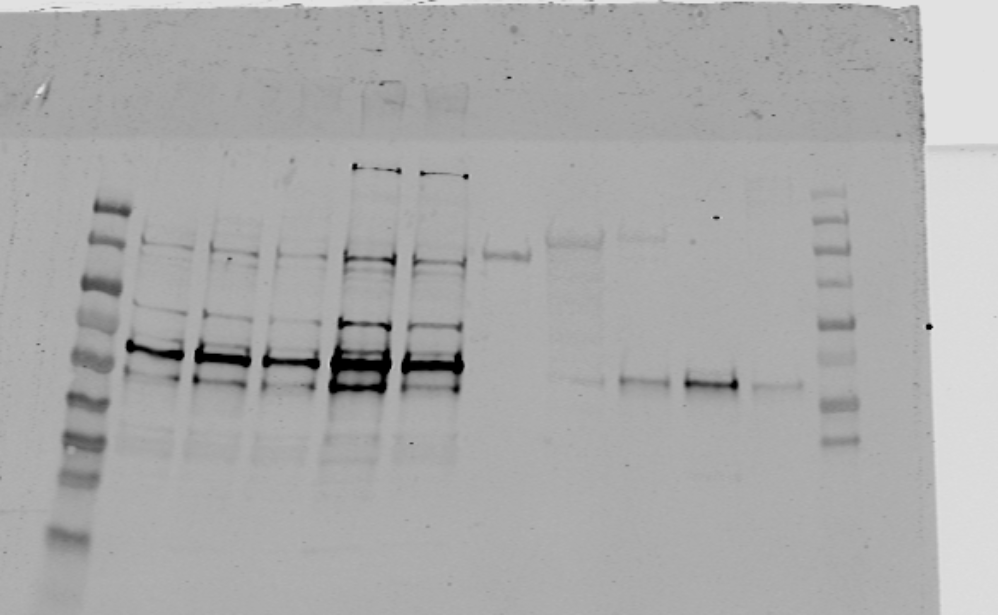

Supplement: Supplementary file 6 — Source Data [file 41467_2026_73227_MOESM6_ESM.zip › Source data/Western blots - uncropped images and replicates/Replicate western blots/Figure 2b/EGFP-ANKRD11 truncations IPs_antiEGFP_antiTBLR1.png]

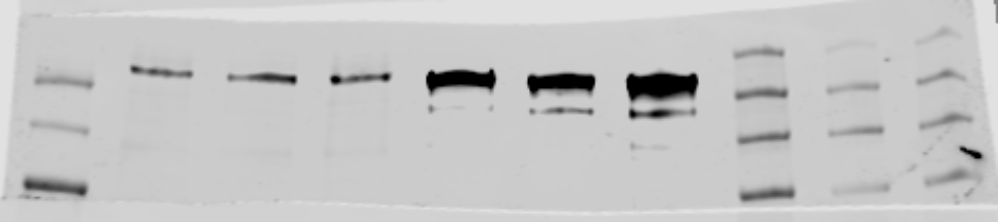

Supplement: Supplementary file 6 — Source Data [file 41467_2026_73227_MOESM6_ESM.zip › Source data/Western blots - uncropped images and replicates/Replicate western blots/Supplementary Figure 4d/EGFP-ANKRD11 C-term WT or S2475P in SETD5 WT or W834C ESCs_antiGFP.png]

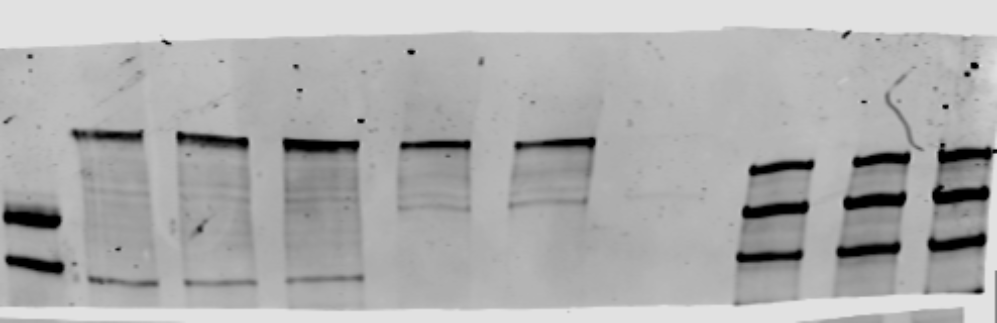

Supplement: Supplementary file 6 — Source Data [file 41467_2026_73227_MOESM6_ESM.zip › Source data/Western blots - uncropped images and replicates/Replicate western blots/Supplementary Figure 4d/EGFP-ANKRD11 C-term WT or S2475P in SETD5 WT or W834C ESCs_antiNCOR1_rerun gel 2.png]

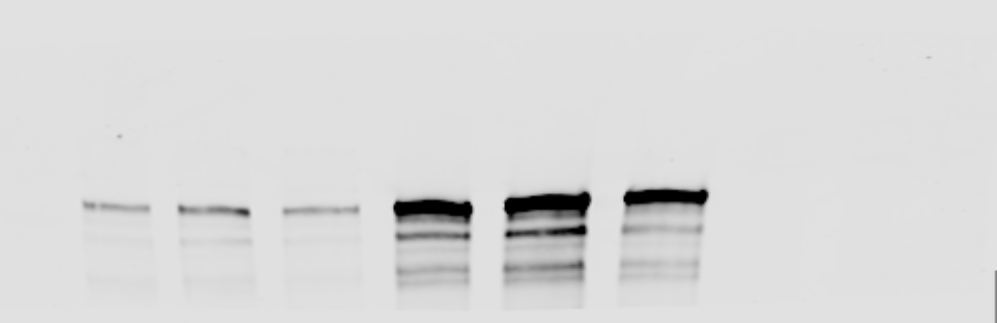

Supplement: Supplementary file 6 — Source Data [file 41467_2026_73227_MOESM6_ESM.zip › Source data/Western blots - uncropped images and replicates/Replicate western blots/Supplementary Figure 4d/EGFP-ANKRD11 C-term WT or S2475P in SETD5 WT or W834C ESCs_antiGFP_rerun gel 2.png]

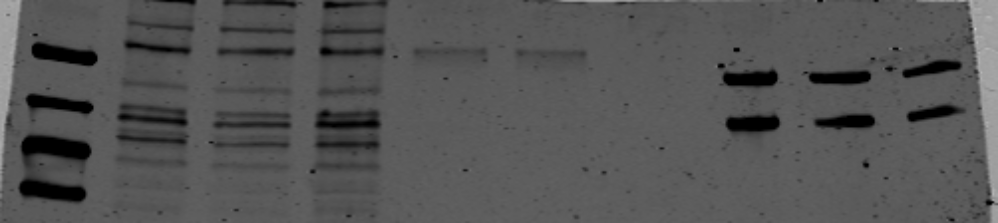

Supplement: Supplementary file 6 — Source Data [file 41467_2026_73227_MOESM6_ESM.zip › Source data/Western blots - uncropped images and replicates/Replicate western blots/Supplementary Figure 4d/EGFP-ANKRD11 C-term WT or S2475P in SETD5 WT or W834C ESCss_antiTBLR1.png]

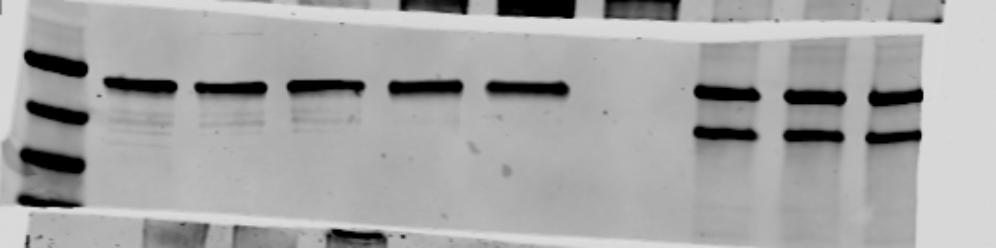

Supplement: Supplementary file 6 — Source Data [file 41467_2026_73227_MOESM6_ESM.zip › Source data/Western blots - uncropped images and replicates/Replicate western blots/Supplementary Figure 4d/EGFP-ANKRD11 C-term WT or S2475P in SETD5 WT or W834C ESCs_amHDAC3_rerun gel 2.png]

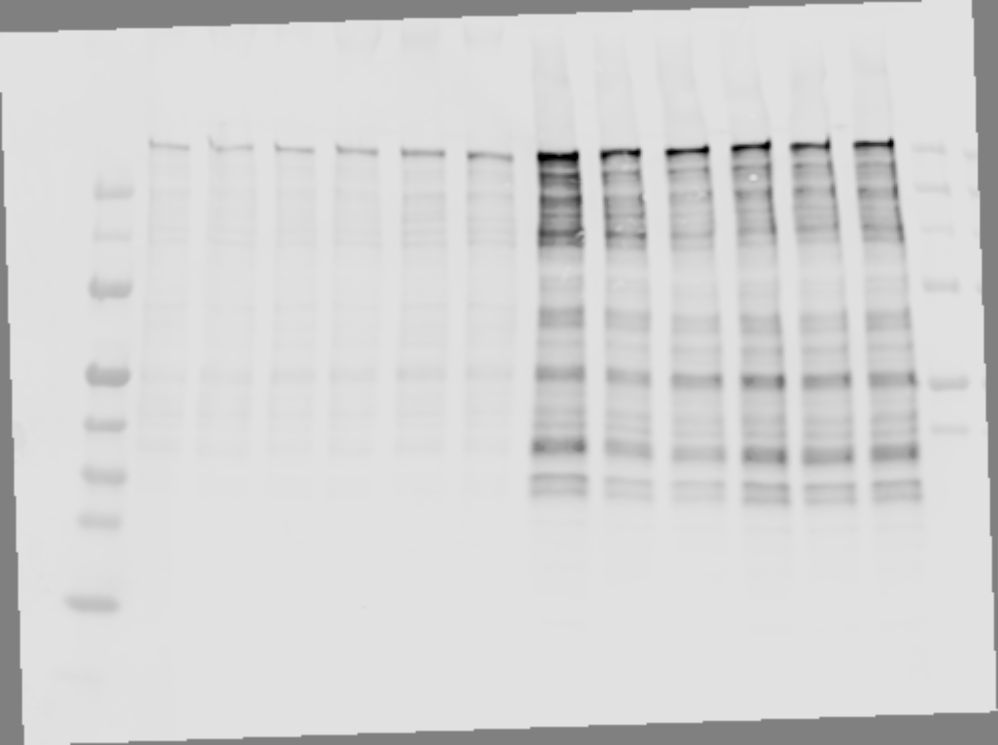

Supplement: Supplementary file 6 — Source Data [file 41467_2026_73227_MOESM6_ESM.zip › Source data/Western blots - uncropped images and replicates/Replicate western blots/Figure 3h/mCherry-SETD5 gnomAD mutants_antimCherry.png]

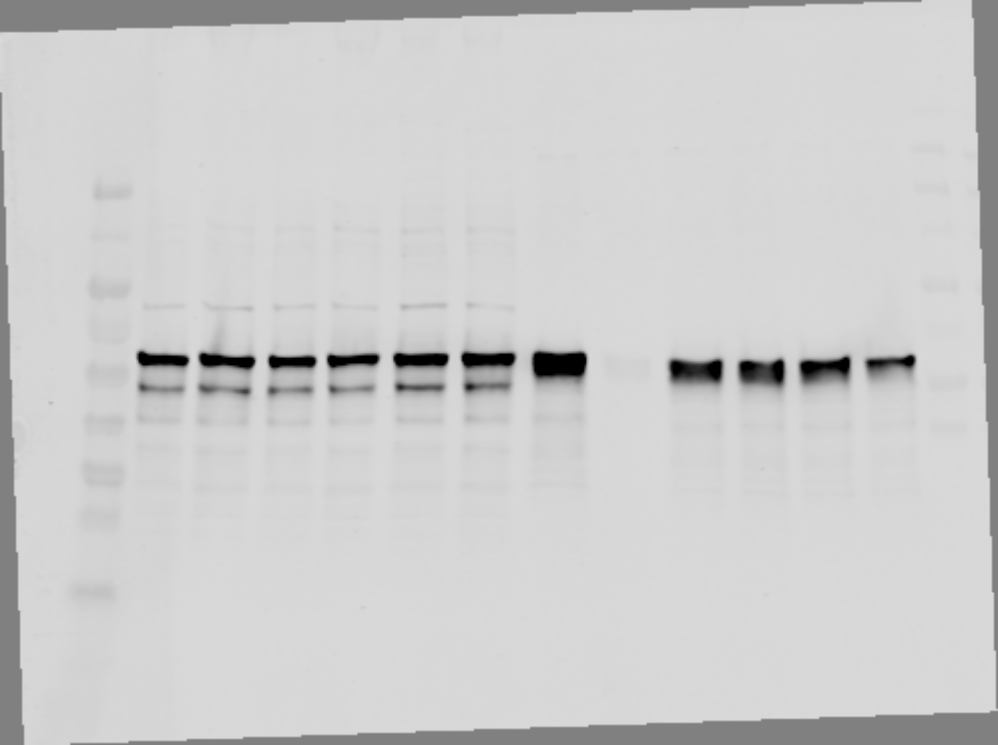

Supplement: Supplementary file 6 — Source Data [file 41467_2026_73227_MOESM6_ESM.zip › Source data/Western blots - uncropped images and replicates/Replicate western blots/Figure 3h/mCherry-SETD5 gnomAD mutants IPs_antiTBLR1.png]

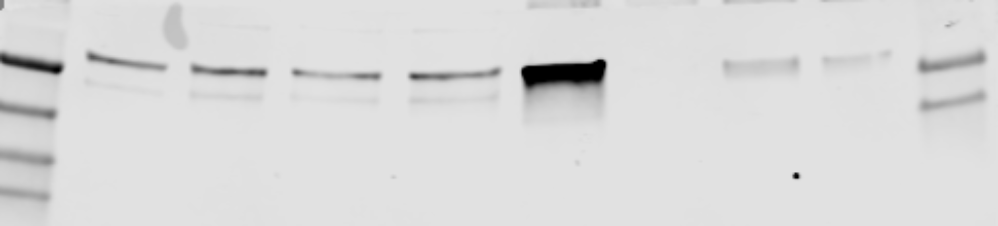

Supplement: Supplementary file 6 — Source Data [file 41467_2026_73227_MOESM6_ESM.zip › Source data/Western blots - uncropped images and replicates/Replicate western blots/Figure 3g/EGFP-SETD5 pathogenic mutants IPs_antiTBLR1.png]

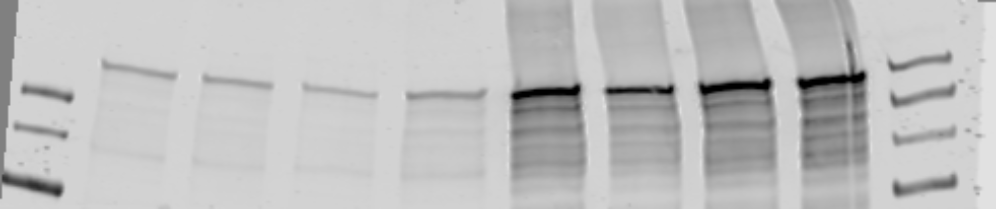

Supplement: Supplementary file 6 — Source Data [file 41467_2026_73227_MOESM6_ESM.zip › Source data/Western blots - uncropped images and replicates/Replicate western blots/Figure 3g/EGFP-SETD5 pathogenic mutants IPs_antiGFP.png]
