# Supplementary material for: Dysfunction of a SET3-like complex underlies a family of related neurological disorders
Source: Nat Commun. 2026 May 16;17:6729. doi: 10.1038/s41467-026-73227-5 (PMC13385800; doi:10.1038/s41467-026-73227-5)
Supplement: Supplementary file 5 — Transparent Peer Review file [file 41467_2026_73227_MOESM5_ESM.pdf]

nature portfolio

Peer Review File

Dysfunction of a SET3-like complex underlies a family of related neurological disorders

Corresponding Author: Professor Adrian Bird

Parts of this Peer Review File have been redacted as indicated to maintain the confidentiality of unpublished data.

This file contains all reviewer reports in order by version, followed by all author rebuttals in order by version.

Version 0:

Reviewer comments:

Reviewer #1

(Remarks to the Author)
The accumulating identification of de novo mutations in various chromatin regulators as the basis for monogenic neurodevelopmental disorders, several which present overlapping phenotypes, potentially indicates functional commonalities and a deeper level of understanding that will illuminate future medical practice. The manuscript by Paton et al describes connections underpinning several monogenic neurodevelopmental disorders notably connecting known neurodevelopmental mutations in Ankrd11, Setd5 and Tblr5 into a mechanistic context. This is important work from one of the forefront laboratories that complements and extends ongoing progress in the field. The manuscript is lucid, thoroughly referenced and a pleasure to read.

A few minor comments –
1. Details of the transient expression methods used to evaluate protein-protein interactions are missing. There are several different ways to achieve transient expression and they have very different properties, mostly involving modest to extreme overexpression. If the authors used extreme overexpression, then many readers, this reviewer included, will be have skepticism skeptical evaluate these protein-protein interaction data with high. Given the other qualities of this manuscript, the skepticism is unlikely but nevertheless, the authors should accurately describe this aspect of their experiments.
2. The identified relationship between the mammalian and yeast SET3Cs is motivating but perhaps the mammalian version should be denoted as mSET3C, or something similar.
3. Line 256 3.6% of Setd5W834C/W834C survived and were fertile – and crossed to Setd5W834C/+, resulting in a higher (unspecified) survival rate, from which the authors conclude selection for a secondary mutation. It is very unlikely that selection for a secondary mutation could be achieved in inbred strains in mouse house crosses. More likely this is due to either epigenetic or incompletely penetrant mechanisms.
4. Line 298 These data
5. Line 315 – Ref 38 appears mistakenly cited
Data from the 100k genome project places ANKRD11, SETD5 and MECP2 within the top ten most frequently mutated genes contributing to monogenic neurodevelopmental abnormalities 38.

Reviewer #2

(Remarks to the Author)
In this study Paton, Bird, Lyst and colleagues investigate developmental disorder associated gene mutations that disrupt chromatin protein interactions to define new connections between disorders. They characterize a putative functional homolog of the yeast SET3C chromatin regulatory complex that is composed of ANKRD11 and SETD5 interacting with the NCoR corepressor complex through the TBLR1 subunit. Building off previous findings suggesting that these proteins interact and genetic studies showing the mutations in each of these proteins cause related developmental disorders, the authors characterize the effects of missense mutations in TBLR1 on interactions with SETD5 and ANKRD11. They show that mutations in the N-terminal domain of TBLR1 disrupt ANKDR11 association while mutations in the C-terminal WDR domain disrupt SETD5 association. They then show that disease-associated mutations in the C-terminal domain of ANKRD11 disrupt interaction with TBLR1. Turning to SETD5, they find that it interacts with TBLR1 through a peptide

sequence similar to a core TBLR1-binding sequence of the Rett syndrome protein MeCP2, and show that disease-associated mutations in this sequence disrupt the SETD5-TBLR1 interaction. To further investigate the SETD5-TBLR1 interaction they generate and study a SETD5 W834C mutant mouse, showing that this mutation disrupts TBLR1 interaction in vivo and leads to disease relevant phenotypes. Finally, to examine the functional impact of disrupting the putative ANKRD11/SETD5/NCoR complex the authors perform RNA-seq on ES cells with either SETD5 or ANKRD11 mutations that disrupt NCoR binding and analyze existing perturb-seq data. They find highly overlapping effects for disruption of ANKRD11, SETD5, and NCoR components, supporting the notion that these proteins function in an overlapping pathway. Overall, this study does an impressive job of establishing interactions in the putative mammalian SET3C complex and dissecting the effects of disease mutations on these interactions. This work defines a new group of related disorders that impact shared chromatin biology. The experiments are rigorously executed, and the findings are convincing. The study identifies molecular interactions between distinct genetic causes of neurodevelopmental diseases, beginning to define generalized disease-associated pathways that the field is searching for. It provides timely and impactful findings that will be of interest to many investigators and the focus of future studies across numerous labs.
As it is currently presented however, the study misses some chances to fully investigate the interactions of the putative SET3C complex and does not provide insight into the mechanisms leading to transcriptional disruption occurring in mutants in vivo. Further, the study posits addressable questions about the interplay between MeCP2 and this complex, but does not include analyses to address these questions. In my opinion, some or all of these gaps should be addressed before the manuscript is ready for publication in Nature Communications.

Major issues:

1.The authors propose that SETD5 and ANKRD11 work together with NCoR as part of a mammalian “SET3C” complex, but additional experiments to test the roles of SETD5 and ANKRD11 in this complex are warranted. The authors could use their cell lines or mutant constructs to determine whether mutations affecting SETD5 and ANKRD11 association with NCoR disrupts either the protein levels of NCoR components or NCoR intra-complex interactions. This would determine whether SETD5 and/or ANKRD11 function to stabilize or scaffold the SET3C complex, respectively. An additional possibility would be that SETD5 and/or ANKRD11 impact the recruitment of SET3C to chromatin, which could be tested using ChIP-seq targeting NCoR components in SETD5 and ANKRD11 mutant cell lines, or in the SETD5 W834C mouse. Evaluating whether the NCoR complex remains intact after disease-associated mutations in SETD5 or ANKRD11 would provide clearer insight into the contribution of these proteins to the mammalian SET3C complex.

2.The direct mechanism and function of SET3C-like complex in mammalian models is not addressed, despite testable hypotheses stemming from what is known about the components of the complex. Does SET3C complex in mammals show a molecular function comparable to what is known in the yeast? The authors should be able investigate these mechanisms to understand how transcriptional disruption occurs when SET3C components are mutated. For example, disruption of SETD5 has been shown to have effects on H3K36 methylation-associated transcriptional regulation, and NCoR is well established to function as a histone deacetylase, but the authors don’t perform complementary histone modification profiling experiments to investigate these marks. In addition, they do not attempt ChIP-seq with antibodies to NCoR or members of the SET3C complex to investigate if changes in their recruitment to chromatin underlie a subset of transcriptomic effects. This work could be done in the cell lines they have performed RNA-seq on, or, even better, in the brain tissue of the SETD5W834C/+ mouse. In addition to providing mechanism, the results could address which effects on transcription may directly result from loss of complex function.

3.While the study uses robust assays in cell lines to assess interactions and transcriptomic effects, the follow up in vivo is limited, making the study’s conclusions about impact on biology less robust. The study would be strengthened if it included transcriptomic analysis from brain tissue from the newly generated SETD5W834C/+ mouse. These data could be used to probe the biological consequences of disrupt SET3C and also be utilized in mechanistic studies (see comment above) and interrogation of MECP2 interactions (see below).

4.The authors propose that competition for TBLR1 binding between MeCP2 and SETD5 may occur, but they don’t investigate this possibility using existing data or perform tractable experiments to test this possibility. For example, the authors could examine the mass spec data from Figure 4D to see if NCoR1 interacts with MeCP2 more in brain lysates of the SETD5 W834C/W834C mouse compared to wild-type. It should also be possible to test in their cell lines if MeCP2 comes down in SETD5 or ANKRD11 pulldowns, and then assess if TBLR1 mutations disrupt this pulldown (if it is detected). Further, if transcriptomic analysis is performed in the SETD5W834C/+ mouse (see above) these data could be interrogated to see if there are positive correlations in fold-changes with MeCP2 overexpression transcriptomic dysregulation or anti-correlations with MeCP2 loss of function effects. This analysis should be straightforward, as MeCP2 transcriptomic effects have been well curated in the published literature (e.g. Trostle et al. Int J Mol Sc 2023).

Minor Comments:

5.It would be relevant to interrogate the mass spec data from figure 4D to see if the NCoR1 interaction with ANKRD11 is affected by the SETD5 W834C mutation.

6.The number of DEGs identified in Ankrd11yod/yod ESCs from the RNA-seq analysis is extremely high (11063 total DEGs), which raises a concern about potential inflation of significance in the correlation analysis. The authors could apply a more stringent cutoff for defining DEGs and repeat the analysis to strengthen their claim.

7 Why did the authors use W834C mutant mice instead of mice with pathogenic missense mutations? Additional explanation for this choice is warranted.

8. In Figure 3a the truncation is listed at position 841, while the manuscript says 846.

9.The TBLR1 mutant self-association experiments (Sup. 1C) seem to show an effect for E63K and L83Q, with less TBLR1 self-association with both the WT and mutant copies, but the text says no effect was found. Could the authors comment on this discrepancy or clarify the analysis?

10.While the authors do explain the reasoning for grouping the two SETD5 mutant clones in their ESC RNA-seq analysis, it would be beneficial to include either a comparison of the two mutants, or an analysis using only one clone when comparing to the WT samples.

11.The authors spend substantial time in the introduction talking about the various clinical presentations of different mutations in TBLR1, implying that disruption of specific interactions by different types of mutations manifest in different underlying molecular pathology and that their findings will explain this variability. It might be helpful for the reader to include a model figure at the end of the study to synthesize their findings and at least speculate, based on their new work, how different mutations affect the relevant complexes and result in different phenotypes.

Reviewer #3

(Remarks to the Author)
In this manuscript by Paton et al, the authors report that TBLR1, a subunit of the NCoR corepressor complex encoded by TBL1XR1, serves as a molecular scaffold linking ANKRD11 and SETD5 to form a mammalian SET3-like (SET3C) transcriptional regulatory complex. The authors demonstrate that pathogenic variants in TBL1XR1, ANKRD11, and SETD5 disrupt assembly and function of this complex, revealing a shared molecular mechanism that underlies a group of neurodevelopmental disorders. The work combines biochemical interaction assays, genetic models, and transcriptomic analyses to establish the central role of SET3C in neuronal development. The manuscript is overall well written, the results are clearly presented, and the findings are innovative with good translational implications. However, enthusiasm for the manuscript is tempered by concerns regarding the validation of the Setd5 W834C mice and potential confounding effects of secondary genetic effects, limited characterization of the Setd5 W834C mice, and clarity of methods.
Major concerns:
1. Figure 1g, Figure 2b, Figure 2d, and Figure 3 - the inclusion of the corresponding quantitative data summarizing replicate IP results would strengthen the figures by showing the reproducibility of the findings across multiple technical replicates.

2. A subset of Setd5W834C/W834C homozygous mice survive and are fertile. The authors hypothesize that this rescue effect is due to a secondary genetic change as a cross between homozygous Setd5W834C/W834C and heterozygous Setd5W834C/+ mice produced a substantially higher proportion of homozygous pups. This raises the concern that findings in the homozygous Setd5W834C/W834C and heterozygous Setd5W834C/+ mice may be confounded by the secondary alterations, and it is difficult to determine that the single amino acid change preventing SETD5 from assembling into SET3C is the sole contributor or causative agent for the severe phenotypic consequences. The secondary alteration could also be disrupting the SET3C complex.

The manuscript’s methods would be strengthened by clarifying if the heterozygous Setd5W834C/+ mice backcrossed to mice from a stock center that controls for genetic drift, the number of generations backcrossed before generating the homozygous mice. Genome rearrangements and off target changes can occur in the genetic engineering process. The study would be further strengthened by screening for sequence alterations at predicted CRISPR/Cas9 off target sites or genome sequencing.

3. The Setd5 W834C/+ and Setd5 W834C/W834C mouse characterization would be benefit from a minimal behavioral or neuroanatomical study to strengthen the in vivo phenotypic correlation.

4. The generation of ANKRD11E2523K/E2523K homozygous ESCs are described in the Methods section but it is unclear where these ESCs are used experimentally in the manuscript. Furthermore, on page 7, the pathogenic C-terminal mutant forms of ANKRD11 studied via transient expression in HEK293 cells include E2522K. Therefore, is ANRKD11 E2523K a typo? Per the MANE transcript the residue in position 2523 should be an Arginine for ANRKD11.

5. Figure 4, please clarify if sex is included as a biological variable in the sample size and provide the male and female distribution.

6. Original uncropped Western blot images should be provided for all datasets as supplemental material.

Version 1:

Reviewer comments:

Reviewer #1

(Remarks to the Author)
I repeat my first conclusion that this is valuable work. Although I still disagree with the authors regarding the selection of a secondary mutation (very unlikely in my opinion), this is a small point against the many merits of their findings and manuscript.

Reviewer #2

(Remarks to the Author)
The authors have addressed several of my concerns, improving an already strong manuscript that will provide important insights for the field. The additional interaction and mass spectrometry data in the SETD5 and ANKRD11 mutants has sufficiently addressed the role of those proteins in SET3C stability, demonstrating that their interactions with TBLR1 do not influence the greater stability of NCoR.

Since an additional possibility for the function of SETD5 or ANKRD11 in SET3C is targeting NCoR to chromatin, I requested profiling of the genomic localization of SET3C components in the SETD5 or ANKRD11 mutants. However, I recognize that cofactor ChIP or CUT&RUN experiments could require substantial further work and are beyond the reasonable scope of this manuscript. Additionally, while it would have been informative to determine whether genes transcriptionally altered in SETD5 or ANKRD11 mutants exhibit concordant changes in histone acetylation or H3K36 methylation, I appreciate the authors' discussion of limitations in definitively establishing causality. The authors' acknowledgement of this limitation, together with the strong genetic and biochemical evidence presented, makes additional histone modification profiling non-essential for the current study.

The authors provide compelling evidence that the incorporation of SETD5 and ANKRD11 into SET3C is important for transcriptional regulation in their mutant cell lines, and I had requested this also be interrogated in the SETD5 W834C/+ mouse. In their rebuttal, the authors performed a robust characterization of gene expression changes in this mouse model. While there were fewer transcriptional changes in this more subtle SETD5 disruption, this is still an important dataset relevant to SETD5 function in the brain and I would request the authors incorporate this data into the study prior to publication.

Finally, the additional biochemical and rescue experiments adequately address the previously raised questions regarding functional interplay between MeCP2 and SETD5 in NCoR binding. While many mechanistic details remain to be explored in future studies, the current data are sufficient to demonstrate that while MeCP2 and SETD5 compete for TBLR1 binding, enhanced SET3C activity is unlikely to drive the primary phenotypes in Rett.

Overall, the additional experiments and clarifications have strengthened the study and addressed the major concerns raised in the initial review. I am pleased to recommend acceptance of the manuscript, subject to incorporation of the newly generated transcriptional data.

Reviewer #3

(Remarks to the Author)
In this revised manuscript by Paton et al, the authors report that TBLR1, a subunit of the NCoR corepressor complex encoded by TBL1XR1, serves as a molecular scaffold linking ANKRD11 and SETD5 to form a mammalian SET3-like (SET3C) transcriptional regulatory complex. The authors demonstrate that pathogenic variants in TBL1XR1, ANKRD11, and SETD5 disrupt assembly and function of this complex, revealing a shared molecular mechanism that underlies a group of neurodevelopmental disorders. The revised manuscript is strengthened with clarification regarding the validation of the Setd5 W834C mice, explanation of potential confounding effects of secondary genetic effects from a mixed background, and clarity of methods. There are two remaining concerns that if addressed would further strengthen the validation of the Setd5 W834C/+ mouse model and expand the discussion for future behavioral and neuroanatomical phenotyping of the Setd5 W834C/+ mouse model.

1. The revised manuscript is strengthened by the clarification regarding the Setd5 W834C/+ mouse allele generation and backcrossed generations. The additional information suggests that selection from genetic variation in the mixed background mice may have contributed to the reduced lethality penetrance in the Setd5 W834C/+ by Setd5 W834C/W834C cross. Genome rearrangements and off target changes can occur in the genetic engineering process and contribute to the genetic variation. The authors report that the guide RNAs used had 2 off-target sites in the exons with CFD scores >0.02 with 4 mismatches and 1-3 mismatches in the gRNA seed region. While an off-target mutation may be less likely due to the mismatches in the gRNA seed region, it would be beneficial to PCR amplify the genomic region of the two off-target sites and conduct Sanger sequencing to confirm these two sites are wildtype in the backcrossed Setd5 W834C/+ mice, particularly if the two off-target sites are in the same chromosome as Setd5 as they may co-segregate with the Setd5 W834C allele.

2. The authors provide limited phenotyping information regarding the Setd5 W834C/+ mice compared to wildtype, without notable differences in aggregate general health score, weight, and lifespan. It would be beneficial for the authors to remark in the discussion that future comprehensive behavioral and neuroanatomical study will strengthen the in vivo phenotypic

correlation and support translational research efforts.

Open Access This Peer Review File is licensed under a Creative Commons Attribution 4.0 International License, which permits use, sharing, adaptation, distribution and reproduction in any medium or format, as long as you give appropriate credit to the original author(s) and the source, provide a link to the Creative Commons license, and indicate if changes were made.
In cases where reviewers are anonymous, credit should be given to 'Anonymous Referee' and the source.
The images or other third party material in this Peer Review File are included in the article's Creative Commons license, unless indicated otherwise in a credit line to the material. If material is not included in the article's Creative Commons license and your intended use is not permitted by statutory regulation or exceeds the permitted use, you will need to obtain permission directly from the copyright holder.
To view a copy of this license, visit https://creativecommons.org/licenses/by/4.0/

We thank the reviewers for their positive remarks and constructive comments. In our revised manuscript we have added substantial data including mouse genetics, gene expression analyses and proteomics. We believe that these data strengthen our conclusions, extend our findings, and address many of the concerns raised by the reviewers. We hope that this improved version will now be suitable for publication.

Reviewer #1 (Remarks to the Author):

The accumulating identification of de novo mutations in various chromatin regulators as the basis for monogenic neurodevelopmental disorders, several which present overlapping phenotypes, potentially indicates functional commonalities and a deeper level of understanding that will illuminate future medical practice. The manuscript by Paton et al describes connections underpinning several monogenic neurodevelopmental disorders notably connecting known neurodevelopmental mutations in Ankrd11, Setd5 and Tblr5 into a mechanistic context. This is important work from one of the forefront laboratories that complements and extends ongoing progress in the field. The manuscript is lucid, thoroughly referenced and a pleasure to read.

A few minor comments –

1. Details of the transient expression methods used to evaluate protein-protein interactions are missing. There are several different ways to achieve transient expression and they have very different properties, mostly involving modest to extreme overexpression. If the authors used extreme overexpression, then many readers, this reviewer included, will be have skepticism skeptical evaluate these protein-protein interaction data with high. Given the other qualities of this manuscript, the skepticism is unlikely but nevertheless, the authors should accurately describe this aspect of their experiments.

We have revised the text throughout to provide more clarity about the transient protein expression methods used. Two methods were used: 1) tetracycline-induced expression of single copy TBLR1 cDNAs integrated at the specific FRT site-containing locus in Flp-In T-REx 293 cells, and 2) transfection of expression plasmids for tagged proteins into cells. In both cases, we harvested cells ~24 hours after transfection or tetracycline induction. The TREx system yields a modest overexpression and the transfection system yields a high overexpression.

2. The identified relationship between the mammalian and yeast SET3Cs is motivating but perhaps the mammalian version should be denoted as mSET3C, or something similar.

We appreciate the suggestion, however, we prefer to retain “SET3C” and qualify this with “mammalian” where it is necessary to avoid ambiguity. We believe this approach reflects the conserved nature of the complex and helps to simplify the nomenclature.

3. Line 256 3.6% of Setd5W834C/W834C survived and were fertile – and crossed to Setd5W834C/+, resulting in a higher (unspecified) survival rate, from which the authors conclude selection for a secondary mutation. It is very unlikely that selection for a secondary mutation could be achieved in inbred strains in mouse house crosses. More likely this is due to either epigenetic or incompletely penetrant mechanisms.

The survival rate is indicated in the plot (originally presented Supplementary Figure 4e). For clarity, we now explicitly specify the survival rate of this cross (and all other crosses) in the main text (page 9 line 277).

We agree that selection for a secondary mutation is unlikely in inbred mice. However, the SETD5$^{W834C/+}$ by SETD5$^{W834C/+}$ cross, which yielded 3.6% homozygous progeny, was between mice on a mixed background. These outbred mice were obtained by crossing wild-type C57BL/6J animals with chimeras derived from our targeted 129/Ola E14TG2a embryonic stem cells, and then crossing the resulting germline pups again with wild-type C57BL/6J mice. We therefore speculated that selection from this genetic variation might contribute to the reduced penetrance of *Setd5*$^{W834C/W834C}$ lethality seen in the subsequent *Setd5*$^{W834C/W834C}$ by *Setd5*$^{W834C/+}$ cross. We acknowledge that this information should have been explicitly included in the original version of the manuscript and have now added details of mouse genetic backgrounds to the Results (page 9 lines 260 and 279) and Methods (page 16 lines 499-500), and also to the figure legends (Figure 4b and Supplementary Figure 4e and f).

Given the discrepancy between the results of the *Setd5*$^{W834C/+}$ by *Setd5*$^{W834C/+}$ cross, and the *Setd5*$^{W834C/W834C}$ by *Setd5*$^{W834C/+}$ cross, we repeated the SETD5$^{W834C/+}$ by SETD5$^{W834C/+}$ cross using animals bred to a C57BL/6J background for >6 generations. We obtained no homozygous progeny out of a total of 31 offspring (new figure panel Supplementary Figure 4f) – a result which aligns well with the data from the original cross. This observation is consistent with the possibility that selection exploiting genetic variation present in outbred animals contributed to the reduced penetrance of the lethality seen in the *Setd5*$^{W834C/W834C}$ by *Setd5*$^{W834C/+}$ cross.

Since both the mixed background *Setd5*$^{W834C/+}$ by *Setd5*$^{W834C/+}$ cross, and the *Setd5*$^{W834C/W834C}$ by *Setd5*$^{W834C/+}$ cross were ongoing whilst the manuscript was in revision, we have now included more mice in these experiments (modified Figure 4b and Supplementary Figure 4e). The conclusions from these experiments are fully supported by this additional data.

4. Line 298 These data

We thank the reviewer for catching this mistake – we have corrected the error.

5. Line 315 – Ref 38 appears mistakenly cited
Data from the 100k genome project places ANKRD11, SETD5 and MECP2 within the top ten most frequently mutated genes contributing to monogenic neurodevelopmental abnormalities 38.

The focus of this reference is the small nuclear RNA RNU2-2, but it is cited for its analysis of the prevalence of mutations in different genes in neurodevelopmental disorders in the 100,000 genomes project (Figure 2 in the cited paper).

Reviewer #2 (Remarks to the Author):

In this study Paton, Bird, Lyst and colleagues investigate developmental disorder associated gene mutations that disrupt chromatin protein interactions to define new connections between disorders. They characterize a putative functional homolog of the yeast SET3C chromatin regulatory complex that is composed of ANKRD11 and SETD5 interacting with the NCoR corepressor complex through the TBLR1 subunit. Building off previous findings suggesting that these proteins interact and genetic studies showing the mutations in each of these proteins cause related developmental disorders, the authors characterize the effects of missense mutations in TBLR1 on interactions with SETD5 and ANKRD11. They show that mutations in the N-terminal domain of TBLR1 disrupt ANKDR11 association while mutations in the C-terminal WDR domain disrupt SETD5 association. They then show that disease-associated mutations in the C-terminal domain of ANKRD11 disrupt interaction with TBLR1. Turning to SETD5, they find that it interacts with TBLR1 through a peptide sequence similar to a core TBLR1-binding sequence of the Rett syndrome protein MeCP2, and show that disease-associated mutations in this sequence disrupt the SETD5-TBLR1 interaction. To further investigate the SETD5-TBLR1 interaction they generate and study a SETD5 W834C mutant mouse, showing that this mutation disrupts TBLR1 interaction in vivo and leads to disease relevant phenotypes. Finally, to examine the functional impact of disrupting the putative ANKRD11/SETD5/NCoR complex the authors perform RNA-seq on ES cells with either SETD5 or ANKRD11 mutations that disrupt NCoR binding and analyze existing perturb-seq data. They find highly overlapping effects for disruption of ANKRD11, SETD5, and NCoR components, supporting the notion that these proteins function in an overlapping pathway.

Overall, this study does an impressive job of establishing interactions in the putative mammalian SET3C complex and dissecting the effects of disease mutations on these interactions. This work defines a new group of related disorders that impact shared chromatin biology. The experiments are rigorously executed, and the findings are convincing. The study identifies molecular interactions between distinct genetic causes of neurodevelopmental diseases, beginning to define generalized disease-associated pathways that the field is searching for. It provides timely and impactful findings that will be of interest to many investigators and the focus of future studies across numerous labs.

As it is currently presented however, the study misses some chances to fully investigate the interactions of the putative SET3C complex and does not provide insight into the mechanisms leading to transcriptional disruption occurring in mutants in vivo. Further, the study posits addressable questions about the interplay between MeCP2 and this complex, but does not include analyses to address these questions.

In my opinion, some or all of these gaps should be addressed before the manuscript is ready for publication in Nature Communications.
Major issues:

1.The authors propose that SETD5 and ANKRD11 work together with NCoR as part of a mammalian “SET3C” complex, but additional experiments to test the roles of SETD5 and ANKRD11 in this complex are warranted. The authors could use their cell lines or mutant constructs to determine whether mutations affecting SETD5 and ANKRD11 association with NCoR disrupts either the protein levels of NCoR components or NCoR intra-complex interactions. This would determine whether SETD5 and/or ANKRD11 function to stabilize or scaffold the SET3C complex, respectively. An additional possibility would be that SETD5 and/or ANKRD11 impact the recruitment of SET3C to chromatin, which could be tested using ChIP-seq targeting NCoR components in SETD5 and ANKRD11 mutant cell lines, or in the SETD5 W834C mouse. Evaluating whether the NCoR complex remains intact after disease-associated mutations in SETD5 or ANKRD11 would provide clearer insight into the contribution of these proteins to the mammalian SET3C complex.

The effects of the *SETD5*^{W834C} mutation on NCoR complex levels and intra-complex interactions are shown in Figure 4d, where NCOR1 is immunoprecipitated from wild-type and *SETD5*$^{W834C/W834C}$ mouse brain extracts. Whereas SETD5 binding to NCOR1 is strongly reduced in the SETD5$^{W834C/W834C}$ samples, association of NCOR1 with all other NCoR complex components is unaffected. We have now emphasised this conclusion by stating in the text (page 9, lines 268-272) that the integrity of the rest of the SET3 complex is unaffected by the W834C mutation this experiment.

To further test the effects of ANKRD11 or SETD5 mutations on NCoR intra-complex interactions, we have now performed IPs of GFP-ANKRD11 expressed in our SETD5$^{W834C/W834C}$ ES cells (new data Supplementary Figure 4c), and of mCherry-SETD5 expressed in a newly generated ANKRD11 null TREx cell line (new data Figure 2e). In all cases, we found that the rest of the SET3/NCoR complex is unaffected by disruption of ANKRD11 or SETD5 binding. The IP-mass spectrometry of mCherry-SETD5 expressed in ANKRD11 null cells (new data Figure 2e) and mass spectrometry on extracts from SETD5 mutant ESCs (reanalysed data Supplementary Figure 4d) both confirm that mutations in these proteins do not affect the levels of other members of the complex. Thus ANKRD11 and SETD5 do not appear to be required for the stability of other members of the SET3 complex, or for NCoR intra-complex interactions. This new data is described in the Results section (page 7 204-207 and page 9 lines 268-272). Please see below for discussion of the possibility of NCoR/SET3 ChIP-seq.

2.The direct mechanism and function of SET3C-like complex in mammalian models is not addressed, despite testable hypotheses stemming from what is known about the components of the complex. Does SET3C complex in mammals show a molecular function comparable to what is known in the yeast? The authors should be

able investigate these mechanisms to understand how transcriptional disruption occurs when SET3C components are mutated. For example, disruption of SETD5 has been shown to have effects on H3K36 methylation-associated transcriptional regulation, and NCoR is well established to function as a histone deacetylase, but the authors don't perform complementary histone modification profiling experiments to investigate these marks. In addition, they do not attempt ChIP-seq with antibodies to NCoR or members of the SET3C complex to investigate if changes in their recruitment to chromatin underlie a subset of transcriptomic effects. This work could be done in the cell lines they have performed RNA-seq on, or, even better, in the brain tissue of the SETD5W834C/+ mouse. In addition to providing mechanism, the results could address which effects on transcription may directly result from loss of complex function.

We thank the reviewer for suggesting these interesting lines of research. We agree that there are testable hypotheses about the mechanism and function of SET3C. As the reviewer notes, SETD5 has been linked to effects on H3K36 methylation, and the NCoR complex contains the histone deacetylase HDAC3. However, while profiling H3K36 methylation and histone acetylation would provide some useful information, this alone would not establish whether observed changes are a cause or consequence of transcriptional perturbation following SET3C disruption. Resolving this question will require generation and characterization of cell lines with inducible degrons - to allow acute depletion of SET3 components - followed by time resolved profiling of histone modifications and the nascent transcriptome. We have initiated such experiments, however, the establishment and validation of appropriate systems, as well as the execution of temporally resolved assays, will likely span many months at least.

We also agree that genome binding analyses of SET3C components would strengthen our study, and we have made substantial efforts towards this goal. Given our previous observation that formaldehyde cross-linking destroys the interaction between TBLR1 and the NID of MeCP2 (Lyst et al, 2013), we opted to use CUT&RUN rather than ChIP-seq, since standard CUT&RUN protocols do not require crosslinking. To this end, we generated a cell line stably expressing GFP-ANKRD11, and we also raised a custom antibody against ANKRD11. Unfortunately, experiments using these reagents both failed to yield signal above the background levels detected in negative controls (cells lacking the tag or ANKRD11 knockout cells), despite robust, specific signal in positive control assays (histone H3 lysine K4 trimethylation). We are continuing to explore alternative strategies for profiling chromatin association of the complex, but these technical constraints have thus far limited our ability to address this question. Consistent with the possibility that genome binding profiles of SET3C are not readily accessible using standard methods, we note that there is contradictory data in high profile papers concerning the genome binding profile of SETD5. For example, in one study SETD5 is reported to bind predominantly to transcription start sites and active enhancers (Deliu et al, 2018; Nature Neuroscience), whereas in another it is found associated with active gene bodies (Sessa et al, 2019; Neuron).

We therefore respectfully submit that the full set of experiments required to rigorously investigate these questions would represent a major new study. We hope the reviewer will agree that pursuing these lines of research is a logical direction for future work rather than a prerequisite for publication of the current manuscript.

3.While the study uses robust assays in cell lines to assess interactions and transcriptomic effects, the follow up in vivo is limited, making the study's conclusions about impact on biology less robust. The study would be strengthened if it included transcriptomic analysis from brain tissue from the newly generated SETD5W834C/+ mouse. These data could be used to probe the biological consequences of disrupt SET3C and also be utilized in mechanistic studies (see comment above) and interrogation of MECP2 interactions (see below).

We have performed RNA-seq on brain cortical tissue from 4-week-old *Setd5*$^{W834C/+}$ and *Setd5*$^{+/+}$ mice. We sequenced at least 80 million reads from 6 biological replicates (3 females and 3 males) per genotype. We saw very few gene expression changes (Reviewer Figure 1), with more variation observed between sexes than between genotypes. As SETD5 is essential for embryonic development, we hypothesise that more substantial effects on gene expression would be observed earlier in development. Consistent with this, our *Setd5*$^{W834C/W834C}$ homozygous ES cells do indeed show irregular gene expression patterns. Future studies including transcriptomics on different brain regions and neuronal subtypes, at early developmental timepoints, will likely be required to fully elucidate the role of SET3C-mediated transcriptional regulation in neurodevelopment. The *Setd5*$^{W834C/+}$ RNA-seq data is shown below (Reviewer Figure 1), but is not included in the revised version of the manuscript.

[editorial note: figure redacted]

4.The authors propose that competition for TBLR1 binding between MeCP2 and SETD5 may occur, but they don't investigate this possibility using existing data or perform tractable experiments to test this possibility. For example, the authors could examine the mass spec data from Figure 4D to see if NCoR1 interacts with MeCP2 more in brain lysates of the SETD5 W834C/W834C mouse compared to wild-type. It should also be possible to test in their cell lines if MeCP2 comes down in SETD5 or ANKRD11 pulldowns, and then assess if TBLR1 mutations disrupt this pulldown (if it is detected). Further, if transcriptomic analysis is performed in the SETD5W834C/+ mouse (see above) these data could be interrogated to see if there are positive correlations in fold-changes with MeCP2 overexpression transcriptomic dysregulation or anti-correlations with MeCP2 loss of function effects. This analysis should be straightforward, as MeCP2 transcriptomic effects have been well curated in the published literature (e.g. Trostle et al. Int J Mol Sc 2023).

We appreciate the suggestion to investigate the possibility of competition for TBLR1 binding between SETD5 and MeCP2. We have explored this question with new

experiments in a section titled “SETD5 mutation does not rescue the RTT-like phenotypes of MeCP2 null mice” (page 10 lines 289-311). Using co-IP assays, we found that SETD5 and MeCP2 can indeed compete for binding to TBLR1 and the NCoR complex (new data Figure 5a). To test whether this competition might be functional, we crossed *Mecp2-null* mice to *Setd5*^{W834C} mice and asked whether the inability of SETD5 to bind to NCoR rescues the Rett-like phenotypes and survival (new data Figure 5b-d). A potential consequence of competition could be MeCP2, a highly abundant neuronal protein, sequestering TBLR1 and associated proteins away from SETD5. In this scenario, some of the phenotypic deficits observed in Rett syndrome would be due to unrestrained SET3C activity, that would normally be tempered by MeCP2. However, we observed no change in the trajectory of the Rett-like phenotype, and no extended survival in *Mecp2*$^{-/y}$,*Setd5*$^{W834C/+}$ mice. This result offers no support for the notion that competition underlies aspects of the Rett-like phenotype. We explain this point in our Discussion (page 13 lines 390-399). As outlined above, the gene expression changes in *Setd5*$^{W834C/+}$ mouse brain are extremely subtle (only 14 differentially expressed genes with padj < 0.05), and they show no clear relationship with the thousands of differentially expressed genes reported in MeCP2 mutants.

Minor Comments:

5.It would be relevant to interrogate the mass spec data from figure 4D to see if the NCoR1 interaction with ANKRD11 is affected by the SETD5 W834C mutation.

After NCoR1 IP, ANKRD11 is detected in all three wild-type samples, and also at similar levels in two out of the three SETD5 W834C mutant samples. However, ANKRD11 was not detected in the third mutant sample. We strongly suspect that ANKRD11 is “missing at random” from this sample (since there are only 762 proteins identified in this sample as compared with over >2200 proteins identified in the other samples), and that ANKRD11 binding to the NCoR complex is therefore not affected by the SETD5 W834C mutation. However, given that the data on ANKRD11 is not conclusive in this experiment, we prefer not to include these data points in our plot. Instead, to address the same question, we have shown that the SETD5 W834C mutation does not affect ANKRD11 binding to the NCoR complex through a new experiment where we perform IPs of GFP-ANKRD11 expressed in SETD5$^{W834C/W834C}$ ESCs. In this experiment, we observe that the SETD5 W834C mutation, which prevents association of SETD5 with SET3C, does not affect ANKRD11 binding to other SET3/NCoR complex components (new data Supplementary Figure 4c) (Results section page 9 lines 268-272).

6.The number of DEGs identified in Ankrd11yod/yod ESCs from the RNA-seq analysis is extremely high (11063 total DEGs), which raises a concern about potential inflation of significance in the correlation analysis. The authors could apply a more stringent cutoff for defining DEGs and repeat the analysis to strengthen their claim.

We have repeated our analysis using a more stringent p-value (padj < 0.01) for the definition of DEGs and we find that the calculated correlation coefficients are not substantially affected (R = 0.87 as compared with R = 0.85) (Supplementary Figure 5d) (Results section page 11 lines 334-336).

7 Why did the authors use W834C mutant mice instead of mice with pathogenic missense mutations? Additional explanation for this choice is warranted.

When we initiated our gene-targeting experiments, pathogenic missense mutations within the SETD5 NID had not yet been reported in the ClinVar or SFARI databases. These entries only became available later in in 2022–2023. We chose the W834C mutation because the corresponding residue in MeCP2 (R306) is known to be critical for TBLR1 binding, and its mutation (R306C) causes Rett syndrome without destabilizing the MeCP2 protein. We have added this last point of explanation to the text (page 8 line 224).

8. In Figure 3a the truncation is listed at position 841, while the manuscript says 846.

Thanks for catching this mistake – we have corrected the error.

9.The TBLR1 mutant self-association experiments (Sup. 1C) seem to show an effect for E63K and L83Q, with less TBLR1 self-association with both the WT and mutant copies, but the text says no effect was found. Could the authors comment on this discrepancy or clarify the analysis?

We have repeated this experiment, and the results of the replicate experiment (see source data for Supplementary Figure 1c) suggest there is no effect of the E63K and L83Q mutations on TBLR1 self-association. We believe that the apparent effect on binding in the originally presented figure was likely due to weaker expression of the bait protein in those samples. Since our assays cannot rule out subtle effects on binding, we have changed the text to say that “TBLR1 self-association was not *substantially* affected by these mutations” (page 5 line 139).

10.While the authors do explain the reasoning for grouping the two SETD5 mutant clones in their ESC RNA-seq analysis, it would be beneficial to include either a comparison of the two mutants, or an analysis using only one clone when comparing to the WT samples.

We have now extended out analysis to include one comparison based on the two $SETD5^{W834C/W834C}$ lines (new Supplementary Figure 5e), and another based on the sole $SETD5^{\Delta KWK/\Delta KWK}$ line (new Supplementary Figure 5f). The results are consistent with the originally presented grouped data (Figure 6c) and are described in the Results section (page 11 lines 334 – 336).

11.The authors spend substantial time in the introduction talking about the various clinical presentations of different mutations in TBLR1, implying that disruption of specific interactions by different types of mutations manifest in different underlying molecular pathology and that their findings will explain this variability. It might be helpful for the reader to include a model figure at the end of the study to synthesize their findings and at least speculate, based on their new work, how different mutations affect the relevant complexes and result in different phenotypes.

We thank the reviewer for this thought-provoking comment, which prompted us to reconsider the wording of the introduction. Detailing the clinical manifestations of different TBLR1 mutations could suggest that we aim to draw strong conclusions about genotype–phenotype relationships. Given the limited clinical data and the small number of reported cases – often a single case per syndrome – we believe there is not sufficient evidence to make such claims. We have therefore revised the introduction (page 3 lines 55-61) to emphasize the complexity of TBLR1-associated clinical presentations without linking specific mutations or protein domains to distinct clinical outcomes. For the aforementioned reasons, we also prefer not to add more detail to our existing model figure (Figure 6h).

Reviewer #3 (Remarks to the Author):

In this manuscript by Paton et al, the authors report that TBLR1, a subunit of the NCoR corepressor complex encoded by TBL1XR1, serves as a molecular scaffold linking ANKRD11 and SETD5 to form a mammalian SET3-like (SET3C) transcriptional regulatory complex. The authors demonstrate that pathogenic variants in TBL1XR1, ANKRD11, and SETD5 disrupt assembly and function of this complex, revealing a shared molecular mechanism that underlies a group of neurodevelopmental disorders. The work combines biochemical interaction assays, genetic models, and transcriptomic analyses to establish the central role of SET3C in neuronal development. The manuscript is overall well written, the results are clearly presented, and the findings are innovative with good translational implications. However, enthusiasm for the manuscript is tempered by concerns regarding the validation of the Setd5 W834C mice and potential confounding effects of secondary genetic effects, limited characterization of the Setd5 W834C mice, and clarity of methods.

Major concerns:

1. Figure 1g, Figure 2b, Figure 2d, and Figure 3 - the inclusion of the corresponding quantitative data summarizing replicate IP results would strengthen the figures by showing the reproducibility of the findings across multiple technical replicates.

Many of our findings are supported by quantitative mass spectrometry performed using replicates from independent cell transfections and IPs. Wherever our main

findings rely exclusively on IP followed by Western blot (Figure 1g, 2b, 3e, 3g, 3h, 5a, Supplementary Figure 1c, 4c), we now present data from replicate experiments in the source data (see “formatted western blots_replicate experiments doc.”)

2. A subset of Setd5W834C/W834C homozygous mice survive and are fertile. The authors hypothesize that this rescue effect is due to a secondary genetic change as a cross between homozygous Setd5W834C/W834C and heterozygous Setd5W834C/+ mice produced a substantially higher proportion of homozygous pups. This raises the concern that findings in the homozygous Setd5W834C/W834C and heterozygous Setd5W834C/+ mice may be confounded by the secondary alterations, and it is difficult to determine that the single amino acid change preventing SETD5 from assembling into SET3C is the sole contributor or causative agent for the severe phenotypic consequences. The secondary alteration could also be disrupting the SET3C complex.

The manuscript’s methods would be strengthened by clarifying if the heterozygous Setd5W834C/+ mice backcrossed to mice from a stock center that controls for genetic drift, the number of generations backcrossed before generating the homozygous mice. Genome rearrangements and off target changes can occur in the genetic engineering process. The study would be further strengthened by screening for sequence alterations at predicted CRISPR/Cas9 off target sites or genome sequencing.

We thank the reviewer for this constructive remark. The mice used in our initial *Setd5*$^{W834C/+}$ by *Setd5*$^{W834C/+}$ cross were on a mixed background generated by mating wild-type C57BL/6J mice with chimeras derived from our targeted 129/Ola E14TG2a embryonic stem cells, and then crossing the resulting germline pups again with wild-type C57BL/6J mice. We have now clarified these details in the Results (page 9 line 260) and Methods (page 16 lines 499-500). To address the concern that our findings may have been confounded by secondary genetic alterations, we have repeated this cross using animals backcrossed to a C57BL/6J background for at least 6 generations (new data Supplementary Figure 4f). If our original finding was confounded by a secondary lethal mutation present in the mixed background animals, then we would expect 25% of the surviving pups from the subsequent *Setd5*$^{W834C/+}$ by *Setd5*$^{W834C/+}$ inbred cross to be homozygous for the W834C mutation. In fact, this new cross produced no homozygous mutant progeny (out of a total 31 offspring). This result aligns well with the outcome of the first experiment, and reinforces the conclusion that the W834C mutation has severe consequences in homozygotes. Given that the two *Setd5*$^{W834C/+}$ by *Setd5*$^{W834C/+}$ crosses, separated by over 6 generations of breeding, have both produced high levels of lethality in homozygotes, we believe it is highly unlikely that this effect is due to a confounding secondary mutation. The Results section discusses the possibility that genetic variation in the mixed-background animals contributed to the reduced penetrance of embryonic lethality in *Setd5*$^{W834C/W834C}$ homozygotes (page 10 lines 281-284).

The guide RNAs used to make the *Setd5*^{W834C} mouse line had high MIT/CFD specificity scores (MIT > 80 and CFD = 91) (calculated using the CRISPOR.org web tool). There are only 2 off-target sites in exons with CFD scores >0.02 (sites with CFD scores <0.02 are unlikely to be cleaved according to the CRISPOR manual). These sites both have 4 mismatches with 1-3 mismatches in the gRNA seed region.

We hope the reviewer will agree that our conclusions about the *Setd5*^{W834C} allele are now based on robust and validated genetic evidence.

3. The Setd5 W834C/+ and Setd5 W834C/W834C mouse characterization would be benefit from a minimal behavioral or neuroanatomical study to strengthen the in vivo phenotypic correlation.

We appreciate the suggestion to better characterize our *Setd5*^{W834C} mice at the behavioural or neuroanatomical level. To address this issue, we have performed an observational assessment of six parameters reflecting neurological function - mobility, gait, hindlimb clasping, tremor, breathing, and general condition - in *Setd5*$^{W834C/+}$ mice. In these assessments, *Setd5*$^{W834C/+}$ animals were indistinguishable from wild-type controls (new data Figure 5b-d), suggesting that any behavioural/neurological phenotypes were mild. We did not score *Setd5*$^{W834C/W834C}$ homozygous mice for neurological phenotypes due to difficulty in establishing a cohort and inability to score them blind (due to their reduced size compared to littermates). A future more comprehensive behavioural and neuroanatomical analysis of our *Setd5*^{W834C} animals, especially in comparison to genetic models of related disorders, promises to yield valuable insights into the broader functional consequences of SET3C perturbation. This work will, however, require substantial additional breeding and resources, which we hope the reviewer will agree is beyond the scope of the present paper.

4. The generation of ANKRD11E2523K/E2523K homozygous ESCs are described in the Methods section but it is unclear where these ESCs are used experimentally in the manuscript. Furthermore, on page 7, the pathogenic C-terminal mutant forms of ANKRD11 studied via transient expression in HEK293 cells include E2522K. Therefore, is ANRKD11 E2523K a typo? Per the MANE transcript the residue in position 2523 should be an Arginine for ANRKD11.

The mouse ANKRD11 E2523K mutation (found in the yoda mouse strain and which we introduced into mouse ESCs) is equivalent to the human ANKRD11 E2522K patient mutation (which we expressed in HEK293 cells). The E2523K ES cells are used for the RNA-seq (Figure 6a-c and Supplementary Figure 5d-g). For clarity, we have edited the Results (page 11 lines 323 - 324) to explicitly explain the relationships between the mouse and human residues and mutations.

5. Figure 4, please clarify if sex is included as a biological variable in the sample size and provide the male and female distribution.

We now show the male and female distributions for each experiment using shape-coding or stacked bar charts, and the numbers of each sex are stated in the figure

legends. For brain and body weight measurements, values were normalised to the average of the wild-type littermates of the same sex (modified Figure 4a and 4c and Supplementary Figure 4b).

6. Original uncropped Western blot images should be provided for all datasets as supplemental material.

We have supplied the raw data for all Western blot images in Source data.

We again thank the reviewers for their positive remarks and constructive comments. In our revised manuscript we have added the new data and discussion points requested by the reviewers. We hope that this improved version will now be suitable for publication in Nature Communications.

REVIEWER COMMENTS

Reviewer #1 (Remarks to the Author):

I repeat my first conclusion that this is valuable work. Although I still disagree with the authors regarding the selection of a secondary mutation (very unlikely in my opinion), this is a small point against the many merits of their findings and manuscript.

We thank the reviewer for their positive remarks and for their continued efforts in evaluating our manuscript.

Reviewer #2 (Remarks to the Author):

The authors have addressed several of my concerns, improving an already strong manuscript that will provide important insights for the field. The additional interaction and mass spectrometry data in the SETD5 and ANKRD11 mutants has sufficiently addressed the role of those proteins in SET3C stability, demonstrating that their interactions with TBLR1 do not influence the greater stability of NCoR.

Since an additional possibility for the function of SETD5 or ANKRD11 in SET3C is targeting NCoR to chromatin, I requested profiling of the genomic localization of SET3C components in the SETD5 or ANKRD11 mutants. However, I recognize that cofactor ChIP or CUT&RUN experiments could require substantial further work and are beyond the reasonable scope of this manuscript. Additionally, while it would have been informative to determine whether genes transcriptionally altered in SETD5 or ANKRD11 mutants exhibit concordant changes in histone acetylation or H3K36 methylation, I appreciate the authors' discussion of limitations in definitively establishing causality. The authors' acknowledgement of this limitation, together with the strong genetic and biochemical evidence presented, makes additional histone modification profiling non-essential for the current study.

The authors provide compelling evidence that the incorporation of SETD5 and ANKRD11 into SET3C is important for transcriptional regulation in their mutant cell lines, and I had requested this also be interrogated in the SETD5 W834C/+ mouse. In their rebuttal, the authors performed a robust characterization of gene expression changes in this mouse model. While there were fewer transcriptional changes in this

more subtle SETD5 disruption, this is still an important dataset relevant to SETD5 function in the brain and I would request the authors incorporate this data into the study prior to publication.

Finally, the additional biochemical and rescue experiments adequately address the previously raised questions regarding functional interplay between MeCP2 and SETD5 in NCoR binding. While many mechanistic details remain to be explored in future studies, the current data are sufficient to demonstrate that while MeCP2 and SETD5 compete for TBLR1 binding, enhanced SET3C activity is unlikely to drive the primary phenotypes in Rett.

Overall, the additional experiments and clarifications have strengthened the study and addressed the major concerns raised in the initial review. I am pleased to recommend acceptance of the manuscript, subject to incorporation of the newly generated transcriptional data.

We thank the reviewer for their positive remarks and for their continued efforts in evaluating our manuscript.

As requested by the reviewer, we have now incorporated the *Setd5*^{W834C} RNA-seq data set (page 9 lines 249-254 and Supplementary Figure 4C).

Reviewer #3 (Remarks to the Author):

In this revised manuscript by Paton et al, the authors report that TBLR1, a subunit of the NCoR corepressor complex encoded by TBL1XR1, serves as a molecular scaffold linking ANKRD11 and SETD5 to form a mammalian SET3-like (SET3C) transcriptional regulatory complex. The authors demonstrate that pathogenic variants in TBL1XR1, ANKRD11, and SETD5 disrupt assembly and function of this complex, revealing a shared molecular mechanism that underlies a group of neurodevelopmental disorders. The revised manuscript is strengthened with clarification regarding the validation of the Setd5 W834C mice, explanation of potential confounding effects of secondary genetic effects from a mixed background, and clarity of methods. There are two remaining concerns that if addressed would further strengthen the validation of the Setd5 W834C/+ mouse model and expand the discussion for future behavioral and neuroanatomical phenotyping of the Setd5 W834C/+ mouse model.

We thank the reviewer for their positive remarks and for their continued efforts in evaluating our manuscript.

1. The revised manuscript is strengthened by the clarification regarding the Setd5 W834C/+ mouse allele generation and backcrossed generations. The additional

information suggests that selection from genetic variation in the mixed background mice may have contributed to the reduced lethality penetrance in the Setd5 W834C/+ by Setd5 W834C/W834C cross. Genome rearrangements and off target changes can occur in the genetic engineering process and contribute to the genetic variation. The authors report that the guide RNAs used had 2 off-target sites in the exons with CFD scores >0.02 with 4 mismatches and 1-3 mismatches in the gRNA seed region. While an off-target mutation may be less likely due to the mismatches in the gRNA seed region, it would be beneficial to PCR amplify the genomic region of the two off-target sites and conduct Sanger sequencing to confirm these two sites are wildtype in the backcrossed Setd5 W834C/+ mice, particularly if the two off-target sites are in the same chromosome as Setd5 as they may co-segregate with the Setd5 W834C allele.

We performed PCR and sequencing of the relevant off target sites in our mice and in the ES cells from which the mice were originally derived. The data confirms that the sites are intact and were unaffected by our CRISPR targeting (Methods page 16 lines 458-460 and Source Data).

2. The authors provide limited phenotyping information regarding the Setd5 W834C/+ mice compared to wildtype, without notable differences in aggregate general health score, weight, and lifespan. It would be beneficial for the authors to remark in the discussion that future comprehensive behavioral and neuroanatomical study will strengthen the in vivo phenotypic correlation and support translational research efforts.

We have expanded our discussion to include consideration of future behavioural and neuroanatomical studies of our mice (page 12 lines 359-363).
